# Synthesis, Stability, and Biological Evaluation of Novel Aminoderivatives Incorporating the Aza-Acridine Scaffold

**DOI:** 10.3390/molecules30122612

**Published:** 2025-06-16

**Authors:** Maria Karelou, Anthi Panara, Eleftheria Chatziorfanou, Aikaterini F. Giannopoulou, Dimitrios J. Stravopodis, Evagelos Gikas, Ioannis K. Kostakis

**Affiliations:** 1 Department of Pharmacy, Division of Pharmaceutical Chemistry, National and Kapodistrian University of Athens, Panepistimiopolis, Zografou, 15771 Athens, Greece; marykar@pharm.uoa.gr (M.K.); chatzior@pharm.uoa.gr (E.C.); 2 Laboratory of Analytical Chemistry, Department of Chemistry, National and Kapodistrian University of Athens, Panepistimiopolis, Zografou, 15771 Athens, Greece; panaranthi@chem.uoa.gr (A.P.); vgikas@chem.uoa.gr (E.G.); 3 Laboratory of Cellular Oncology, Section of Cell Biology and Biophysics, Department of Biology, School of Science, National and Kapodistrian University of Athens, Panepistimiopolis, Zografou, 15771 Athens, Greece; aigiann@biol.uoa.gr (A.F.G.); dstravop@biol.uoa.gr (D.J.S.)

**Keywords:** acridines, cancer, computational chemistry, cytotoxic assay, drug discovery, mass spectrometry, stability

## Abstract

Several new amino-substituted aza-acridine derivatives bearing one or two basic side chains have been designed and synthesized. Their anticancer activities were evaluated in vitro against two human cancer cell lines: T24 (urothelial bladder carcinoma, malignancy grade III) and WM266-4 (metastatic melanoma). Some of the synthesized compounds induced significant antiproliferative effects, with WM266-4 cells appearing more susceptible than T24 cells. This apparent cell-type selectivity may reflect differences in the mutational profiles and molecular target landscapes between the two cancer models. A stability study under hydrolytic conditions, based on a validated method, indicated that the most active compounds were stable under aqueous conditions. Computational analysis further supported the stability of these analogs, providing insights into the structure–stability relationships of the synthesized compounds.

## 1. Introduction

Acridine derivatives have been extensively studied as potential anticancer agents due to their unique structural and pharmacological properties [1,2,3,4]. Acridine serves as a versatile scaffold for the synthesis of analogs with a broad spectrum of biological activities, including anticancer [5,6], anti-inflammatory [7], antiviral [8], antimalarial [9,10], and antiparasitic effects [11]. The π-conjugated planar structure enhances hydrophobicity, facilitating interactions with biomolecular targets and DNA intercalation, which inhibits replication in rapidly dividing cancer cells. In addition to DNA intercalation, acridine derivatives inhibit critical enzymes, such as topoisomerases [12,13,14], telomerase [15,16], and cyclin-dependent kinases [17], contributing significantly to their anticancer effects.

**Figure 1:**
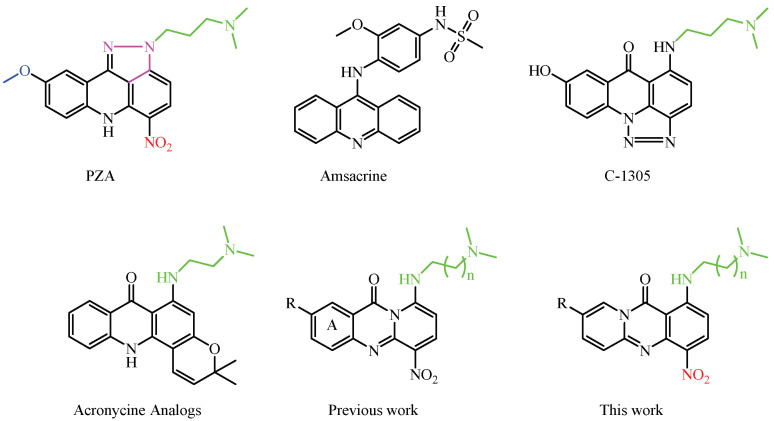
Representative compounds illustrating key structural features and those prepared by our group. Color coding highlights: 7-methoxy group (blue), nitro group (red), basic side chains (green), and fused pyrazole ring (purple).

Notable examples include pyrazoloacridines [18,19,20,21], thiadiazinoacridines [22], triazoloacridones [22,23,24], and imidazoloacridones. Among them, compounds like pyrazoloacridine (PZA), imidazoloacridine C-1311, acridine carboxamide DACA, and aza-acridines have attracted significant attention due to their promising biological properties. Structure-activity relationship (SAR) studies have highlighted that specific features, such as a 7-methoxy group, an electron-deficient nitro group, and one or two basic side chains, are crucial for both activity and selectivity, as illustrated in Figure 1, where key features are color-coded for clarity. Optimal activity is observed with side chains comprising two or three methylene units, whereas modifications that reduce the hydrogen-bonding ability of the amino group markedly diminish efficacy, emphasizing the importance of these interactions. The incorporation of a carbonyl group between the amino side chain and the chromophore improves the flexibility and spatial arrangement, while the addition of a fused pyrazole group further improves the activity [18,19,20,21,22,23,24].

As part of an extensive research program on the synthesis and pharmacological evaluation of acridine analogs [25,26,27,28], we recently reported the synthesis of several amino-substituted aza-acridines bearing a nitro group at C-4.

**Scheme 1:**
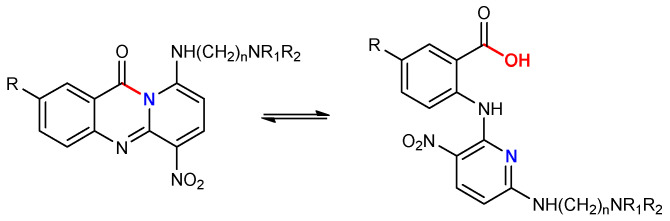
Hydrolysis of the compounds.

Although these compounds exhibit low cytotoxic activity, this limitation is attributed to their hydrolytic instability in aqueous environments. Aza-acridines are amenable to ring-opening reactions, and their stability is strongly influenced by the substitution pattern on the core structure [26]. Preliminary findings suggest that electron-donating groups enhance core stability, potentially mitigating hydrolytic degradation and preserving biological activity (Scheme 1).

Prompted by these findings, we investigated this scaffold further and studied the influence of nitrogen positioning and substituent patterns on scaffold stability. In the new derivatives, nitrogen was relocated to the opposite side of the carbonyl group, while a nitro group or a second basic side chain was incorporated into the A-ring. The rationale for these modifications was based on previous findings of our group, which indicated that the electron density distribution around the carbonyl group plays a crucial role in the hydrolytic stability of the heterocyclic ring [26]. Specifically, the introduction of electron-donating or electron-withdrawing groups in proximity to the carbonyl can significantly affect the susceptibility of the ring to nucleophilic attack and ring opening. Thus, the newly introduced structural changes were designed to modulate the electronic environment of the core and enhance stability while preserving or improving the pharmacological profile observed in related acridines.

## 2. Results and Discussion

### 2.1. Chemistry

**Scheme 2:**
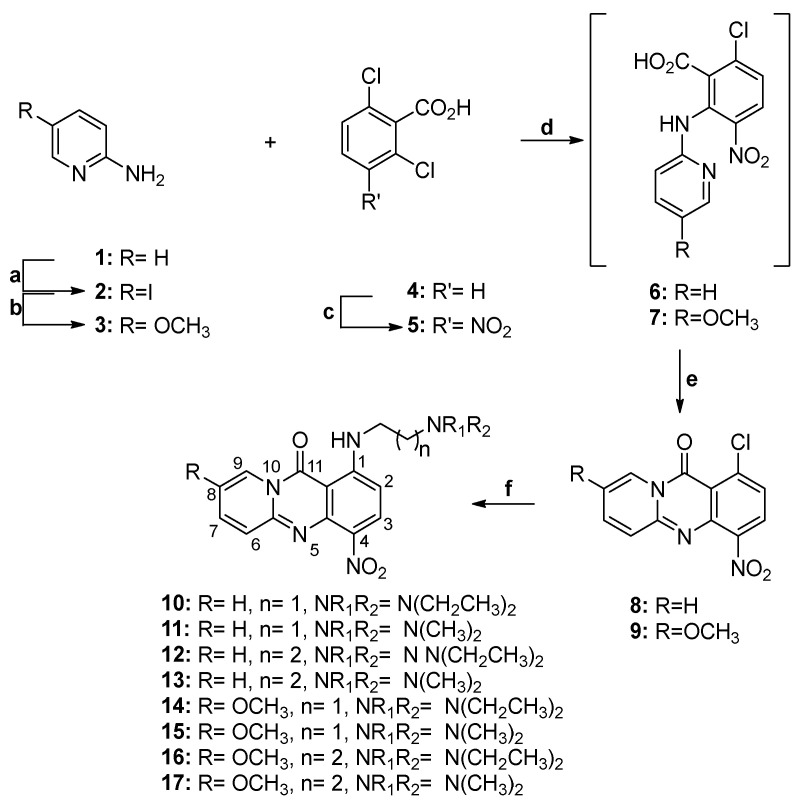
Reagents and conditions: (a) KIO_4_, I_2_, glacial acetic acid, H_2_O, H_2_SO_4_, 80 °C, 5 h, (b) Cs_2_CO_3_, CuI, 1,10-phenanthroline, MeOH, 120 °C, 12 h, (c) fuming HNO_3_, H_2_SO_4_, r.t, 1 h, (d) NaH 60% in mineral oil, dry DMF, 50 °C, 24 h, (e) PPA, 100 °C, 2 h, (f) suitable amine, THF, reflux, 12 h.

The synthesis of amino-substituted aza-acridones **12**–**17** started from 1-chloro-4-nitro-11*H*-pyrido[2,1-*b*]quinazolin-11-one (**8**) and 1-chloro-8-methoxy-4-nitro-11*H*-pyrido[2,1-*b*]quinazolin-11-one (**9**) (Scheme 2). To synthesize the nitro-substituted aza-acridone **8**, we utilized nitro derivative **5**, prepared from commercially available 2,6-dichlorobenzoic acid (**4**) following a previously described procedure [29]. Compound **5** was then reacted with 2-aminopyridine (**1**) in the presence of NaOH, yielding intermediate acid **6**, which, upon intramolecular cyclization, provided chloro-substituted aza-acridone **8** with an overall yield of 85%. For the preparation of methoxy-substituted analog **9**, we employed the same synthetic procedure, starting from 5-methoxypyridin-2-amine (**3**) with a 91% overall yield. This involved the initial preparation of pyridine **3**. Thus, commercially available 2-aminopyridine (**1**) was first reacted with iodine in the presence of KIO_4_ to afford the corresponding iodopyridine **2**. Subsequent reaction with methanol in the presence of cuprous iodide and 1,10-phenanthroline provided methoxy-substituted pyridine **3**. Finally, the reaction of these chloro-substituted acridones with the appropriate *N*,*N*-dialkylethylenediamines or *N*,*N*-dialkylpropylenediamines resulted in the target amines **10**–**17**. The yield of nucleophilic substitution ranged from 50% to 90%, depending on the amine used.

**Scheme 3:**
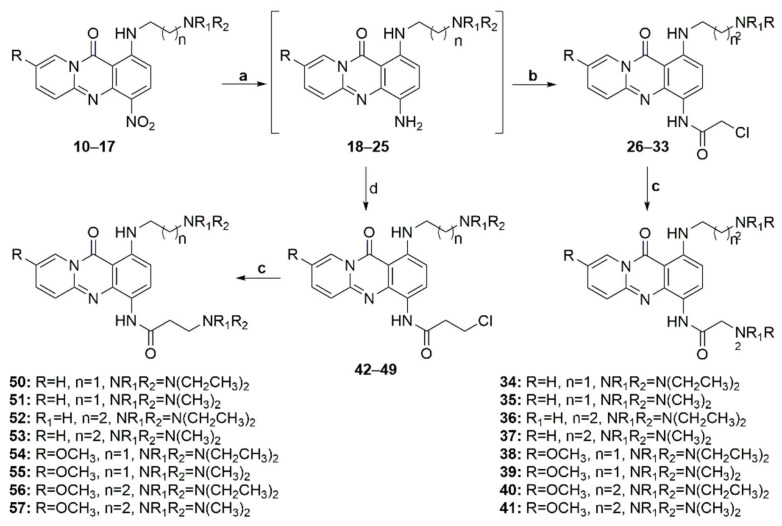
Reagents and conditions: (a) HCOONH_4_, Pd/C 10%, MeOH, 70 °C, 150 W, 2 min **18**–**21** or HCOONH_4_, Zn, MeOH, r.t., 60 min **22**–**25**, (b) ClCOCH_2_Cl, Na_2_CO_3_, THF, r.t., 15 min, (d) ClCOCH_2_CH_2_Cl, Na_2_CO_3_, THF, r.t., 15 min, (c) HNR_1_R_2_, EtOH, reflux 48–72 h.

Subsequently, the nitro groups of compounds **10**–**17** were reduced through hydrogenation over palladium on activated carbon to afford anilines **18**–**25**, which, upon treatment with chloracetyl chloride or 3-chloropropionyl chloride, afforded amides **26**–**33** and **42**–**49**, respectively (Scheme 3). Finally, the reaction of these amides with the appropriate amines yielded target diamines **34**–**41** and **50**–**57**. The overall yield for the preparation of the diamino-substituted analogs ranged from 10% to 50%, with the reduction step being the most crucial due to the instability of the intermediate aniline.

### 2.2. Biology—Pharmacology

In Vitro Cytotoxic Activity—Cell Viability Assay

All newly synthesized compounds were evaluated for their antiproliferative activity against two human cancer cell lines of high clinical relevance: T24 (urinary/urothelial bladder cancer) [30,31,32,33] and MW266-4 (skin cancer: melanoma) [34,35,36,37]. Both T24 and WM266-4 cancer cell types are known for their high malignancy grades and strong metastatic potential. The T24 (malignancy grade III) cell line is characterized by the H-RAS^G12V^ (gain of function) and the p53^ΔY126^ (loss of function) mutant protein forms (https://depmap.org/portal/cell_line/ACH-000018?tab=mutations, accessed on 20 May 2025), while the WM266-4 (metastatic) cell line is typified by the B-RAFV600D (gain of function) driver mutation (https://depmap.org/portal/cell_line/ACH-001239?tab=mutations, accessed on 20 May 2025). All three mutations are oncogenic; thus, the T24 and WM266-4 cell systems are capable of reliably recapitulating bladder cancer and metastatic melanoma malignancies, respectively.

**Figure 2:**
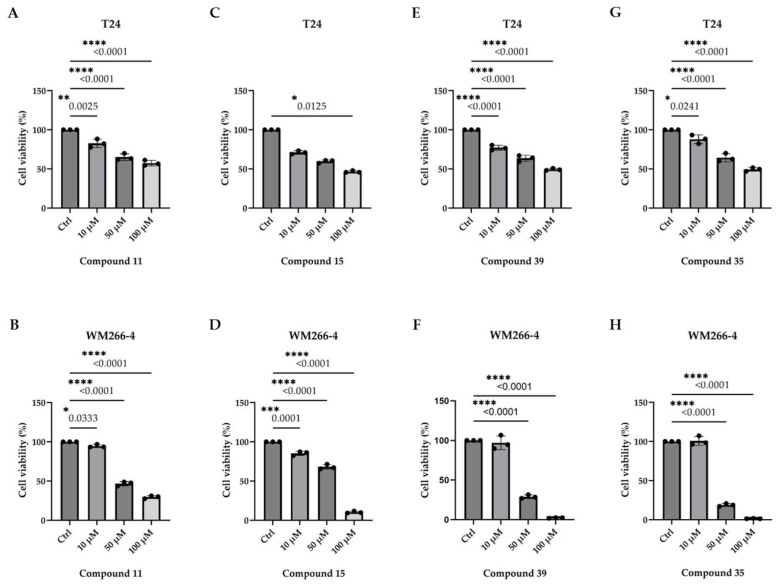
Metastatic melanoma, but not bladder cancer, cells undergo compound-specific annihilation of their survival and growth in a dose-dependent manner. MTT-based cytotoxicity assays of T24 (left-column panels) and WM266-4 (right-column panels) cells, grown at ~90% confluency under standard conditions and treated with the indicated doses (0, 10, 50, and 100 μM) of compounds **11** (top panels), **15** (second from the top panels), **39** (second from the bottom panels), and **35** (bottom panels) (all dissolved in high-grade DMSO), for 24 h. Note the striking anticancer activity (cell viability annihilation) of compound **35**, at the 100 μM concentration, against WM266-4 (human metastatic melanoma) but not against T24 (human urothelial bladder cancer) cells. Results are reported as mean ± standard deviation of triplicates of at least three independent experiments (*p* < 0.001). Statistical analysis: Statistical analyses were performed using the GraphPad Prism 9 software. Normality and lognormality of the data were assessed using the Shapiro-Wilk test, while heteroscedasticity was evaluated using the Brown-Forsythe test. Depending on the distribution, the results were analyzed using either a one-way ANOVA with Tukey’s multiple comparisons test or the Kruskal-Wallis test. Data are presented as mean ± standard deviation (SD), with n = 3. A *p*-value of less than 0.05 was considered statistically significant.

Most of the tested compounds did not significantly affect the cell viability. However, four compounds (11, 15, 35, and 39) exhibited cytotoxic effects with varying potency and selectivity. Compounds **11** and **15** exhibited modest, concentration-dependent activity in both cell lines, whereas compounds **35** and **39** were markedly more potent against WM266-4 cells. Notably, compound **35** nearly abolished WM266-4 viability at 100 μM (Figure 2), suggesting its selective activity against metastatic melanoma.

Based on the IC_50_ values in WM266-4 cells, compound **35** was the most active (24.74 ± 3.00 μM), followed by **39** (27.31 ± 3.00 μM), **15** (48.94 ± 3.00 μM), and **11** (49.63 ± 3.00 μM) (Figure S1). Interestingly, compound **35** exhibited minimal activity in T24 cells, suggesting selective cytotoxicity against melanoma. This difference may reflect the distinct mutational context of the two cell lines. In T24 cells, the coexistence of H-RAS^G12V^ and p53^ΔY126^, along with other alterations such as CAD^Q646Ter^, V^1357F^, HLA-DQB1^P84E^, SRPK2^D65A^, and WIF1^A149E^, did not sensitize them to compound **35**. In contrast, WM266-4 cells carry additional mutations beyond B-RAF^V600D^—including APC^T2178NfsTer2^, CDH2^55TfsTer23^, FGFR2^P748RfsTer20^, FGFR3^P402L^, HGF^R197C^, HLA-DQB1^Y62S^, LRP6^T83NfsTer2^, MAP3K15^W1112Ter/P730RfsTer33^, MMP9^G100E^, PRKCI^T410I^, SMAD4^P313A^, and TP73^H96PfsTer33^—which may influence their response to cytotoxic agents (https://depmap.org/portal/cell_line/ACH-001239?tab=mutations, accessed on 20 May 2025) [38].

Overall, compound **35**—and, to a lesser extent, compound **39**— emerged as promising leads for the targeted treatment of metastatic melanoma. The selective activity observed in WM266-4 cells warrants further investigation to elucidate its mechanism of action and therapeutic potential.

### 2.3. Hydrolysis Study

The subjective hydrolysis procedure was used to experimentally ascertain the stability of the investigated compounds. Hydrolysis was not performed under acidic or alkaline conditions to simulate the cellular and extracellular pH of 7.4, whereas under the same notion, the temperature was set to 23(±1) °C. A UV-Vis methodology was developed and validated to assess the quality of the procedure, including linearity, sensitivity, precision, and robustness. The λ_max_ wavelengths obtained for compound **17** were 220 nm, 342 nm, and 446 nm, while the latter one was selected as the optimum one, whereas for compound **16** were 238 nm, 373 nm, and 462 nm, and the 462 nm was selected as the optimum one (Figures S1 and S2). To ensure high selectivity, the longest wavelengths were employed for quantitative analyses. Thus, 446 and 462 nm were used for the abovementioned compounds, respectively.

The analytical methodology validation results showed excellent linearity (Table S1). The linear range of compound **16** was between 5.0 and 15.00 mg L^−1^, whereas that of compound **17** was between 5.0 and 12.5 mg L^−1^. The calibration curve equations obtained, as well as the coefficient of determination for both compounds, are listed in Table S1 in the Supplementary Material. Additionally, the calibration curves passed the normality (Shapiro-Wilk and Kolmogorov-Smirnov) and homoscedasticity tests, proving the validity of the analysis. Furthermore, no outlier data were obtained.

The repeatability of compound **16** was examined at seven levels (5 mg L^−1^, 6.25 mg L^−1^, 7.5 mg L^−1^, 8.75 mg L^−1^, 10 mg L^−1^, 12.5 mg L^−1^, and 15 mg L^−1^) with five replicate samples for each level. The mean absorbance, standard deviation (SD), and percentage of relative standard deviation (% RSD) were calculated. For all the investigated levels, the % RSD was less than 4.5%, thus demonstrating excellent repeatability. Furthermore, the repeatability of compound **17** was tested at six levels (5 mg L^−1^, 6.25 mg L^−1^, 7.5 mg L^−1^, 8.75 mg L^−1^, 10 mg L^−1^, and 12.5 mg L^−1^) with five replicate samples for each level, and the corresponding values were calculated accordingly. For all levels, the %RSD was less than 0.44%, indicating excellent repeatability. The values obtained for each level for both compounds are tabulated in Table S2 (compound **16**) and Table S3 (compound **17**) in the Supplementary Material.

The sensitivity, according to the IUPAC definition, was calculated from the calibration equation and was found to be LOD = 0.51 mg L^−1^ and LOQ= 1.54 mg L^−1^ for compound **17**. The corresponding values for compound **16** were determined to be LOD = 0.7 mg L^−1^ and LOQ = 2.1 mg L^−1^. The robustness study determined that deliberately altering the λmax for both compounds by 5 nm did not exceed 1%, expressed as %RSD.

The hydrolytic stability was assessed using linear and non-linear regression of the data obtained from the hydrolysis procedure. The linear model obtained in both cases, showing a near-zero slope for both cases, indicates the stability of both substances under the conditions used. It should be noted that under similar conditions, the previous series of compounds were readily degraded [26].

### 2.4. Computational Studies

**Figure 3:**
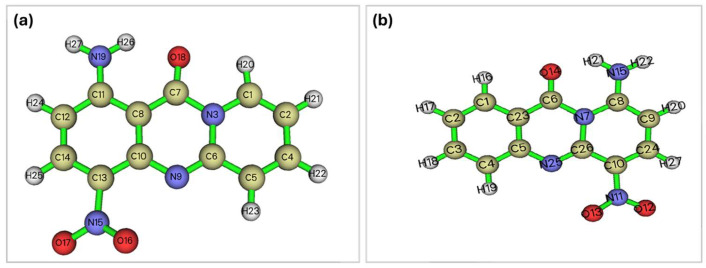
Structures of the investigated compounds: (**a**) 1A _and (**b**) 9A.

The enhanced biological activity of the new molecular scaffold was partly attributed to its hydrolytic stability. Consequently, a rigorous investigation was undertaken to assess the stability of these two scaffolds (previously named 9-amino-6-nitro-11*H*-pyrido[2,1-*b*]quinazolin-11-one and abbreviated as 9A, and the current name 1-amino-4-nitro-11*H*-pyrido[2,1-*b*]quinazolin-11-one and abbreviated as 1A) employing a quantum mechanical approach. For simplicity, the alkane groups that imposed conformational freedom on the system were removed, as they obscured the effects of the electronic configuration on the extent of hydrolysis. The main questions posed were (a) whether the C-N amide bond exhibits a significantly higher π character for 1A than for the 9A derivative, (b) whether the carbonyl carbon is more electrophilic, and (c) whether the carbonyl oxygen is more nucleophilic. Criteria (b) and (c) refer to the basic and acidic hydrolysis of amides, respectively. Furthermore, the aromaticity of the system, comprising atoms adjacent to the δ-lactam or the ring to which the lactam belongs, the bond lengths, and the planarity thereof are indicators of π-system delocalization. Multiple metrics of aromaticity have been employed, including bond lengths, their respective bond angles, and the planarity of the system, that is, the ring to which it belongs, the AIM (Atoms-In-Molecules) ring critical points, the ELF (Electron Localization Function) localization, and specialized aromaticity indices such as the HOMA (Harmonic Oscillator Model of Aromaticity), the PDI (Para-Delocalization Index) and the FLU (Aromatic Fluctuation Index) indices as well as the Shannon aromaticity index. Based on the comparison between two molecules of each group (Figure 3), it has been considered that the more delocalized the electronic density of the ring, the more stable the lactamic bond.

#### Quantum Mechanical-Based Estimation of the Lactam Ring Stability

(a) Charges

The charge trends of the two molecules were calculated to assess whether the two molecules were equally amenable to acidic or basic hydrolysis. The CHELPG ESP fitting atomic charge calculated using MultiWFN showed that the charge of the carbonyl carbon changed from 0.69 for the 1A derivative to 0.72 for the 9A derivative, indicating a more facile attack of the hydroxyl anion on the latter. Furthermore, the respective charge of the carbonyl O is −0.6 for the 1A molecule and becomes slightly higher, i.e., 0.61 for the 9A molecule, proving that the acidic hydrolysis starting with oxygen protonation is marginally facilitated in the latter derivative.

(b) Geometry analysis

The deviation of the dihedral angles for the central ring of the molecule, i.e., the lactam-bearing ring, indicates the loss of aromaticity, which in turn denotes the increased hydrolytic instability between the two derivatives.

(c) Angles and dihedral angles

The dihedral angles are listed in Table 1. The angles encompassing the lactam nitrogen moiety did not deviate appreciably from the theoretical 120° predicted for the nitrogen SP2 hybridization, except for the bond centered on the carbonyl carbon. In the latter case, the two angles are 115.99 for the 1A and 116.74 for 9A, indicating a slightly increased strain of the 1A. The calculation of the dihedral angles of the amide moiety exhibits a larger deviation from planarity for the 9A derivative, which is attributed to the higher sp3 character of the carbonyl carbon and, therefore, a slightly higher partial δ^+^ charge. Thus, the 9A carbonyl carbon is amenable to a higher hydrolysis rate due to the more facile nucleophilic attack of OH^−^ during the first step of alkaline hydrolysis.

(d) Bond lengths

The bond lengths of the two molecules are listed in Table 2. The major divergences in the bond lengths are in ring C (three bonds) and ring 1 (one bond), which can be attributed to the positioning and resulting electronic density redistribution due to the NO_2_ group. Interestingly, all the bonds of the lactamic N are contracted to 1A, showing that the aromaticity of the central ring (ring A) is enhanced.

**Figure 4:**
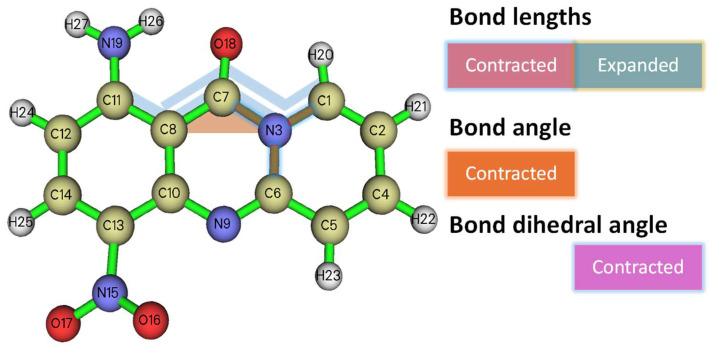
Structure of the 1A analog along ( numbering is arbitrary). The highlighted red bonds are those that are subtracted from the 1A derivative compared to 9A, whereas the blue-colored bond is elongated. The angle is subtracted, whereas the dihedral angles are contracted toward planarity.

The results of the comparison of the bond lengths, angles, and dihedral angles are shown in Figure 4.

(e) FUKUI functions

To examine the potential differences in the hydrolytic reactivity between the two molecules, the f− and f+ FUKUI functions were examined. The electrophilic and nucleophilic susceptibilities of the carbonyl carbon and its oxygen counterpart were examined to assess the differences in the basic and acidic hydrolysis of the two substances. The f-value for the carbonyl O atom is calculated to be 0.018 in 1A, whereas the corresponding value is 0.0565 for 9A, indicating that the latter can be protonated more easily. Accordingly, the f+ value of the carbonyl C is found to be 0.0469 for the 1A analog, whereas the corresponding value is calculated to be 0.0628 for its 9A counterpart, showing enhanced nucleophilic susceptibility for the latter molecule upon attack by the OH^−^ anion.

Aromaticity indices

To study whether the resistance of the 1A analog to hydrolysis is the result of the central ring-enhanced electronic charge delocalization, i.e., enhanced aromaticity, a series of aromatic indices were employed. The results are presented in Table 3.

It is apparent that upon relocation of the NO_2_ group to ring B, ring A acquires an enhanced aromatic character for derivative 1A. All the aromaticity indices show that the property is enhanced for rings A and C and reduced for ring B. This is probably the result of enhanced electron delocalization to a more extended conjugated π system, which includes the lactamic moiety. In the case of the 9A analog, NO_2_, due to its electron-drawing properties, relocated the electronic cloud toward it, causing charge separation and drawing the electronic density from the respective bonds. The only index that shows no substantial difference between the two molecules is the Shannon entropy index.

Topological analysis of the electron density

**Figure 5:**
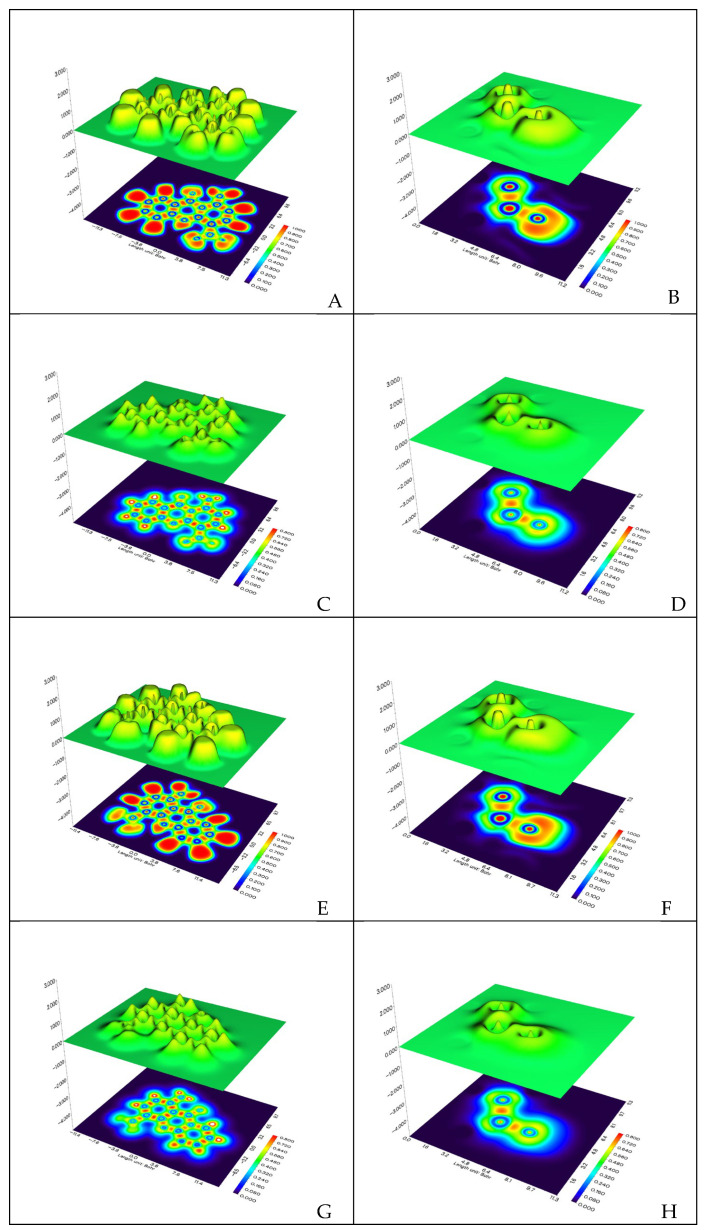
The ELF (Electron Localization Function) and LOL (Localized Orbital Locator) functions of the 1A and 9A molecules. Panels (**A**,**B**) correspond to the ELF function of the 1A derivative, and panels (**E**) correspond to those of the 9A derivative. Panels (**C**,**D**) correspond to the LOL function of the 1A derivative, and panels (**G**,**H**) correspond to the function of the 9A derivative. Panels (**B**,**D**,**F**,**H**) focus only on the three atoms of the amide bond.

Further topological analysis of the carbonyl carbon, the corresponding oxygen, and their connecting bonds was carried out using the ELF and LOL (Localized Orbital Locator) indices. The results are presented in Table S4 and Figure 5, and the CP (Critical Point) numbering is shown in Figure S4.

The ELF shows that there is a negligible difference in the CPs located on the carbonyl oxygen, carbonyl carbon, and lactamic N compared to the CPs of the two bonds, the C=O and the C-N, where the ELF values are enhanced. The only appreciable difference is located at the C=O CP, showing a slight difference, with the 1A molecule bearing slightly more σ character to this bond.

The same trend as that observed for the ELF values holds for the Localized Orbital Locator quantum chemical counterpart. Thus, the LOL is enhanced for the CPs of C=O, showing a slightly more localized electron density. In both cases, these differences could not demonstrate the increased stability of the 1A molecule.

## 3. Materials and Methods

### 3.1. General Information

All commercially available reagents and solvents were purchased from Alfa Aesar (Ward Hill, MA, USA) and used without any further purification. Melting points were determined using a Büchi apparatus and were uncorrected. All NMR spectra were recorded on 400 or 600 MHz Bruker spectrometers (Avance™ DRX and III instruments, Bruker BioSpin GmbH, Rheinstetten, Germany). ^1^H NMR (200, 400, and 600 MHz) and ^13^C NMR (50 and 151 MHz, recorded with complete proton decoupling) spectra were obtained with samples dissolved in CDCl_3_ or DMSO-*d6*, with the residual solvent signals used as internal references: 7.26 ppm for CHCl_3_ and 2.50 ppm for (CD_3_)(CD_2_H)S(O) regarding ^1^H NMR experiments; 77.2 ppm for CDCl_3_ and 39.4 ppm for (CD_3_)_2_S(O) concerning ^13^C NMR experiments. Chemical shifts (δ) are given in ppm to the nearest 0.01 (^1^H) or 0.1 ppm (^13^C) unit. The coupling constants (*J*) are expressed in hertz (Hz). The signals are reported as follows: (s = singlet, d = doublet, t = triplet, m = multiplet, and br = broad). The assignments of ^1^H and ^13^C NMR signals were unambiguously achieved with the help of D/H exchange and 2D techniques: COSY, NOESY, HMQC, and HMBC experiments. Systematic indole and pyrazole nomenclatures were used for the assignment of each spectrum. Flash chromatography was performed on Merck silica gel (40–63 μm) with the indicated solvent system using gradients of increasing polarity in most cases (Merck KGaA, Darmstadt, Germany). The reactions were monitored by analytical thin-layer chromatography (Merck pre-coated silica gel 60 F254 TLC plates, 0.25-mm layer thickness). The compounds were visualized on TLC plates using UV radiation (254 and 365 nm). All solvents used in the absorption and fluorescence experiments were of spectroscopic grade. Mass spectra were recorded on a UPLC Triple TOF-MS {UPLC: Acquity of Waters, SCIEX Triple TOF-MS 5600+ (Framingham, MA, USA)}.

### 3.2. Synthesis of Compounds **2**–**57**

**2-Amino-5-iodopyridine (2)**

To a suspension of 2-aminopyridine (4 g, 42.55 mmol, **1**), ΚΙO_4_ (1.43 g, 62.17 mmol), and Ι_2_ (4.48 g, 84.56 mmol) in a mixture of acetic acid (99.6 mL) and water (5 mL) at 0 °C, sulfuric acid 98% (1.16 mL) was added dropwise, and the resulting mixture was stirred at 80 °C for 5 h. After completion of the reaction, the mixture was poured into ice-water and saponified by the addition of 20% ΝaOH solution (pH~10). The resulting precipitate was filtered off, washed with aqueous Νa_2_S_2_O_3_ solution, and dried under vacuum over P_2_O_5_ to afford 8 g (67%) of the title compound **2**, which was practically pure and used for the next step without further purification. Mp 126–130 °C (EtOAc) [29]. ^1^HNMR (400 MHz, DMSO-*d6*) δ (ppm) 8.03 (d, *J* = 2.1 Hz, 1H, H-6), 7.58 (dd, *J* = 8.7, 2.2 Hz, 1H, H-4), 6.34 (d, *J* = 8.7 Hz, 1H, H-3), 6.11 (brs, 2H, D_2_Oexch., N*H*_2_). ^13^C NMR (151 MHz, DMSO-*d6*) δ (ppm) 157.4, 153.8, 145.3, 110.9, 77.8.

**2-Amino-5-methoxy-pyridine (3)**

A mixture of 2-amino-5-iodopyridine (6 g, 27.3 mmol, **2**), Cs_2_CO_3_ (17.8 g, 54.6 mmol), CuI (520 mg, 2.73 mmol), and 1,10-phenanthroline (0.98 g, 5.46 mmol) in anhydrous MeOH (50 mL) was stirred at 120 °C in an autoclave apparatus for 12 h. The reaction mixture was then filtered, the filter cake was washed with EtOAc, the filtrate was washed with water, dried (anhydrous Na_2_SO_4_), and evaporated to dryness. Flash chromatography on silica gel using a mixture of cyclohexane/EtOAc 3:1 as the eluent afforded the title compound (1.5 g, 44%) as a brownish oil [29]. ^1^HNMR (400 MHz, CDCl_3_) δ (ppm) 7.78 (d, *J* = 3.2 Hz, 1H, H-6), 7.09 (dd, *J* = 8.8, 3.2 Hz, 1H, H-4), 6.48 (d, *J* = 9.2 Hz, 1H, H-3), 5.43 (brs, 2H, D_2_O exch., N*H*_2_), 3.68 (s, 3H, OC*H*_3_). ^13^C NMR (101 MHz, CDCl_3_) δ (ppm) 152.9, 149.6, 133.2, 125.7, 109.4, 56.3 (O*C*H_3_).

**2,6-Dichloro-3-nitrobenzoic acid (5)**

To a suspension of 2,6-dichlorobenzoic acid (2.00 g, 10.53 mmol) in c. H_2_SO_4_ (30 mL) was added dropwise to fuming HNO3 (0.6 mL, 14.28 mmol) at 0 °C, and the mixture was stirred at room temperature for 45 min. After completion of the reaction, the mixture was poured into ice, and the resulting solid was filtered, washed with cold water, and dried over P_2_O_5_ to afford 2.4 g (97%) of the title compound **5**, which was used in the next step without any further purification. M.p.: 140–142 °C (H_2_O) [29]. ^1^HNMR (400 MHz, CDCl_3_) δ (ppm) 7.92 (d, *J* = 8.8 Hz, 1H, H-4), 7.55 (d, *J* = 8.8, 1H, H-5), 6.84 (brs, 1H, O*H*). ^13^C NMR (101 MHz, CDCl_3_) δ (ppm) 166.6, 146.6, 135.9, 135.4, 128.9, 126.9, 125.5.

**1-Chloro-4-nitro-11*H*-pyrido[2,1-*b*]quinazolin-11-one (8)**

To a solution of 2-aminopyridine (600 mg, 6.38 mmol, **1**) in anhydrous DMF (40 mL) at 0 °C under an argon atmosphere, ΝaH (60% in mineral oil) (2.5 g, 62.5 mmol) was added in portions, and the resulting mixture was stirred for 30 min at room temperature. 2-Aminopyridine (1.5 g, 6.38 mmol) was added portion-wise at 0 °C, and the resulting suspension was stirred at 50 °C for 24 h. After completion of the reaction, the mixture was allowed to cool to room temperature, poured into ice-water, and acidified by the addition of 18% HCl solution (pH~3). The resulting precipitate was filtered off and dried under vacuum over P_2_O_5_ to afford 1.59 g (85%) of the title compound **8**, which was practically pure and used for the next step without further purification. M.P.: 242–244 °C (EtOH). ^1^H NMR (600 MHz, DMSO-*d*6) δ (ppm): 8.84 (d, *J* = 7.1 Hz, 1H, H-9), 8.26 (d, *J* = 8.3 Hz, 1H, H-3), 7.88 (td, *J* = 8.4, 1.6 Hz, 1H, H-7), 7.58 (d, *J* = 8.4 Hz, 1H, H-2), 7.51 (d, *J* = 9.0 Hz, 1H, H-6), 7.20 (td, *J* = 8.4, 1.6 Hz, 1H, H-8). ^13^C NMR (151 MHz, DMSO-*d*6) δ (ppm): 155.5 (CO), 149.2 (C-5a), 145.0 (C-4a), 142.2 (C-1), 138.2 (C-7), 136.6 (C-3), 127.6 (C-3), 127.2 (C-9), 125.6 (C-6), 125.0 (C-2), 114.6 (C-11a), 114.0 (C-9).

**8-Methoxy-4-nitro-1-chloro-11*H*-pyrido[2,1-*b*]quinazolin-11-one (9)**

This compound was synthesized using a procedure analogous to that described for the preparation of compound **8**, using compound **3**. Yield: 91%. M.p.: 248–250 °C (EtOAc). ^1^H NMR (600 MHz, DMSO-d_6_) δ (ppm) 8.34 (d, *J* = 2.5 Hz, 1H, H-9), 8.27 (d, *J* = 8.3 Hz, 1H, H-3), 7.77 (dd, *J* = 7.07, 2.65 Hz, 1H, H-7), 7.61 (d, *J* = 8.3 Hz, 1H, H-2), 7.56 (d, *J* = 9.7 Hz, 1H, H-6), 3.93 (s, 3H, OC*H*_3_). ^13^C NMR (151 MHz, DMSO-d_6_) δ (ppm) 155.0 (*C*O), 149.3 (C-8), 146.6 (C-5a), 145.0 (C-4a), 141.6 (C-1), 136.4 (C-4), 133.6 (C-7), 127.3 (C-3), 126.9 (C-6), 125.2 (C-2), 113.2 (C-11a), 106.2 (C-9), 56.4 (OC*H*_3_).

**1-((2-(Diethylamino)ethyl)amino)-4-nitro-11*H*-pyrido[2,1-*b*]quinazolin-11-one (10)**

A solution of **8** (1 g, 3.64 mmol) and *N*,*N*-diethylethylenediamine (1 mL, 7.13 mmol) in THF (40 mL) was refluxed for 24 h. Upon cooling, the mixture was vacuum-evaporated and extracted with CH_2_Cl_2_ -water; the organic layer was dried (using Na_2_SO_4_) and evaporated to dryness. The residue was purified by column chromatography (silica gel, CH_2_Cl_2_-MeOH 10:0–9:1) to afford the title compound as a yellow solid (870 mg, 67% yield). M.p.: 123–125 °C (EtOAc). ^1^H NMR (600 MHz, CDCl_3_) δ (ppm): 9.87 ((brs, 1H, D_2_O exch., N*H*), 8.82 (d, *J* = 7.2 Hz, 1H, H-9), 8.32 (d, *J* = 9.3 Hz, 1H, H-3), 7.62 (t, *J* = 6.61 Hz, 1H, H-7), 7.55 (d, *J* = 9.0 Hz, 1H, H-6), 6.94 (t, *J* = 6.27 Hz, 1H, H-8), 6.34 (d, *J* = 9.3 Hz, 1H, H-2), 3.41 (q, *J* = 6.3 Hz, 2H, NHC*H_2_*CH_2_), 2.83 (t, *J* = 6.3 Hz, 2H, NHCH_2_C*H_2_*), 2.66 (q, *J* = 7.1 Hz, 4H, N(C*H_2_*CH*_3_*)_2_), 1.09 (t, *J* = 7.1 Hz, 6H, N(CH_2_C*H_3_*)_2_). ^13^C NMR (151 MHz, CDCl_3_) δ (ppm): 159.4 (CO), 154.9 (C-1), 148.7 (C-5a), 145.5 (C-4a), 136.2 (C-7), 134.6 (C-3), 131.1 (C-4), 126.7 (C-6), 126.4 (C-9), 113.6 (C-8), 101.8 (C-2), 100.7 (C-11a), 50.7 (NHCH*_2_C*H*_2_*), 46.9 (NH(*C*H*_2_*CH*_3_*)_2_), 41.3 (NH*C*H*_2_*CH*_2_*), 11.8 (NH(CH*_2_C*H*_3_*)_2_). *m*/*z*: calcd. for C_18_H_22_N_5_O_3_^+^: [M1 + H]^+^ = 356.1717, found 356.1724.

**1-((2-(Dimethylamino)ethyl)amino)-4-nitro-11*H*-pyrido[2,1-*b*]quinazolin-11-one (11)**

This compound was synthesized using a procedure analogous to that described for the preparation of **10**, using compound **8** and *N,N*-dimethylethylenediamine. Yield: 76%. M.p.: 160–162 °C (EtOAc). ^1^H NMR (600 MHz, CDCl_3_) δ (ppm): 9.85 (brs, 1H, D_2_O exch., N*H*), 8.83 (d, *J* = 7.3 Hz, 1H, H-9), 8.33 (d, *J* = 9.3 Hz, 1H, H-3), 7.58–7.63 (m, 2H, H-6, H-7), 6.91 (t, *J* = 7.4 Hz, 1H, H-8), 6.34 (d, *J* = 9.3 Hz, 1H, H-2), 3.4 (q, *J* = 6.1 Hz, 2H, NHC*H_2_*CH_2_), 2.67 (t, *J* = 6.2 Hz, 2H, NHCH_2_C*H_2_*), 2.34 (s, 6H, N(C*H_3_*)_2_). ^13^C NMR (151 MHz, CDCl_3_) δ (ppm): 159.8 (CO), 155.1 (C-1), 148.9 (C-5a), 145.7 (C-4a), 136.2 (C-7), 134.9 (C-3), 131.6 (C-4), 127.0 (C-6), 126.6 (C-9), 113.7 (C-8), 101.8 (C-2), 101.0 (C-11a), 57.5 (NHCH_2_*C*H_2_), 45.6 (N(*C*H*_3_*)_2_), 41.2 (NH*C*H_2_CH_2_). *m*/*z*: calcd. for C_16_H_18_N_5_O_3_^+^: [M1 + H]^+^ = 328.1404, found 328.1411.

**1-((3-(Diethylamino)propyl)amino)-4-nitro-11*H*-pyrido[2,1-*b*]quinazolin-11-one (12)**

This compound was synthesized using a procedure analogous to that described for the preparation of **10**, using compound **8** and *N,N*-diethyl-1,3-propanediamine. Yield: 56%. M.p.: 217–219 °C (EtOAc). ^1^H NMR (600 MHz, CDCl_3_) δ (ppm): 9.76 (brs, 1H, D_2_O exch., N*H*), 8.80 (d, *J* = 7.2 Hz, 1H, H-9), 8.34 (d, *J* = 9.3 Hz, 1H, H-3), 7.58–7.63 (m, 2H, H-6, H-7), 6.95 (t, *J* = 6.7 Hz, 1H, H-8), 6.40 (d, *J* = 9.3 Hz, 1H, H-2), 3.43 (q, *J* = 6.2 Hz, 2H, NHC*H_2_*CH_2_CH_2_), 2.61–2.63 (m, 6H, NHCH_2_CH_2_C*H_2_*, N(C*H*_2_*C*H*_3_*)_2_), 1.94 (quintet, *J* = 6.9 Hz, 2H, NHCH_2_C*H_2_*CH_2_), 1.06 (t, *J* = 7.2 Hz, 6H, N(CH_2_*CH*_3_)_2_). ^13^C NMR (151 MHz, CDCl_3_) δ (ppm): 159.9 (CO), 155.3 (C-1), 148.8 (C-5a), 145.6 (C-4a), 136.3 (C-7), 134.9 (C-3), 131.4 (C-4), 127.0 (C-6), 126.4 (C-9), 113.9 (C-8), 101.8 (C-2), 100.8 (C-11a), 50.3 (NHCH_2_CH_2_*C*H_2_ ), 47.1 (N(*C*H_2_CH_3_)_2_), 41.7 (NH*C*H_2_CH_2_CH_2_), 26.4 (NHCH_2_*C*H_2_CH_2_), 11.6 (N(CH_2_*C*H_3_)_2_). *m*/*z*: calcd. for C_19_H_24_N_5_O_3_^+^: [M1 + H]^+^ = 370.1874, found 370.1867.

**1-((3-(Dimethylamino)propyl)amino)-4-nitro-11*H*-pyrido[2,1-*b*]quinazolin-11-one (13)**

This compound was synthesized using a procedure analogous to that described for the preparation of **10,** using compound **8** and *N,N*-dimethyl-1,3-propanediamine. Yield 94%. M.p.: 134–136 °C (EtOAc). ^1^H NMR (600 MHz, CDCl_3_) δ (ppm): 9.78 (brs, 1H, D_2_O exch., N*H*), 8.82 (d, *J* = 7.3 Hz, 1H, H-9), 8.35 (d, *J* = 9.4 Hz, 1H, H-3), 7.59–7.64 (m, 2H, H-6, H-7), 6.95 (t, *J* = 8.2 Hz, 1H, H-8), 6.42 (d, *J* = 9.4 Hz, 1H, H-2), 3.44 (q, *J* = 6.2 Hz, 2H, NHC*H_2_*CH_2_CH_2_), 2.45 (t, *J* = 6.9 Hz, 2H, NHCH_2_CH_2_C*H_2_*), 2.27 (s, 6H, N(C*H_3_*)_2_) 1.93 (quintet, *J* = 6.9 Hz, 2H, NHCH_2_C*H_2_*CH_2_). ^13^C NMR (151 MHz, CDCl_3_) δ (ppm): 159.9 (CO), 155.3 (C-1), 148.8 (C-5a), 145.7 (C-4a), 136.2 (C-7), 134.9 (C-3), 131.5 (C-4), 127.1 (C-6), 126.4 (C-9), 113.8 (C-8), 101.8 (C-2), 100.9 (C-11a), 57.1 (NHCH_2_CH_2_*C*H_2_), 45.6 (N(*C*H_3_)_2_), 41.56 (NH*C*H_2_CH_2_CH_2_), 26.9 (NHCH_2_*C*H_2_CH_2_). *m*/*z*: calcd. for C_17_H_20_N_5_O_3_^+^: [M1 + H]^+^ = 342.1561, found 342.1569.

**1-((2-(Diethylamino)ethyl)amino)-8-methoxy-4-nitro-11*H*-pyrido[2,1-*b*]quinazolin-11-one (14)**

This compound was synthesized using a procedure analogous to that described for the preparation of **10**, using compound **9** and *N,N*-diethylethylenediamine. Yield: 63%. M.p.: 140–142 °C (EtoAc). ^1^H NMR (600 MHz, CDCl_3_) δ (ppm): 9.94 (brs, 1H, D2O exch., NH), 8.38 (d, *J* = 9.3 Hz, 1H, H-3), 8.32 (s, *J* = 2.67 Hz, 1H, H-9), 7.58 (d, *J* = 9.7 Hz,1H, H-6), 7.45 (dd, *J* = 9.73, 2.74 Hz 1H, H-7), 6.37 (d, *J* = 9.4 Hz, 1H, H-2), 3.93 (s, 3H, OC*H*_3_), 3.41 (q, *J* = 6.4 Hz, 2H, NHC*H_2_*CH*_2_*), 2.83 (t, *J* = 6.3 Hz, 2H, NHCH*_2_*C*H_2_*), 2.65 (q, *J* = 7.1 Hz, 4H, N(C*H_2_*CH*_3_*)_2_), 1.10 (t, *J* = 7.12 Hz, 6H, N(CH*_2_*C*H*_3_)_2_). ^13^C NMR (151 MHz, CDCl_3_) δ (ppm): 159.4 (*C*O), 155.1 (C-1), 149.7 (C-8), 146.5 (C-5a), 145.3 (C-4a), 134.7 (C-3), 132.6 (C-7), 131.3 (C-4), 127.9 (C-6), 105.5 (C-9), 101.7 (C-2), 100.8 (C-11a), 56.5 (O*C*H_3_), 51.0 (NHCH*_2_C*H*_2_*), 47.1 (N(*C*H*_2_*CH*_3_*)_2_), 41.6 (NH*C*H*_2_*CH*_2_*), 12.0 (N(CH*_2_C*H*_3_*)_2_). *m*/*z*: calcd. for C_19_H_24_N_5_O_4_^+^: [M1 + H]^+^ = 386.1823, found 386.1830.

**1-((2-(Dimethylamino)ethyl)amino)-8-methoxy-4-nitro-11*H*-pyrido[2,1-*b*]quinazolin-11-one (15)**

This compound was synthesized using a procedure analogous to that described for the preparation of **10**, using compound **9** and *N*,*N*-dimethylethylenediamine. Yield: 50%. M.p.: 193–195 °C (EtOAc). ^1^H NMR (600 MHz, CDCl_3_) δ (ppm): 9.90 (brs, 1H, D_2_O exch., N*H*CO), 8.37 (d, *J* = 9.3 Hz, 1H, H-3), 8.34 (s, 1H, H-9), 7.58 (d, *J* = 9.7 Hz, 1H, H-6), 7.44 (dd, *J* = 9.7, 2.26Hz, 1H, H-7), 6.36 (d, *J* = 9.4, Hz, 1H, H-2), 3.92 (s, 3H, OC*H*_3_), 3.42 (q, *J* = 11.0, 6.0 Hz, 2H, NHC*H_2_*CH*_2_*), 2.70 (t, *J*= 9.7 Hz, 2H, NHCH*_2_*C*H_2_*), 2.36 (s, 6H, N(C*H_3_*)_2_). ^13^C NMR (151 MHz, CDCl_3_) δ (ppm): 159.5 (*C*O), 155.0 (C-1), 149.7 (C-8), 146.5 (C-5a), 145.2 (C-4a), 134.7 (C-3), 132.7 (C-7), 131.4 (C-4), 127.9 (C-6), 105.5 (C-9), 101.6 (C-2), 100.8 (C-11a), 57.6 (NHCH*_2_C*H_2_), 56.5 (O*C*H_3_), 45.6 (N(C*H_3_*)_2_), 41.2 (NH*C*H*_2_*CH*_2_*). *m*/*z*: calcd. for C_17_H_20_N_5_O_4_^+^: [M1 + H]^+^ = 358.1510, found 358.1517.

**1-((3-(Diethylamino)propyl)amino)-8-methoxy-4-nitro-11*H*-pyrido[2,1-*b*]quinazolin-11-one (16)**

This compound was synthesized using a procedure analogous to that described for the preparation of **10**, using compound **9** and *N*,*N*-diethyl-1,3-propanediamine. Yield: 53%. M.p.: 230 °C (dec). ^1^H NMR (600 MHz, CDCl_3_) δ (ppm) 9.94 (t, 1H, *J* = 5.0 Hz, D_2_Oexch., N*H*), 8.36 (d, *J* = 9.2 Hz, 1H, H-3), 8.27 (d, *J* = 2.6 Hz, 1H, H-9), 7.59 (d, *J* = 9.8 Hz, 1H, H-6), 7.48 (dd, *J* = 9.8, 2.7 Hz, 1H, H-7), 6.39 (d, *J* = 9.3, Hz, 1H, H-2), 3.94 (s, 3H, OC*H*_3_), 3.56 (q, *J* = 6.2 Hz, 2H, NHC*H_2_*CH*_2_*CH_2_), 2.30 (quintet, *J* = 6.8 Hz, 2H, NHCH*_2_*C*H_2_*CH_2_), 3.07–3.09 (m, 6H, N(C*H_2_*CH*_3_*)_2_, NHCH_2_CH_2_C*H*_2_), 1.73 (t, *J* = 7.2 Hz, 6H, N(CH*_2_*C*H_3_*)_2_). ^13^C NMR (50 MHz, CDCl_3_) δ (ppm) 159.5 (*C*O), 155.1 (C-1), 149.8 (C-8), 146.4 (C-5a), 145.1 (C-4a), 134.7 (C-3), 132.7 (C-7), 131.2 (C-4), 127.9 (C-6), 105.2 (C-9), 101.6 (C-2), 100.6 (C-11a), 56.4 (O*C*H_3_), 51.0 (NHCH_2_CH*_2_C*H*_2_*), 46.9 (N(*C*H*_2_*CH*_3_*)_2_), 41.5 (NH*C*H*_2_*CH_2_CH_2_), 26.2 (NHCH*_2_C*H_2_CH_2_), 11.4 (N(CH*_2_C*H*_3_*)_2_). *m*/*z*: calcd. for C_20_H_26_N_5_O_4_^+^: [M1 + H]^+^ = 400.1979, found 400.1972.

**1-((3-(Dimethylamino)propyl)amino)-8-methoxy-4-nitro-11*H*-pyrido[2,1-*b*]quinazolin-11-one (17)**

This compound was synthesized using a procedure analogous to that described for the preparation of **10**, using compound **9** and *N*,*N*-dimethyl-1,3-propanediamine. Yield: 61%. M.p.: 164–167 °C (EtOAc). ^1^H NMR (600 MHz, CDCl_3_) δ (ppm): 9.80 (brs, 1H, D_2_O exch., N*H*), 8.34 (d, *J* = 9.2 Hz, 1H, H-3), 8.27 (d, *J* = 2.66 Hz, 1H, H-9), 7.55 (d, *J* = 9.7 Hz,1H, H-6), 7.44 (dd, *J* = 9.7, 2.7 Hz, 1H, H-7), 6.39 (d,*J* = 8.9 Hz, 1H, H-2), 3.92 (s, 3H, OC*H*_3_), 3.42 (q, *J* = 6.8 Hz, 2H, NHC*H_2_*CH*_2_*CH_2_), 2.45 (t, *J* = 6.9 Hz, 2H, NHCH*_2_*CH_2_C*H*_2_), 2.27 (s, 1H, 6H, N(C*H_3_*)_2_), 1.94 (quintet, *J* = 7.0 Hz, 2H, NHCH_2_C*H_2_*CH_2_). ^13^C NMR (50 MHz, CDCl_3_) δ (ppm): 159.5 (*C*O), 155.2 (C-1), 149.8 (C-8), 146.4 (C-5a), 145.1 (C-4a), 134.7 (C-3), 132.6 (C-7), 131.2 (C-4), 127.9 (C-6), 105.2 (C-9), 101.6 (C-2), 100.7 (C-11a), 57.0 (NHCH_2_CH*_2_C*H*_2_*), 56.4 (O*C*H_3_), 45.6 (N(*C*H*_3_*)_2_), 41.5 (NH*C*H*_2_*CH_2_CH_2_), 26.9 (NHCH*_2_C*H_2_CH_2_). *m*/*z*: calcd. for C_18_H_22_N_5_O_4_^+^: [M1 + H]^+^ = 372.1666, found 372.1673.

**2-(Diethylamino)-N-(1-((2-(diethylamino)ethyl)amino)-11-oxo-11*H*-pyrido[2,1-*b*]quinazolin-4-yl)acetamide (34)**

A mixture of compound **10** (150 mg, 0.42 mmol), ammonium formate (400 mg, 6.30 mmol), and a catalytic amount of 10% palladium charcoal (45 mg) in anhydrous methanol (20 mL) was placed in a quartz tube, and the resulting suspension was heated under microwaves at 70 °C (150 W) for 2 min. The catalyst was filtered through celite, and the solvent was evaporated under a vacuum. The obtained crude compound **18** was immediately dissolved in THF (25 mL) and CH_2_Cl_2_ (5 mL) without any further purification, and Na_2_CO_3_ (1.6 g, 15.12 mmol) and chloroacetyl chloride (0.6 mL, 7.56 mmol) were added. The resulting suspension was stirred at room temperature for 12 h, and the organic solvent was evaporated under vacuum. The residue was dissolved in CH_2_Cl_2_, washed with a 10% Na_2_CO_3_ solution and water, dried over Na_2_SO_4_, and evaporated. Without further purification, the residue was dissolved in a mixture of abs. ethanol (20 mL) and CH_2_Cl_2_ (2 mL), diethylamine (3.5 mL, 33.8 mmol) was added. The resulting solution was then refluxed for 72 h. Upon cooling, the mixture was vacuum-evaporated and extracted with CH_2_Cl_2_-water. The organic layer was dried (using Na_2_SO_4_) and evaporated to dryness. The residue was purified by column chromatography (silica gel, CH_2_Cl_2_-MeOH 97:3–90:10) to afford the title compound as an orange solid (80 mg, 74%). M.p.: 90–92 °C (EtOAc). ^1^H NMR (400 MHz, CDCl_3_) δ (ppm): 10.81 (brs, 1H, D_2_O exch., N*H*CO), 8.80 (d, *J* = 8.9 Hz, 1H, H-3), 8.76 (d, *J* = 7.4 Hz, 1H, H-9), 8.49 (brs, 1H, D_2_O exch., N*H*CH_2_CH_2_,) 7.40 (t, *J* = 7.8 Hz, 1H, H-7), 7.34 (d, *J* = 9.0 Hz, 1H, H-6), 6.77 (t, *J* = 6.7 Hz, 1H, H-8), 6.47 (d, *J* = 8.9 Hz, 1H, H-2), 3.50 (brs, 2H, NHC*H*_2_CH_2_), 3.24 (s, 2H, COC*H*_2_), 2.98 (t, *J* = 6.7 Hz, 2H, NHCH_2_C*H*_2_), 2.85 (q, *J* = 7.1 Hz, 4H, CH_2_CH_2_N(C*H*_2_CH_3_)_2_), 2.70 (q, *J* = 7.1 Hz, 4H, COCH_2_N(C*H*_2_CH_3_)_2_), 1.20–1.15 (m, 12H, CH_2_CH_2_N(CH_2_C*H*_3_)_2_, COCH_2_N(CH_2_C*H*_3_)_2_). ^13^C NMR (50 MHz, CDCl_3_) δ (ppm): 169.7 (NHCO), 160.0 (CO), 146.7 (C-4a, C-5a), 139.6 (C-1), 134.2 (C-7), 126.5 (C-9), 126.1 (C-3), 126.0 (C-6), 121.0 (C-4), 112.0 (C-8), 103.0 (C-2), 101.7 (C-11a), 59.2 (CO*C*H_2_), 51.3 (CH_2_*C*H_2_N(CH_2_CH_3_)_2_), 48.6 (COCH_2_N(*C*H_2_CH_3_)_2_), 47.2 (CH_2_CH_2_N(*C*H_2_CH_3_)_2_), 41.2 (*C*H_2_CH_2_N(CH_2_CH_3_)_2_), 12.9 (COCH_2_N(CH_2_*C*H_3_)_2_), 11.7 (CH_2_CH_2_N(CH_2_*C*H_3_)_2_). *m*/*z*: calcd. for C_24_H_35_N_6_O_2_^+^: [M1 + H]^+^ = 439.2816, found 439.2811.

**2-(Dimethylamino)-N-(1-((2-(dimethylamino)ethyl)amino)-11-oxo-11*H*-pyrido[2,1-*b*]quinazolin-4-yl)acetamide (35)**

This compound was synthesized using a procedure analogous to that described for the preparation of **34**, using compound **11** and dimethylamine. Yield: 47%. M.p.: 230 °C (dec) (EtOAc). ^1^H NMR (400 MHz, CDCl_3_) δ (ppm): 10.40 (brs, 1H, D_2_O exch., N*H*CO), 8.73 (d, *J* = 7.2 Hz, 1H, H-3), 8.68 (d, *J* = 8.8 Hz, 1H, H-9), 8.45 (t,*J* = 4.5 Hz, 1H, D_2_O exch., N*H*CH_2_CH_2_,), 7.46 (t, *J* = 9.0 Hz, 1H, H-7), 7.35 (d, *J* = 9.0 Hz, 1H, H-6), 6.81 (t, *J* = 10.04 Hz, 1H, H-8), 6.47 (d, *J* = 8.9 Hz, 1H, H-2), 3.64 (q, *J* = 6.3 Hz, 2H, NHC*H*_2_CH_2_), 3.17 (s, 2H, COC*H*_2_), 3.02 (t, *J* = 6.3 Hz, 2H, NHCH_2_C*H*_2_), 2.63 (s, 6H, CH_2_CH_2_N(C*H_3_*)_2_), 2.45 (s, 6H, COCH_2_N(C*H_3_*)_2_). ^13^C NMR (50 MHz, CDCl_3_+1drop of MeOD) δ (ppm): 168.5 (NHCO), 160.1 (CO), 146.9 (C-5a), 145.6 (C-4a), 140.2 (C-1), 134.7 (C-7), 126.3 (C-9, C-3, C-6), 121.2 (C-4), 112.7 (C-8), 102.6 (C-2), 101.7 (C-11a), 63.6 (CO*C*H_2_), 56.4 (CH_2_*C*H_2_N(CH_3_)_2_), 46.0 (COCH_2_N(*C*H_3_)_2_), 44.0 (CH_2_CH_2_N(*C*H_3_)_2_), 39.0 *C*H_2_CH_2_N(CH_3_)_2_). *m*/*z*: calcd. for C_20_H_27_N_6_O_2_^+^: [M1 + H]^+^ = 383.2190, found 383.2199.

**2-(Diethylamino)-N-(1-((3-(diethylamino)propyl)amino)-11-oxo-11H-pyrido[2,1-b]quinazolin-4-yl)acetamide (36)**

This compound was synthesized using a procedure analogous to that described for the preparation of **34**, using compound **12** and diethylamine. Yield: 35%. M.p.: 86–88 °C (EtOAc). ^1^H NMR (400 MHz, CDCl_3_) δ (ppm): 10.70 (brs, 1H, D_2_O exch., N*H*CO), 8.67 (d, *J* = 8.9 Hz, 1H, H-3), 8.63 (d, *J* = 7.4 Hz, 1H, H-9), 8.26 (t, *J* = 4.7 Hz, 1H, D_2_O exch., N*H*CH_2_CH_2_CH_2_), 7.39 (t, *J* = 7.8 Hz, 1H, H-7), 7.23 (d, *J* = 7.7 Hz, 1H, H-6), 6.71 (t, *J* = 7.0 Hz, 1H, H-8), 6.34 (d, *J* = 10.8 Hz, 1H, H-2), 3.27 (q, *J* = 6.8 Hz, 2H, NHC*H*_2_CH_2_CH_2_), 3.16 (s, 2H, COC*H*_2_), 2.96 (t, 2H, *J* = 7.1 Hz, NHCH_2_CH_2_C*H*_2_), 2.84 (q, *J* = 7.1 Hz, 4H, CH_2_CH_2_CH_2_N(C*H*_2_CH_3_)_2_), 2.62 (q, *J* = 7.2 Hz, 4H, COCH_2_N(C*H*_2_CH_3_)_2_), 2.12 (quintet, *J* = 7.59, 2H, NHCH_2_C*H*_2_CH_2_), 1.27 (t, *J* = 7.12 Hz, 6H, CH_2_CH_2_CH_2_N(CH_2_C*H*_3_)_2_), 1.09 (t, *J* = 7.1 Hz, 6H, COCH_2_N(CH_2_C*H*_3_)_2_,). ^13^C NMR (50 MHz, CDCl_3_) δ (ppm): 170.0 (HN*C*O), 160.0 (*C*O), 146.6 (C-5a), 146.1 (C-4a), 139.7 (C-1), 134.5 (C-7), 126.1 (C-9), 125.9 (C-3), 125.7 (C-6), 121.2 (C-4), 112.4 (C-8), 102.6 (C-2), 101.6 (C-11a), 58.2 (CO*C*H_2_), 48.8 (NHCH_2_CH_2_*C*H_2_), 47.9 (COCH_2_N(*C*H_2_CH_3_)_2_), 46.2 (NHCH_2_CH_2_CH_2_N(*C*H_2_CH_3_)_2_), 39.7 (NH*C*H_2_CH_2_CH_2_), 22.6 (NHCH_2_*C*H_2_CH_2_), 12.1 (COCH_2_CH_2_N(CH_2_*C*H_3_), 8.3 (CH_2_CH_2_CH_2_N(CH_2_*C*H_3_)_2_). *m*/*z*: calcd. for C_25_H_37_N_6_O_2_^+^: [M1 + H]^+^ = 453.2973, found 453.2965.

**2-Dimethylamino-*N*-(1-((3-(dimethylamino)propyl)amino)-11-oxo-11*H*-pyrido[2,1-*b*]quinazolin-4-yl)acetamide (37)**

This compound was synthesized using a procedure analogous to that described for the preparation of **34**, using compound **13** and dimethylamine. Yield: 27%. M.p.: 98–100 °C (EtOAc). ^1^H NMR (400 MHz, CDCl_3_) δ (ppm): 10.40 (brs, 1H, D_2_O exch., N*H*CO), 8.71–8.74 (m, 2H, H-3, H-9), 8.36 (brs, 1H, D_2_O exch., N*H*CH_2_CH_2_CH_2_, D_2_Oexchange), 7.43 (t, *J* = 6.3 Hz, 1H, H-7), 7.36 (d, *J* = 9.0 Hz, 1H, H-6), 6.77 (t, *J* = 7.5 Hz, 1H, H-8), 6.46 (d, *J* = 9.0 Hz, 1H, H-2), 3.30 (q, *J* = 12.1, 6.7 Hz, 2H, NHC*H*_2_CH_2_CH_2_), 3.16 (s, 2H, COC*H*_2_) 2.43–2.47 (m, 8H, NHCH_2_CH_2_C*H*_2_, CH_2_CH_2_CH_2_N(C*H*_3_)_2_), 2.29 (s, 6H, COCH_2_N(C*H*_3_)_2_), 1.92 (quintet, *J* = 7.12 Hz, 2H, NHCH_2_C*H*_2_CH_2_). ^13^C NMR (50 MHz, CDCl_3_) δ (ppm): 168.4 (HN*C*O), 160.3 (*C*O), 147.2 (C-5a), 146.8 (C-4a), 139.9(C-1), 134.2 (C-7), 126.7 (C-9), 126.5 (C-3, C-6), 120.7 (C-4), 112.2 (C-8), 103.1 (C-2), 101.7 (C-11a), 64.4 (CO*C*H_2_), 57.3 (NHCH_2_CH_2_*C*H_2_), 46.4 (COCH_2_N(*C*H_3_)_2_), 45.7 (NHCH_2_CH_2_CH_2_N(*C*H_3_)_2_), 41.5 (NH*C*H_2_CH_2_CH_2_), 27.3 (NHCH_2_*C*H_2_CH_2_). *m*/*z*: calcd. for C_21_H_29_N_6_O_2_^+^: [M1 + H]^+^ = 397.2347, found 397.2354.

**2-Diethylamino-N-(1-((2-(diethylamino)ethyl)amino)-8-methoxy-11-oxo-11*H*-pyrido[2,1-*b*]quinazolin-4-yl)acetamide (38)**

A suspension of **14** (200 mg, 0.52 mmol), ammonium formate (50 mg, 0.78 mmol), and activated zinc (136 mg, 2.08 mmol) in anhydrous methanol (10 mL) was stirred at room temperature for 1 h. After completion of the reaction, the mixture was filtered through a Celite pad, and the filtrate was evaporated to dryness. Without further purification, the obtained crude **22** was immediately dissolved in a mixture of THF (25 mL) and CH_2_Cl_2_ (5 mL), and Na2CO3 (170 mg, 1.56 mmol) and chloroacetyl chloride (0.06 mL, 0.78 mmol) were added to this solution. The resulting suspension was stirred for 15 min at room temperature. The solvent was then evaporated under vacuum, and the residue was dissolved in CH_2_Cl_2_, washed with water, dried over Na_2_SO_4_, and evaporated to dryness to obtain the oil corresponding to compound **30**. Without further purification, the residue was dissolved in a mixture of absolute. ethanol (20 mL) and) and_2_ (2 mL), and diethylamine (3.5 mL, 33.8 mmol) was added to this solution. The resulting mixture was then refluxed for 72 h. Upon cooling, the mixture was vacuum-evaporated and extracted with CH_2_Cl_2_ -water; the organic layer was dried (anhydrous Na_2_SO_4_) and evaporated to dryness. The residue was purified by column chromatography (silica gel, CH_2_Cl_2_-MeOH 100:0–75:25) to afford the title compound as an orange solid (20 mg, 18% yield). M.p.: 144–146 °C (EtOAc). ^1^H NMR (600 MHz, CDCl_3_) δ (ppm): 10.76 (brs, 1H, D_2_O exch., N*H*CO), 8.77 (d, *J* = 8.9 Hz, 1H, H-3), 8.44 (t, *J* = 4.6 Hz, 1H, D_2_O exch., N*H*CH*_2_*CH_2_), 8.22 (d, *J* = 2.6 Hz, 1H, H-9), 7.29 (d, *J* = 9.6 Hz, 1H, H-6), 7.26 (dd, *J* = 9.75, 2.57 Hz, 1H, H-7), 6.44 (d, *J* = 8.9 Hz, 1H, H-2), 3.87 (s, 3H, OC*H*_3_), 3.405 (q, *J* = 6.2 Hz, 3H, NHC*H_2_*CH_2_), 3.22 (s, 2H, COC*H*_2_), 3.01 (t, *J* = 6.5 Hz, 2H, NHCH_2_C*H*_2_), 2.62–2.68 (m, 4H, NHCH_2_CH_2_N(C*H_2_*CH*_3_*)_2_, COCH_2_N(CH*_2_*C*H_3_*)_2_), 1.21–1.07 (m, 12H, HNCH_2_CH_2_N(CH*_2_*C*H_3_*)_2_, COCH_2_N(CH*_2_*C*H_3_*)_2_). ^13^C NMR (151 MHz, CDCl_3_) δ (ppm): 169.7 (NH*C*O), 159.8 (*C*O), 148.4 (C-8), 146.3 (C-5a), 144.3 (C-4a), 139.5 (C-1), 131.0 (C-7), 127.0 (C-6), 125.3 (C-3), 121.1 (C-4), 105.0 (C-9), 102.9 (C-2), 101.6 (C-11a), 59.2 (CO*C*H_2_), 56.3 (O*C*H_3_), 51.4 (NHCH*_2_C*H*_2_*), 48.7 (COCH_2_N(*C*H*_2_*CH*_3_*)_2_), 47.3 (NHCH_2_CH_2_N(*C*H*_2_*CH*_3_*)_2_), 41.3 (NH*C*H*_2_*CH_2_), 12.9 (COCH_2_N(CH*_2_C*H*_3_*)_2_), 11.7 (NHCH_2_CH_2_N(CH*_2_C*H*_3_*)_2_). *m*/*z*: calcd. for C_25_H_37_N_6_O_3_^+^: [M1 + H]^+^ = 469.2922, found 469.2915.

**2-Dimethylamino-*N*-(1-((2-(dimethylamino)ethyl)amino)-8-methoxy-11-oxo-11*H*-pyrido[2,1-*b*]quinazolin-4-yl)acetamide (39)**

This compound was synthesized using a procedure analogous to that described for the preparation of **38**, using compound **15** and dimethylamine. Yield: 35%. M.p.: 108–110 °C (dec) (EtOAc). ^1^H NMR (600 MHz, CDCl_3_) δ (ppm): 10.38 (s, br,1H,N*H*CO, D_2_Oexchange), 8.69 (d, *J* = 8.8 Hz, 1H, H-3), 8.52 (s, br, 1H, N*H*CH*_2_*CH_2_, D_2_Oexchange), 8.24 (d, *J*= 2.5 Hz, 1H, H-9), 7.35 (d, *J* = 9.8 Hz, 1H, H-6), 7.29 (dd, *J* = 9.7, 2.6 Hz, 1H, H-7), 6.45 (d, *J* = 8.9 Hz, 1H, H-2), 3.89 (s, 3H, OC*H*_3_), 3.51 (q, *J* = 4.8 Hz, 2H, NHC*H_2_*CH_2_), 3.17 (s, 2H, COC*H*_2_), 2.89 (t, 2H, *J* = 6.0 Hz, NHCH_2_C*H*_2_), 2.71 (s, 6H, COCH_2_N(C*H_3_*)_2_), 2.45 (s, 6H, NHCH_2_CH_2_N(C*H_3_*)_2_). ^13^C NMR (151 MHz, MeOD-*d_4_*) δ (ppm): (NH*C*O), 161.0 (*C*O), 150.7 (C-8), 147.8 (C-5a), 146.6 (C-4a), 142.4 (C-1), 133.3 (C-7), 128.8 (C-6), 127.7 (C-3), 121.6 (C-4), 106.2 (C-9), 103.5 (C-2), 101.2 (C-11a), 60.3 (CO*C*H_2_), 57.2 (NHCH_2_*C*H*_2_*), 56.8 (O*C*H_3_), 44.6 (NHCH_2_CH_2_N(*C*H*_3_*)_2_), 44.1 (COCH_2_N(*C*H*_3_*)_2_), 39.2 (NH*C*H_2_CH_2_). *m*/*z*: calcd. for C_21_H_29_N_6_O_3_^+^: [M1 + H]^+^ = 413.2296, found 413.2290.

**2-Diethylamino-N-(1-((3-(diethylamino)propyl)amino)-8-methoxy-11-oxo-11*H*-pyrido[2,1-*b*]quinazolin-4-yl)acetamide (40)**

This compound was synthesized using a procedure analogous to that described for the preparation of **38**, using compound **16** and diethylamine. Yield: 29%. M.p.: 148–150 °C (dec) (EtOAc). ^1^H NMR (400 MHz, CDCl_3_) δ (ppm): 10.77 (brs, 1H, D_2_O exch., N*H*CO), 8.75 (d, *J* = 9.3 Hz, 1H, H-3), 8.39 (brs, 1H, D_2_O exch., N*H*CH*_2_*CH_2_CH_2_, D_2_Oexchange), 8.19 (s, 1H, H-9), 7.37 (d, *J* = 9.7 Hz, 1H, H-6), 7.30 (dd, *J* = 9.8, 2.6 Hz, 1H, H-7), 6.40 (d,*J* = 11.5 Hz, 1H, H-2), 6.43 (d, *J* = 10 Hz, 1H, H-2), 3.91 (s, 3H, OC*H*_3_), 3.41 (q, *J* = 2.6 Hz, 2H, NHC*H_2_*CH*_2_*CH_2_), 3.12 (s, 2H, COC*H*_2_), 3.09 (m, 6H, NHCH_2_CH_2_C*H*_2_, CH_2_CH_2_CH_2_N(C*H_2_*CH*_3_*)_2_), 2.70 (q, *J* = 7.1 Hz, 4H, COCH_2_N(C*H_2_*CH*_3_*)_2_), 2.20 (quintet, *J* = 8.02 Hz, 2H, NHCH*_2_*C*H_2_*CH_2_), 1.36 (t, *J* = 7.3 Hz, 6H, CH_2_CH_2_CH_2_N(CH*_2_*C*H_3_*)_2_), 1.16 (t, *J* = 7.11 Hz, 6H, COCH_2_N(CH*_2_*C*H_3_*)_2_). ^13^C NMR (50 MHz, CDCl_3_) δ (ppm): 169.5 (NH*C*O), 160.050 (*C*O), 148.6 (C-8), 146.1 (C-5a), 144.5 (C-4a), 139.5 (C-1), 131.3 (C-7), 126.8 (C-6), 126.2 (C-3), 121.5 (C-4), 104.9 (C-9), 102.6 (C-2), 101.6 (C-11a), 56.3 (CO*C*H_2_), 49.5 (O*C*H_3_), 48.5 (NHCH_2_CH*_2_C*H*_2_*), 46.4 (COCH_2_CH_2_N(*C*H*_2_*CH*_3_*)_2_), 46.4 (CH_2_CH_2_CH_2_N(*C*H*_2_*CH*_3_*)_2_), 41.1 (NH*C*H*_2_*CH_2_CH_2_), 24.4 (NHCH*_2_C*H_2_CH_2_), 10.5 (COCH_2_CH_2_N(CH*_2_C*H*_3_*)_2_), 9.9 (CH_2_CH_2_CH_2_N(CH*_2_C*H*_3_*)_2_). *m*/*z*: calcd. for C_26_H_39_N_6_O_3_^+^: [M1 + H]^+^ = 483.3078, found 483.3084.

**2-Dimethylamino-*N*-(1-((3-(dimethylamino)propyl)amino)-8-methoxy-11-oxo-11*H*-pyrido[2,1-*b*]quinazolin-4-yl)acetamide (41)**

This compound was synthesized using a procedure analogous to that described for the preparation of **38**, using compound **17** and dimethylamine. Yield: 35%. M.p.: 210 °C (dec) (EtOAc). ^1^H NMR (600 MHz, CDCl_3_) δ (ppm): 10.37 (brs, 1H, D_2_O exch., N*H*CO), 8.69 (d, *J* = 8.8 Hz, 1H, H-3), 8.21 (s, *J* = 2.5 Hz, 1H, H-9), 7.38 (d, *J* = 9.8 Hz, 1H, H-6), 7.31 (dd, *J* = 9.8, 2.6 Hz, 1H, H-7), 6.40 (d, *J* = 8.9Hz, 1H,H-2), 3.91 (s, 3H, OC*H*_3_), 3.42 (q, *J* = 6.4 Hz, 2H, NHC*H_2_*CH_2_CH_2_), 3.20 (s, 2H, COC*H*_2_), 3.09 (t, *J* = 4.06 Hz, 2H, NHCH_2_CH_2_C*H*_2_), 2.76 (s, 6H, NHCH_2_CH_2_CH_2_N(C*H_3_*)_2_), 2.49 (s, 6H, COCH_2_N(C*H_3_*)_2_), 2.24 (quintet, *J* = 7.9 Hz, 2H, NHCH_2_C*H*_2_CH_2_). ^13^C NMR (50 MHz, CDCl_3_+1drop Acetone-*d6*) δ (ppm): 168.4 (NH*C*O), 160.0 (*C*O), 148.8 (C-8), 145.9 (C-5a), 144.6 (C-4a), 139.8 (C-1), 131.2 (C-7), 127.3 (C-6), 125.68 (C-3), 121.4 (C-4), 104.8 (C-9), 102.8 (C-2), 101.9 (C-11a), 64.2 (CO*C*H_2_), 56.3 (NHCH_2_CH*_2_C*H*_2_*), 56.1 (O*C*H_3_), 46.1 (COCH_2_N(*C*H*_3_*)_2_), 43.0 (NHCH_2_CH_2_CH_2_N(*C*H*_3_*)_2_), 40.4 (NH*C*H_2_CH*_2_*CH_2_), 24.2 (NHCH_2_*C*H*_2_*CH*_2_*). *m*/*z*: calcd. for C_22_H_31_N_6_O_3_^+^: [M1 + H]^+^ = 427.2452, found 427.2457.

**3-(Diethylamino)-*N*-(1-((2-(diethylamino)ethyl)amino)-11-oxo-11*H*-pyrido[2,1-*b*]quinazolin-4-yl)propanamide (50)**

This compound was synthesized using a procedure analogous to that described for the preparation of **34**, using compound **10**, 3-cloropropionyl chloride, and diethylamine. Yield: 32%. ^1^H NMR (400 MHz, CDCl_3_) δ (ppm): 9.87 (brs, 1H, D_2_O exch., N*H*CO), 8.69 (d, *J* = 7.3 Hz, 1H, H-3), 8.61 (d, *J* = 8.8 Hz, 1H, H-9), 8.43 (t, *J* = 4.6 Hz, 1H, D_2_O exch., N*H*CH_2_CH_2_), 7.45 (t, *J* = 5.1 Hz, 1H, H-7), 7.35 (d, *J* = 9.1 Hz, 1H, H-6), 6.76 (t, *J* = 6.4 Hz, 1H, H-8), 6.41 (d, *J* = 8.8 Hz, 2H, H-2), 3.19 (q, *J* = 6.5 Hz, 2H, NHC*H*_2_CH_2_), 2.89–3.07 (m, 10H, NHCH_2_C*H*_2_, COC*H*_2_C*H*_2_, NHCH_2_CH_2_N(C*H*_2_CH_3_)_2_, 2.77 (q, *J* = 7. 1Hz, 4H, COCH_2_CH_2_N(C*H*_2_CH_3_)_2_), 1.27 (t, *J* = 7.1 Hz, 6H, NHCH_2_CH_2_N(CH_2_C*H*_3_)_2_, 1.14 (t, *J* = 6.9 Hz, 6H, COCH_2_CH_2_N(CH_2_C*H*_3_)_2_). ^13^C NMR (50 MHz, CDCl_3_) δ (ppm): 169.7 (HN*C*O), 160.0 (CO), 146.7 (C-5a), 146.6 (C-4a), 139.5 (C-1), 134.3 (C-7), 126.9 (C-3), 126.5 (C-9), 125.7 (C-6), 121.4 (C-4), 111.9 (C-8), 102.9 (C-2), 101.6 (C-11a), 51.3 (NHCH_2_*C*H_2_,) 48.6 (COCH_2_*C*H_2_), 47.2 (COCH_2_CH_2_N(*C*H_2_CH_3_)_2_), 46.5 (NHCH_2_CH_2_N(*C*H_2_CH_3_)_2_), 41.1 (NH*C*H_2_CH_2_), 34.3(CO*C*H_2_CH_2_), 11.7 (COCH_2_CH_2_N(CH_2_*C*H_3_)_2_), 10.8 (NHCH_2_CH_2_N(CH_2_*C*H_3_)_2_). *m*/*z*: calcd. for C_25_H_37_N_6_O_2_^+^: [M1 + H]^+^ = 453.2973, found 453.2978.

**3-(Dimethylamino)-N-(1-((2-(dimethylamino)ethyl)amino)-11-oxo-11*H*-pyrido[2,1-*b*]quinazolin-4-yl)propanamide (51)**

This compound was synthesized using a procedure analogous to that described for the preparation of **34**, using compound **11**, 3-cloropropionyl chloride, and dimethylamine. Yield: 38%. M.p.: 258–260 °C (EtOAc). ^1^H NMR (400 MHz, CDCl_3_) δ(ppm): 10.25 (brs, 1H, D_2_O exch., N*H*CO), 8.64 (d, *J* = 7.3 Hz, 1H, H-3), 8.55 (d, *J* = 8.9 Hz, 1H, H-9), 8.37 (brs, 1H, D_2_O exch., 1H, N*H*CH_2_CH_2_CH_2_), 7.39 (t, *J* = 8.1 Hz, 1H, H-7), 7.27 (d, *J* = 7.8 Hz, 1H, H-6), 6.72 (t, *J* = 6.9 Hz, 1H, H-8), 6.34 (d, *J* = 9.0 Hz 1H, H-2), 3.29 (q, *J* = 7.0 Hz, 2H, NHC*H*_2_CH_2_), 2.82 (t, *J* = 6.6 Hz, 2H, COCH_2_C*H_2_*), 2.65–2.69 (m, 4H, NHCH_2_C*H*_2_, COC*H_2_*CH_2_,), 2.41 (s, 6H, CH_2_CH_2_CH_2_N(C*H*_3_)_2_), 2.28 (s, 6H, COCH_2_CH_2_N(C*H*_3_)_2_). ^13^C NMR (151 MHz, CDCl_3_) δ (ppm): 168.8 (HN*C*O), 160.1 (*C*O), 146.8 (C-5a), 146.7 (C-4a), 139.7 (C-1), 134.6 (C-7), 126.8 (C-3), 126.5 (C-9), 125.9 (C-6), 121.3 (C-4), 112.9 (C-8), 102.8 (C-2), 101.7 (C-11a), 57.8 (NHCH_2_*C*H_2_), 54.7 (COCH_2_*C*H_2_), 45.4 (COCH_2_CH_2_N(*C*H_3_)_2_), 44.6 (NHCH_2_CH_2_CH_2_N(*C*H_3_)_2_), 40.8 (NH*C*H_2_CH_2_), 34.2 (CO*C*H_2_CH_2_). *m*/*z*: calcd. for C_21_H_29_N_6_O_2_^+^: [M1 + H]^+^ = 397.2347, found 397.2341.

**3-(Diethylamino)-** **N-(1-((3-(diethylamino)propyl)amino)-11-oxo-11*H*-pyrido[2,1-*b*]quinazolin-4-yl)propanamide (52)**

This compound was synthesized using a procedure analogous to that described for the preparation of **34**, using compound **12**, 3-cloropropionyl chloride, and diethylamine. Yield: 40%. ^1^H NMR (400 MHz, CDCl_3_) δ (ppm): 10.70 (brs, 1H, D_2_O exch., N*H*CO), 8.78 (d, *J* = 8.9 Hz, 1H, H-3), 8.69 (d, *J* = 6.9 Hz, 1H, H-9), 8.34 (brs, 1H, D_2_O exch., N*H*CH_2_CH_2_CH_2_), 7.38 (t, *J* = 8.4, 7.3 Hz, 1H, H-7), 7.27 (d, *J* = 7.7 Hz, 1H, H-6), 6.71 (t, *J* = 6.3 Hz, 1H, H-8), 6.42 (d, *J*= 8.7 Hz, 1H, H-2), 3.25 (q, *J* = 5.9 Hz, 2H, NHC*H*_2_CH_2_CH_2_), 2.82 (t, *J* = 6.3 Hz, 2H, COCH_2_C*H_2_*), 2.67 (q, *J* = 7.0 Hz, 4H, CH_2_CH_2_CH_2_N(C*H*_2_CH_3_)_2_) 2.50–2.58 (m, 8H, NHCH_2_CH_2_C*H*_2_, COC*H_2_*CH_2_, COCH_2_CH_2_N(C*H*_2_CH_3_)_2_), 1.86 (quintet, *J* = 6.9 Hz, 2H, NHCH_2_C*H*_2_CH_2_), 1.10 (t, *J* = 7.1 Hz, 6H, CH_2_CH_2_CH_2_N(CH_2_C*H*_3_)_2_), 1.02 (t, *J* = 7.1 Hz, 6H, COCH_2_CH_2_N(CH_2_C*H*_3_)_2_). ^13^C NMR (50 MHz, CDCl_3_) δ (ppm): 170.6 (HN*C*O), 160.2 (*C*O), 146.9 (C-5a), 146.4 (C-4a), 139.5 (C-1), 134.1 (C-7), 127.2 (C-3), 126.4 (C-9), 125.8 (C-6), 121.7 (C-4), 111.9 (C-8), 103.1 (C-2), 101.5 (C-11a), 50.6 (NHCH_2_CH_2_*C*H_2_), 48.8 (COCH_2_*C*H_2_), 47.0 (COCH_2_CH_2_N(*C*H_2_CH_3_)_2_), 46.6 (CH_2_CH_2_CH_2_N(*C*H_2_CH_3_)_2_), 41.6 (NH*C*H_2_CH_2_CH_2_), 34.9 (CO*C*H_2_CH_2_), 26.6 (NHCH_2_*C*H_2_CH_2_), 11.8 (COCH_2_CH_2_N(CH_2_*C*H_3_)_2_)_,_ 11.5 (NHCH_2_CH_2_CH_2_N(CH_2_*C*H_3_)_2_). *m*/*z*: calcd. for C_26_H_39_N_6_O_2_^+^: [M1 + H]^+^ = 467.3129, found 467.3135.

**3-Dimethylamino-N-(1-((3-(dimethylamino)propyl)amino)-11-oxo-11*H*-pyrido[2,1-*b*]quinazolin-4-yl)propanamide (53)**

This compound was synthesized using a procedure analogous to that described for the preparation of **34**, using compound **13**, 3-cloropropionyl chloride, and dimethylamine. Yield: 24%. ^1^H NMR (400 MHz, CDCl_3_) δ (ppm): 10.68 (brs, 1H, D_2_O exch., N*H*CO), 8.73–8.78 (m, 2H, H-3, H-9), 8.36 (brs, 1H, D_2_O exch., NHCH_2_CH_2_CH_2_), 7.44 (t, *J* = 7.7 Hz, 1H, H-7), 7.34 (d, *J* = 9.0 Hz, 1H, H-6), 6.77 (t, *J* = 6.9 Hz, 1H, H-8), 6.46 (d, *J* = 9.0 Hz, 1H, H-2), 3.31 (q, *J* = 6.2 Hz, 2H, NHC*H*_2_CH_2_CH_2_), 2.78 (t, *J* = 6.2 Hz, 2H, COCH_2_C*H_2_*), 2.65 (t, *J* = 6.2 Hz, 2H, COC*H_2_*CH_2_), 2.54 (t, *J* = 7.3 Hz, 2H, NHCH_2_CH_2_C*H*_2_), 2.44 (s, 6H, NHCH_2_CH_2_CH_2_N(C*H*_3_)_2_), 2.35 (s, 6H, COCH_2_CH_2_N(C*H*_3_)_2_), 1.95 (quintet, *J* = 14.2, 7.0 Hz, 2H, NHCH_2_C*H*_2_CH_2_). ^13^C NMR (151 MHz, CDCl_3_) δ (ppm): 169.8 (HNCO), 160.3 (CO), 146.8 (C-5a), 146.6 (C-4a), 139.6 (C-1), 134.3 (C-7), 127.1 (C-3), 126.5 (C-9), 126.1 (C-6), 121.7 (C-4), 112.1 (C-8), 103.2 (C-2), 101.6 (C-11a), 57.3 (NHCH_2_CH_2_*C*H_2_), 55.2 (COCH_2_*C*H_2_), 45.1 (COCH_2_CH_2_N(*C*H_3_)_2_), 45.1 (NHCH_2_CH_2_CH_2_N(*C*H_3_)_2_), 41.3 (NH*C*H_2_CH_2_CH_2_), 34.9 (CO*C*H_2_CH_2_), 26.6 (NHCH_2_CH_2_*C*H_2_). *m*/*z*: calcd. for C_22_H_31_N_6_O_2_^+^: [M1 + H]^+^ = 411.2503, found 411.2496.

**3-(Diethylamino)-*N*-(1-((2-(diethylamino)ethyl)amino)-8-methoxy-11-oxo-11*H*-pyrido[2,1-*b*]quinazolin-4-yl)propanamide (54)**

This compound was synthesized using a procedure analogous to that described for the preparation of **38**, using compound **14**, 3-cloropropionyl chloride, and diethylamine. Yield: 28%. M.p.: 128–130 °C (EtOAc). ^1^H NMR (600 MHz, CDCl_3_) δ (ppm): 9.66 (brs, 1H, D_2_O exch., N*H*CO), 8.51 (d, *J* = 8.9 Hz, 2H, H-3), 8.37 (brs, 1H, D_2_O exch., N*H*CH_2_CH_2_), 8.08 (d, *J* = 2.4 Hz, 1H, H-9), 7.27 (d, *J* = 9.7 Hz, 1H, H-6), 7.18 (dd, *J* = 9.7, 2.5 Hz, 1H, H-7), 6.30 (d, *J* = 11.9 Hz, 1H, H-2), 3.78 (s, 3H, OC*H*_3_), 3.40 (t, *J* = 4.1 Hz, 3H, NHC*H_2_*CH_2_), 3.12 (t, *J* = 6.8 Hz, 2H, COCH_2_C*H*_2_), 2.82–2.91 (m, 8H, NHCH_2_CH_2_N(C*H_2_*CH*_3_*)_2_), NHCH_2_CH_2_C*H*_2_, COC*H*_2_CH_2_), 2.73 (q, *J* = 7.1 Hz, 4H, COCH_2_CH_2_N(C*H_2_*CH*_3_*)_2_), 1.17 (t, *J* = 7.2 Hz, 6H, HNCH_2_CH_2_N(CH*_2_*C*H_3_*)_2_), 1.07 (t, *J* = 7.2 Hz, 6H, COCH_2_CH_2_N(CH*_2_*C*H_3_*)_2_). ^13^C NMR (151 MHz, CDCl_3_) δ (ppm): 168.4 (NH*C*O), 159.9 (*C*O), 148.9 (C-8), 146.2 (C-5a), 144.8 (C-4a), 139.9 (C-1), 131.6 (C-7), 126.9 (C-3), 126.0 (C-6), 121.5 (C-4), 105.2 (C-9), 102.7 (C-2), 101.8 (C-11a), 56.5 (O*C*H_3_), 51.0 (NHCH*_2_C*H*_2_*), 48.5 (COCH_2_*C*H_2_), 47.3 (COCH_2_CH_2_N(*C*H*_2_*CH*_3_*)_2_), 46.7 (NHCH_2_CH_2_N(*C*H*_2_*CH*_3_*)_2_), 40.4 (NH*C*H*_2_*CH_2_), 33.5 (CO*C*H_2_CH_2_), 10.9 (COCH_2_CH_2_N(CH*_2_C*H*_3_*)_2_), 9.8 (NHCH_2_CH_2_CH_2_N(CH*_2_C*H*_3_*)_2_). *m*/*z*: calcd. for C_26_H_39_N_6_O_3_^+^: [M1 + H]^+^ = 483.3078, found 483.3085.

**3-Dimethylamino-N-(1-((2-(dimethylamino)ethyl)amino)-8-methoxy-11-oxo-11*H*-pyrido[2,1-*b*]quinazolin-4-yl)propanamide (55)**

This compound was synthesized using a procedure analogous to that described for the preparation of **38**, using compound **15**, 3-cloropropionyl chloride, and dimethylamine. Yield: 20%. M.p.: 202–204 °C (EtOAc). ^1^H NMR (600 MHz, CDCl_3_) δ (ppm): 10.33 (brs, 1H, D_2_O exch., N*H*CO), 8.72 (d, *J* = 8.8 Hz, 1H, H-3), 8.51 (t, *J* = 4.2 Hz, 1H, D_2_O exch., N*H*CH_2_CH_2_e), 8.25 (d, *J* = 2.5 Hz, 1H, H-9), 7.35 (d, *J* = 9.74 Hz, 1H, H-6), 7.29 (dd, *J* = 9.7, 2.6 Hz, 1H, H-7), 6.42 (d, *J* = 8.9 Hz, 1H, H-2), 3.88 (s, 3H, OC*H*_3_), 3.37 (q, *J* = 5.5 Hz, 2H, NHC*H_2_*CH_2_), 2.94 (t, *J* = 6.3 Hz, 2H, COCH_2_C*H*_2_), 2.77 (t, *J* = 6.4 Hz, 2H, NHCH*_2_*C*H*_2_), 2.72 (t, *J* = 6.3 Hz, 2H, COC*H*_2_CH_2_), 2.52 (s, 6H, HNCH_2_CH_2_N(C*H_3_*)_2_), 2.38 (s, 6H, COCH_2_CH_2_N(C*H_3_*)_2_). ^13^C NMR (151 MHz, CDCl_3_) δ (ppm): 169.0 (NH*C*O), 159.9 (*C*O), 148.6 (C-8), 146.3 (C-5a), 144.6 (C-4a), 139.5 (C-1), 131.3 (C-7), 126.9 (C-6), 126.1 (C-3), 121.5 (C-4), 105.2 (C-9), 102.8 (C-2), 101.6 (C-11a), 57.8 (NHCH_2_*C*H*_2_*), 56.4 (O*C*H_3_), 54.7 (COCH_2_*C*H_2_) 45.3 (COCH_2_CH_2_N(*C*H*_3_*)_2_) 44.6 (HNCH_2_CH_2_N(*C*H*_3_*)_2_) 40.8 (NH*C*H*_2_*CH_2_), 34.4 (CO*C*H_2_CH_2_). *m*/*z*: calcd. for C_22_H_31_N_6_O_3_^+^: [M1 + H]^+^ = 427.2452, found 427.2457.

**3-Diethylamino-N-(1-((3-(diethylamino)propyl)amino)-8-methoxy-11-oxo-11*H*-pyrido[2,1-*b*]quinazolin-4-yl)propanamide (56)**

This compound was synthesized using a procedure analogous to that described for the preparation of **38**, using compound **16**, 3-cloropropionyl chloride, and dimethylamine. Yield: 10%. ^1^H NMR (600 MHz, CDCl_3_) δ (ppm): 10.26 (brs, 1H, D2O exch., NHCO), 8.78 (d, *J* = 8.9 Hz, 1H, H-3), 8.59 (brs, 1H, D_2_O exch., N*H*CH*_2_*CH_2_CH_2_), 8.22 (d, *J* = 2.56 Hz 1H, H-9), 7.37 (d, *J* = 9.7 Hz,1H, H-6), 7.31 (dd, *J* = 9.8, 2.6 Hz, 1H, H-7), 6.39 (d, *J* = 9.3 Hz, 1H, H-2), 6.43 (d, *J* = 8.9 Hz, 1H, H-2), 3.90 (s, 3H, OC*H*_3_), 3.35 (t, *J* = 6.6 Hz, 2H, NHC*H_2_*CH*_2_*CH_2_), 3.03 (t, *J* = 6.6 Hz, 2H, COCH_2_C*H*_2_), 2.82–2.93 (m, 10H, NHCH_2_CH_2_C*H*_2_, NHCH_2_CH_2_CH_2_N(C*H_2_*CH*_3_*)_2_, COCH_2_CH_2_N(C*H_2_*CH*_3_*)_2_), 2.77 (t, *J* = 6.6 Hz, 2H,COC*H*_2_CH_2_), 2.06 (quintet, *J* = 7.6 Hz, 2H, NHCH*_2_*C*H_2_*CH_2_), 1.20–1.24 (m, 12H, NHCH_2_CH_2_CH_2_N(CH*_2_*C*H_3_*)_2_, COCH_2_CH_2_N(CH*_2_*C*H_3_*)_2_). ^13^C NMR (151 MHz, CDCl_3_) δ (ppm): 169.5 (NH*C*O), 160.0 (*C*O), 148.7 (C-8), 146.1 (C-5a), 144.5 (C-4a), 139.5 (C-1), 131.3 (C-7), 126.2 (C-3), 126.8 (C-6), 121.8 (C-4), 104.9 (C-9), 102.8 (C-2), 101.6 (C-11a), 56.3 (O*C*H_3_), 49.5 (NHCH_2_CH*_2_C*H*_2_*), 48.5 (COCH_2_*C*H_2_), 46.4 (COCH_2_CH_2_N(*C*H*_2_*CH*_3_*)_2_), 46.4 (NHCH_2_CH_2_CH_2_N(*C*H*_2_*CH*_3_*)_2_), 41.1 (NH*C*H*_2_*CH_2_CH_2_), 34.1 (CO*C*H_2_CH_2_), 24.4 (NHCH*_2_C*H_2_CH_2_), 10.5 (COCH_2_CH_2_N(CH*_2_C*H*_3_*)_2_), 9.9 (NHCH_2_CH_2_CH_2_N(CH*_2_C*H*_3_*)_2_). *m*/*z*: calcd. for C_27_H_41_N_6_O_3_^+^: [M1 + H]^+^ = 497.3235, found 497.3228.

**3-Dimethylamino-*N*-(1-((3-(dimethylamino)propyl)amino)-8-methoxy-11-oxo-11*H*-pyrido[2,1-*b*]quinazolin-4-yl)propanamide (57)**

This compound was synthesized using a procedure analogous to that described for the preparation of **38**, using compound **17**, 3-cloropropionyl chloride, and dimethylamine. M.p.: 178–180 °C (dec) (EtOAc). ^1^H NMR (400 MHz, CDCl_3_) δ (ppm): 10.34 (brs, 1H, D_2_O exch., N*H*CO), 8.64 (d, *J* = 8.9 Hz, 1H), 8.3 (t, *J* = 8.4 Hz 1H, D_2_O exch., N*H*CH_2_CH_2_CH_2_), 8.19 (s, *J* = 2.3 Hz, 1H, H-9), 7.34 (d, *J* = 9.8 Hz, 1H, H-6), 7.29 (dd, *J* = 9.8, 2.5 Hz, 1H, H-7), 6.41 (d, *J* = 8.9 Hz, 1H, H-3), 3.89 (s, 3H, OC*H*_3_), 3.31 (q, *J* = 7.2 Hz, 2H, NHC*H_2_*CH_2_CH_2_), 2.87 (t, *J* = 6.3 Hz, 2H, COCH_2_C*H*_2_), 2.67–2.72(m, 4H, NHCH*_2_*CH_2_C*H*_2_, COC*H*_2_CH_2_), 2.47 (s, 6H, NHCH_2_CH_2_CH_2_N(C*H_3_*)_2_), 2.43 (s, 6H, COCH_2_CH_2_N(C*H_3_*)_2_), 2.02 (quintet, *J* = 6.9 Hz, 2H, NHCH*_2_*C*H*_2_CH_2_). ^13^C NMR (151 MHz, CDCl_3_) δ (ppm): 169.9 (NH*C*O), 160.0 (*C*O), 148.6 (C-8), 146.3 (C-5a), 144.4 (C-4a), 139.4 (C-1), 131.1 (C-7), 126.9 (C-6), 126.3 (C-3), 121.7 (C-4), 104.9 (C-9), 103.0 (C-2), 101.6 (C-11a), 57.3 (NHCH_2_CH_2_*C*H*_2_*), 56.3 (O*C*H_3_), 55.2 (COCH_2_*C*H_2_), 45.1 (NHCH_2_CH_2_CH_2_N(*C*H*_3_*)_2_, COCH_2_CH_2_N(*C*H*_3_*)_2_) 41.3 (NH*C*H*_2_*CH_2_CH_2_), 34.9 (CO*C*H_2_CH_2_), 26.6 (NHCH*_2_C*H_2_CH_2_). *m*/*z*: calcd. for C_23_H_33_N_6_O_3_^+^: [M1 + H]^+^ = 441.2609, found 441.2616.

### 3.3. Cell Lines—Growth Conditions—Cytotoxicity Assays

The T24 human urinary/urothelial bladder cancer cell line (major mutation signature: H-RAS^G12V^; p53^ΔY126^) was provided by ATCC-LGC Standards GmbH (Wesel, Germany), and the WM266-4 human metastatic melanoma (skin cancer) cell line (major mutation signature: B-RAF^V600D^) was purchased from ECACC-Sigma-Aldrich (St. Louis, MO, USA). Cells were cultured and exponentially grown in a complete 1x DMEM medium supplemented with 10% (heat-inactivated) FBS in a 5% CO_2_ environment and at +37 °C. For the MTT cytotoxicity assay, cells were seeded onto 48-well plates at approximately 90% confluency and subsequently treated with different doses of the four compounds (11, 15, 35, and 39) for 24 h. Next, the cells were incubated with MTT solution for 4 h, and the formazan crystals produced were carefully dissolved in isopropanol. Spectrophotometric absorbance was measured using a Dynatech MR5000 Elisa microtiter-plate reader (Dynatech Laboratories, Chantilly, VA, USA) at 550 nm, using 630 nm as the reference wavelength. Each MTT assay was performed in triplicate, using three wells per tested compound and cell line. All four compounds were dissolved in high-grade DMSO. The statistical significance of the differences observed in the compound-treated versus control (DMSO) cell survival values was determined using an unpaired two-sided Student’s *t*-test. Data are reported as mean ± SD (standard deviation) of the mean value. *p < 0.001* was considered statistically significant.

### 3.4. Hydrolysis Study Experimental

#### 3.4.1. Samples Preparation

Each compound (10 mg) was weighed in a 10 mL volumetric flask, diluted with LC-MS grade acetonitrile, and stored at −20 °C in amber glass vials (concentration 1000 mg L^−1^). Intermediate working solutions of 5.0–6.25–7.5–8.75–10.0–12.5–15.0 mg L^−1^ were prepared by appropriate dilutions of the stock solutions in ultrapure water.

#### 3.4.2. Instrumental Analysis

The analysis was performed using a UV spectrometer (SHIMADZU UV-1800, Shimadzu Corporation, Kyoto, Japan) equipped with a 150W Xenon lamp. The spectra were obtained from 200–500 nm in the scan mode using a spectral resolution of 1 nm, whereas the validation was performed using the photometry mode.

#### 3.4.3. Validation of the Analytical Method

A six-point calibration curve was constructed by preparing working solutions of the investigated compounds at various concentrations (xx mg L^−1^) to examine the linear range. Furthermore, the precision of the method (repeatability and intermediate precision) was assessed. Statistical calculations were performed using Microsoft Excel 365.

#### 3.4.4. Kinetic Study

The spectra of the compounds were obtained at the optimum λ_max_ at different time points to investigate the extent of hydrolysis. Specifically, the spectra were obtained at the following time points: t = 0, 5, 10, 20, 60, 90, and 120 min, based on the estimated t_1/2_ of the compounds.

### 3.5. Computational Chemistry

The molecules were drawn, displayed, and characterized using Marvin Sketch software (Marvin 24.3.0, 2024, ChemAxon (http://www.chemaxon.com)) and optimized using the Gaussian09 computational chemistry package [39]. The 6-31G basis set was employed using the B3LYP approximation using DFT calculations for the geometry optimization of the two molecules at the ground state, and a full NBO analysis was also carried out. The results were also punched to the corresponding wfn files using the output=wfn additional command and pop=(3/33=4) for executing the population analysis. Electronic density post-processing was performed using the MultiWFN program [40,41], using topology, fuzzy atomic space, basin analysis, electron delocalization, and aromaticity analyses. The FUKUI functions were calculated using the UCA-FUKUI v.2 software [42]. All simple arithmetic analyses were performed using Microsoft Excel 365.

## 4. Conclusions

In summary, we designed and synthesized a series of novel amino-substituted aza-acridines and evaluated their in vitro anticancer properties. Among these, compounds **11** and **15** exhibited low to moderate cytotoxic activity, whereas compounds **35** and **39** demonstrated more pronounced effects against WM266-4 metastatic melanoma cells but not against T24 bladder cancer cells. While these findings indicate a degree of selectivity toward melanoma cells, further investigation is needed to assess their stability, mechanism of action and potential relevance in more advanced biological models.

To better understand the observed activity profile, we focused on the stability of the compounds, given previous evidence from our group indicating that the aza-acridine core is prone to hydrolysis. To explore this, we selected compounds **25** and **29**, which differ by a methoxy substituent at position 2 of the core, and examined them using mass spectrometry and DFT-based computational analysis. Both compounds were highly stable under the experimental conditions, underscoring the influence of core substitution on chemical stability. This combined experimental and theoretical approach offers valuable insights into the behavior of these analogs and could guide the future design of more stable and potent aza-acridine derivatives.

## Figures and Tables

**Table 1:** The angles and dihedral angles of molecules 1A and 9A, along with their corresponding differences from their theoretical values.

Angles				Difference from the theoretical 120°	
	1A		9A	1A	9A
C7-N3-C6	120.31	C6-N7-C26	118.52	−0.31	1.48
C7-N3-C1	118.04	C6-N7-C8	120.79	1.96	−0.79
C6-N3-C1	121.64	C26-N7-C8	120.67	−1.64	−0.67
C8-C7-N3	115.99	C23-C6-N7	116.74	4.01	3.26
C8-C7-O18	126.22	C23-C5-O14	122.17	−6.22	−2.17
N3-C7-O18	117.78	N7-C6-O14	121.06	2.22	−1.06
C11-C8-C7	119.84	C1-C23-C6	119.94	0.16	0.06
C11-C8-C10	120.65	C1-C23-C5	120.21	−0.65	−0.21
C7-C8-C10	119.49	C3-C2-C5	119.91	0.51	0.09
				Difference from the theoretical 180°	
C11-C8-C7-N3	−178.52	C1-C23-C6-N7	−179.45	1.48	0.55
C8-C7-N3-C1	−179.83	C23-C6-N7-C8	−177.16	0.17	2.84

**Table 2:** Selected bond lengths of the 1A and 9A derivatives along with their highest differences. The bond lengths contracted by 1A compared to 9A are highlighted in red, whereas the opposite phenomenon is highlighted in green.

1A		9A		
Number	Bond Length	Number	Bond Length	ΔBond (1A–9A)
N3-C7	1.435	N7-C6	1.446	−0.011
C7-O18	1.264	C6-O14	1.261	0.003
C8-C7	1.438	C23-C6	1.445	−0.007
C10-C8	1.437	C5-C23	1.415	0.022
C10-N9	1.369	C5-N25	1.374	−0.005
N9-C6	1.321	N25-C26	1.307	0.014
C6-N3	1.412	C26-N7	1.466	−0.054
N3-C1	1.392	N7-C8	1.415	−0.023
C8-C11	1.44	C23-C1	1.413	0.027
C1-C2	1.362	C8-C9	1.397	−0.035
C2-C4	1.431	C9-C24	1.389	0.042
C4-C5	1.368	C24-C10	1.391	−0.023
C5-C6	1.432	C10-C26	1.437	−0.005
C6-N9	1.321	C26-N25	1.307	0.014
C10-C13	1.42	C5-C4	1.413	0.007
C13-C14	1.399	C4-C3	1.386	0.013
C14-C12	1.384	C3-C2	1.416	−0.032
C12-C11	1.413	C2-C1	1.385	0.028

**Table 3:** Aromaticity indices of the 1A and 9C derivatives, along with their corresponding differences. (Harmonic Oscillator Model of Aromaticity), PDI: Para-Delocalization Index, FLU: Aromatic Fluctuation Index, HOMA: Harmonic Oscillator Model of Aromaticity).

	1A			9A			Difference		
Aromaticity Index	Ring B	Ring A	Ring C	Ring B	Ring A	Ring C	Ring B	Ring A	Ring C
PDI	0.0366	0.0547	0.0629	0.0344	0.0792	0.0425	0.0022	−0.0245	0.0204
FLU	0.0444	0.0178	0.0309	0.0526	0.0093	0.0375	−0.0082	0.0085	−0.0066
Shannon aromaticity	0.7069	0.6762	0.6972	0.7070	0.6760	0.6976	−0.0001	0.0003	−0.0004
HOMA	0.5120	0.7040	0.6400	0.3300	0.8810	0.5190	0.1820	−0.1770	0.1210

## Data Availability

All data are available in this publication and Supplementary Materials.

## Supplementary Materials

The following supporting information can be downloaded at: https://www.mdpi.com/article/10.3390/molecules30122612/s1, Table S1: Linearity results of the investigated compounds; Table S2: Repeatability results for the compound 16; Table S3: Repeatability results for the compound 17; Table S4: ELF LOL: ELF (Electron Localization Function) and LOL (Localized Orbital Locator) values of selected CPs of 1A and 9A along with their corresponding differences; Figure S1: Determination of IC_50_ values using MTT assay; Figure S2: Absorbance spectrum of the compound 16; Figure S3: Absorbance spectrum of the compound 17; Figure S4: CPs numbering of the 1A (A) and 9A(B) molecules as shown in Fig S ELFLOL; Figures S5–S48: 1H and 13C-NMR spectra of compounds.
