# Supplementary material for: Synthesis, Stability, and Biological Evaluation of Novel Aminoderivatives Incorporating the Aza-Acridine Scaffold
Source: Molecules. 2025 Jun 16;30(12):2612. doi: 10.3390/molecules30122612 (PMC12195947; doi:10.3390/molecules30122612)
Supplement: Supplementary file 1 [file molecules-30-02612-s001.zip › molecules-3674170-supplementary.pdf]

Supporting Information

Article

Synthesis, Stability, and Biological Evaluation of Novel Aminoderivatives Incorporating the Aza-acridine Scaffold

Maria Karelou[1], Anthi Panara[2], Eleftheria Chatziorfanou[1], Aikaterini F. Giannopoulou[3,4], Dimitrios J. Stravopodis[3], Evagelos Gikas[2], and Ioannis K. Kostakis[1,*]

1 Department of Pharmacy, Division of Pharmaceutical Chemistry, National and Kapodistrian University of Athens, Panepistimiopolis, Zografou, 15771 Athens, Greece; elgeorgiou@pharm.uoa.gr (E.G.); kon.paraskevas@outlook.com (K.P.).

2 Laboratory of Analytical Chemistry, Department of Chemistry, National and Kapodistrian University of Athens, Panepistimiopolis, Zografou, Athens 15771, Greece.

3 Laboratory of Cellular Oncology, Section of Cell Biology and Biophysics, Department of Biology, School of Science, National and Kapodistrian University of Athens, Panepistimiopolis, Zografou, 15771 Athens, Greece; aigiann@biol.uoa.gr (A.F.G.); dstravop@biol.uoa.gr (D.J.S.)

4 Present Address: Laboratory of Cell Biology of Immunity, Max Delbrück Center for Molecular Medicine (MDC), Robert-Rössle-Str. 10, 13125 Berlin, Germany; aikaterini.giannopoulou@mdc-berlin.de (A.F.G.)

* Correspondence: ikkostakis@pharm.uoa.gr (I.K.K.); Tel.: +30-210-727-4212

Contents

Table S1- Linearity results of the investigated compounds 4

Table S2: Repeatability results for the compound 16 4

Table S3: Repeatability results for the compound 17 4

Table S4: ELF LOL: ELF (Electron Localization Function) and LOL (Localized Orbital Locator) values of selected CPs of 1A and 9A along with their corresponding differences 4

Figure S1: Determination of IC_{50} values using MTT assay. 5

Figure S2: Absorbance spectrum of the compound 16 5

Figure S3: Absorbance spectrum of the compound 17 6

Figure S4: CPs numbering of the 1A (A) and 9A(B) molecules as shown in Fig S ELFLOL 7

Figure S5: ^{1}H NMR spectrum of 11 8

Figure S6: ^{13}C NMR spectrum of 11 8

Figure S7: ^{1}H NMR spectrum of 12 9

Figure S8: ^{13}C NMR spectrum of 12 9

Figure S9: ^{1}H NMR spectrum of 13 10

Figure S10: ^{13}C NMR spectrum of 13 10

Figure S11: ^{1}H NMR spectrum of 14 11

Figure S12: ^{13}C NMR spectrum of 14 11

Figure S13: ^{1}H NMR spectrum of 15 12

Figure S14: ^{13}C NMR spectrum of 15 12

Figure S15: ^{1}H NMR spectrum of 16 13

Figure S16: ^{13}C NMR spectrum of 16 13

Figure S17: ^{1}H NMR spectrum of 17 14

Figure S18: ^{13}C NMR spectrum of 17 14

Figure S19: ^{1}H NMR spectrum of 34 15

Figure S20: ^{13}C NMR spectrum of 34 15

Figure S21: ^{1}H NMR spectrum of 35 16

Figure S22: ^{13}C NMR spectrum of 35 16

Figure S23: ^{1}H NMR spectrum of 36 17

Figure S24: ^{13}C NMR spectrum of 36 17

Figure S25: ^{1}H NMR spectrum of 37 18

Figure S26: ^{13}C NMR spectrum of 37 18

Figure S27: ^{1}H NMR spectrum of 38 19

Figure S28: ^{13}C NMR spectrum of 38 19

Figure S29: ^{1}H NMR spectrum of 39 20

Figure S30: ^{13}C NMR spectrum of 39 20
Figure S31: ^{1}H NMR spectrum of 40 21
Figure S32: ^{13}C NMR spectrum of 40 21
Figure S33: ^{1}H NMR spectrum of 41 22
Figure S34: ^{13C} NMR spectrum of 41 22
Figure S35: ^{1}H NMR spectrum of 50 23
Figure S36: ^{13}C NMR spectrum of 50 23
Figure S37: ^{1}H NMR spectrum of 51 24
Figure S38: ^{13}C NMR spectrum of 51 24
Figure S39: ^{1}H NMR spectrum of 52 25
Figure S40: ^{13}C NMR spectrum of 52 25
Figure S41: ^{1}H NMR spectrum of 54 26
Figure S42: ^{13}C NMR spectrum of 54 26
Figure S43: ^{1}H NMR spectrum of 55 27
Figure S44: ^{13}C NMR spectrum of 55 27
Figure S45: ^{1}H NMR spectrum of 56 28
Figure S46: ^{13}C NMR spectrum of 56 28
Figure S47: ^{1}H NMR spectrum of 57 29
Figure S48: 13C NMR spectrum of 57 29

Compound name	Linear range (mg L^{-1})	Equation of the calibration curve	R^2
16	5.0-15.0	y= (7.81 ± 0.17) x× 10^{-2}-(22.00±1.7) ×10^{-2}	0.998
17	5.0-12.5	y= (6.82 ± 0.12) x×10^{-2}-(3.9±1.0) ×10^{-2}	0.9990

Table S1- Linearity results of the investigated compounds

	Absorbance of compound 16						
Concentration	5 mg L^{-1}	6.25 mg L^{-1}	7.5 mg L^{-1}	8.75 mg L^{-1}	10 mg L^{-1}	12.5 mg L^{-1}	15 mg L^{-1}
Repilcate 1	0.179	0.274	0.356	0.45	0.580	0.742	0.963
Repilcate 2	0.189	0.273	0.357	0.434	0.590	0.742	0.963
Repilcate 3	0.188	0.272	0.357	0.441	0.590	0.743	0.963
Repilcate 4	0.178	0.272	0.357	0.45	0.580	0.743	0.963
Repilcate 5	0.170	0.273	0.357	0.45	0.580	0.743	0.963
Average	0.181	0.273	0.357	0.445	0.584	0.742	0.963
SD	0.01	0.00	0.00	0.01	0.01	0.00	0.00
%RSD	4.34	0.31	0.13	1.64	0.94	0.07	0.00

Table S2: Repeatability results for the compound 16

	Absorbance of compound 17					
Concentration	5 mg L^{-1}	6.25 mg L^{-1}	7.5 mg $^{L-1}$	8.75 mg L^{-1}	10 mg L^{-1}	12.5 mg L^{-1}
Repilcate 1	0.304	0.385	0.483	0.555	0.641	0.813
Repilcate 2	0.304	0.385	0.483	0.554	0.641	0.817
Repilcate 3	0.304	0.385	0.484	0.553	0.641	0.819
Repilcate 4	0.304	0.385	0.485	0.553	0.641	0.821
Repilcate 5	0.304	0.385	0.485	0.556	0.64	0.822
Average	0.304	0.385	0.484	0.554	0.641	0.818
SD	0.00	0.00	0.00	0.00	0.00	0.00
%RSD	0.00	0.00	0.21	0.24	0.07	0.44

Table S3: Repeatability results for the compound 17

Difference 1A-9A	19	24	16	58	52
ELF CPs	-6.1×10^{-9}	6.0×10^{-9}	-1.69×10^{-8}	1.1×10^{-2}	2.64×10^{-2}
LOL CPs	-4.8×10^{-9}	2.4×10^{-9}	-8.8×10^{-9}	5.97×10^{-3}	2.1×10^{-3}

Table S4: ELF LOL: ELF (Electron Localization Function) and LOL (Localized Orbital Locator) values of selected CPs of 1A and 9A along with their corresponding differences

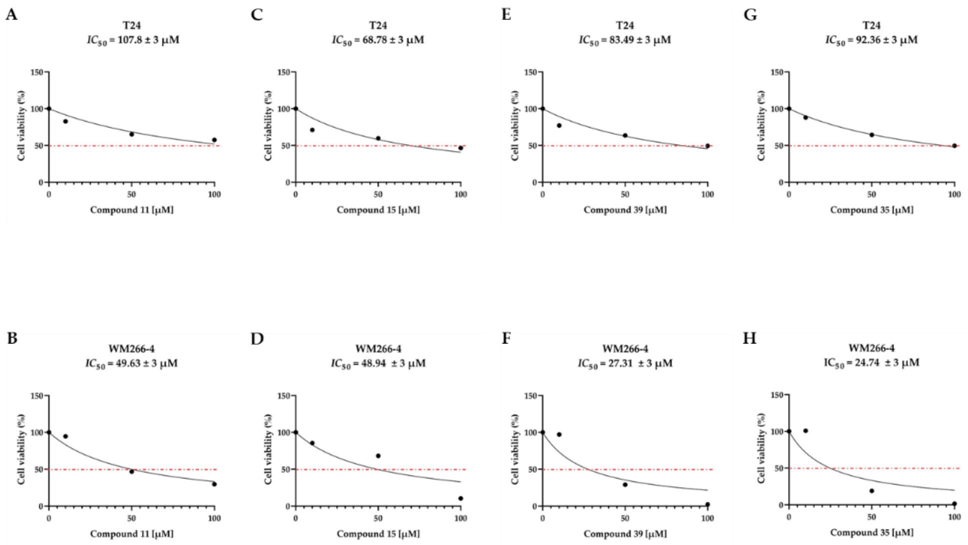

Figure S1: Determination of IC_{50} values using MTT assay.

Percentage of cell viability [%] is plotted on the Y-axis, while the concentration of the test compound [in µM] is shown on the X-axis. The IC_{50} value—defined as the concentration at which 50% inhibition of cell viability is observed—is determined as the point where the dose-response curve crosses the 50% viability level. Nonlinear regression (Inhibitor vs. normalized response) is used for curve fitting in GraphPad Prism 9.0.

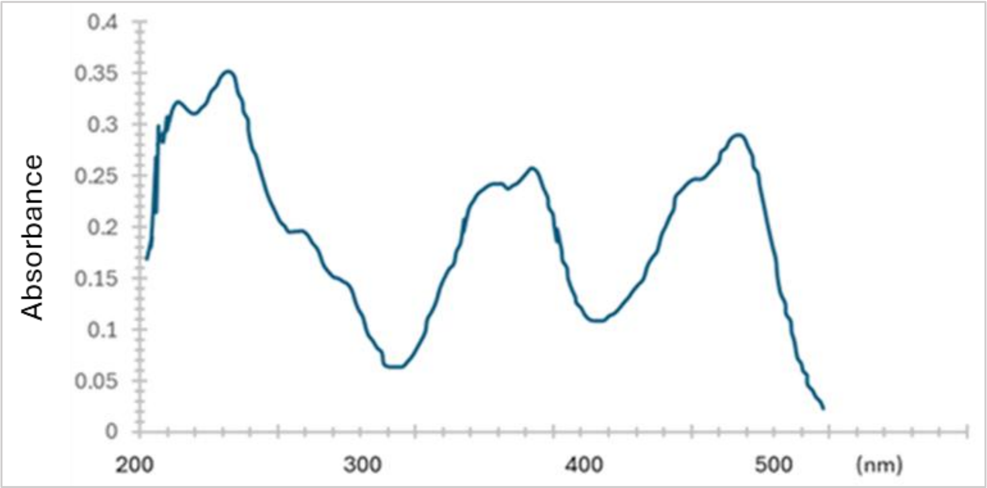

Figure S2: Absorbance spectrum of the compound 16

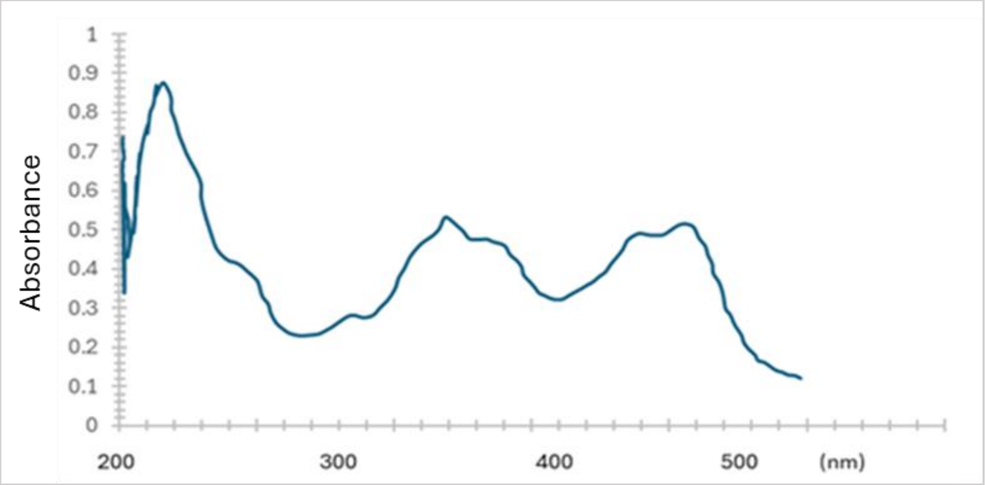

Figure S3: Absorbance spectrum of the compound 17

A

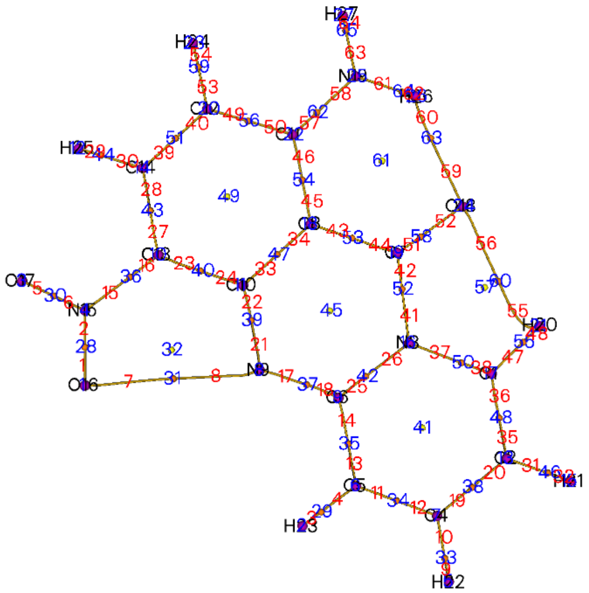

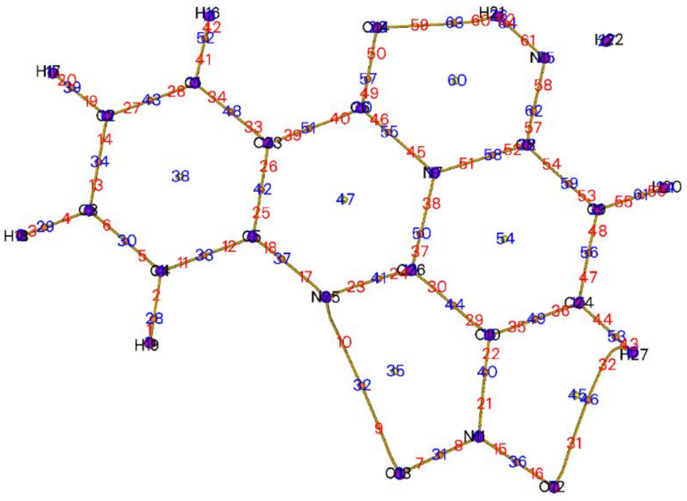

B

Figure S4: CPs numbering of the 1A (A) and 9A(B) molecules as shown in Fig S ELFLOL

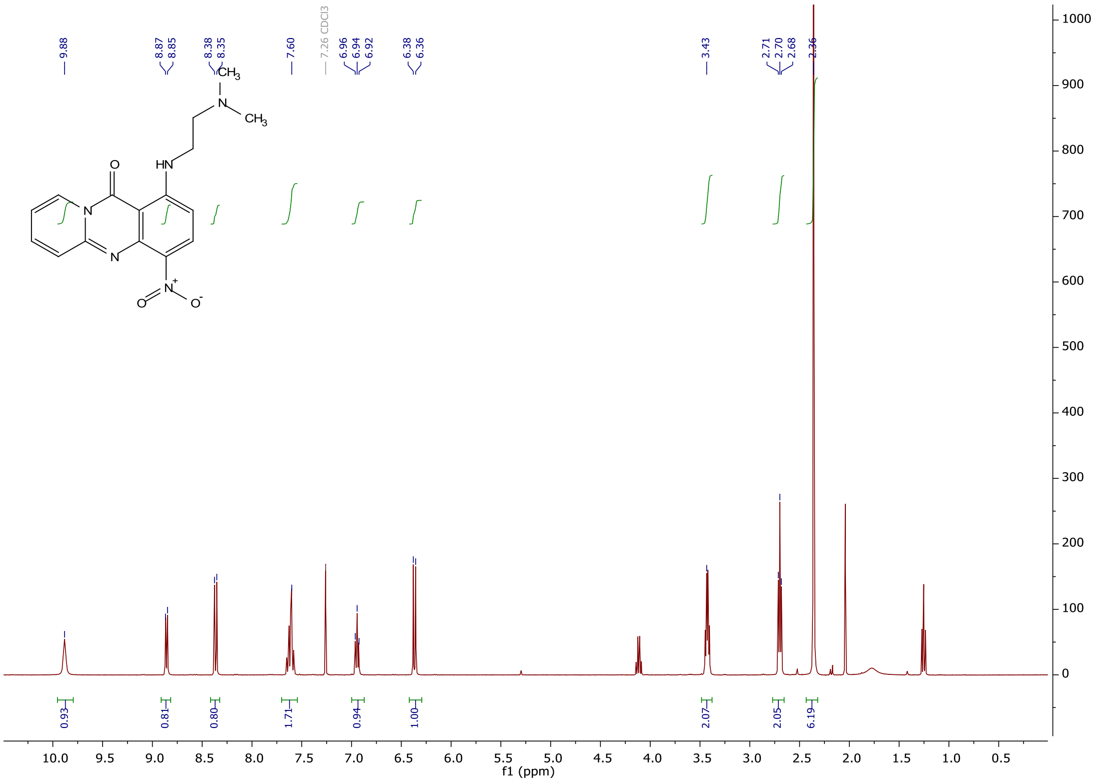

Figure S5: ^{1}H NMR spectrum of 11

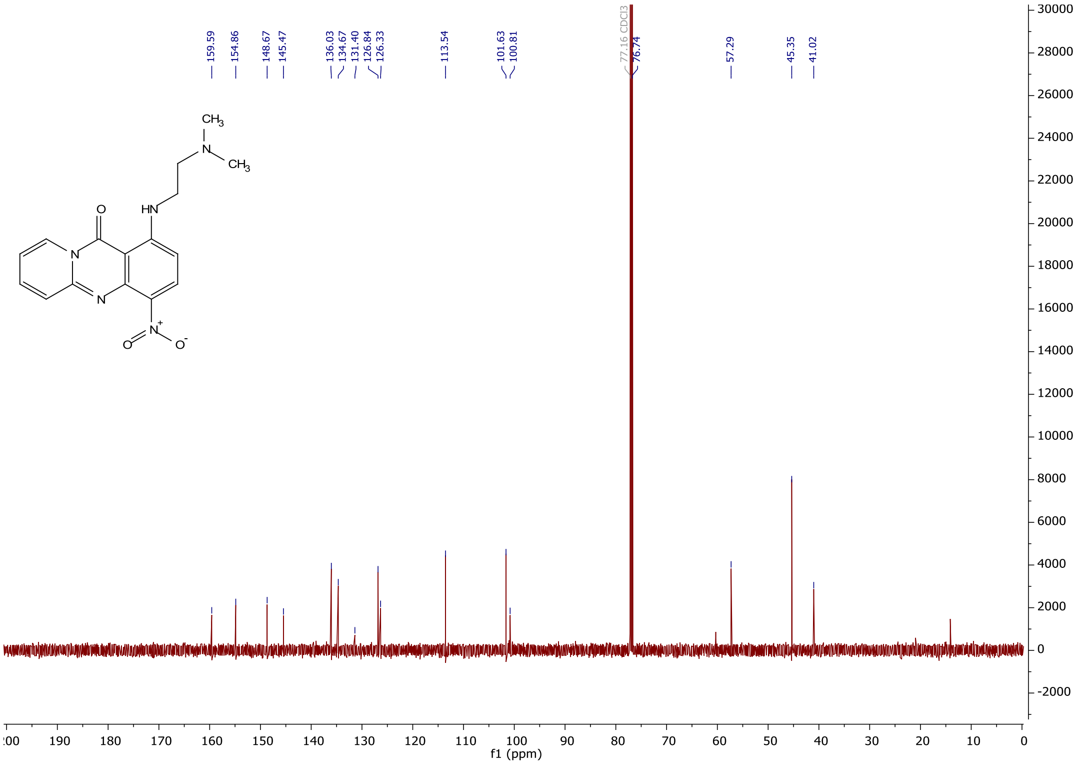

Figure S6: ^{13}C NMR spectrum of 11

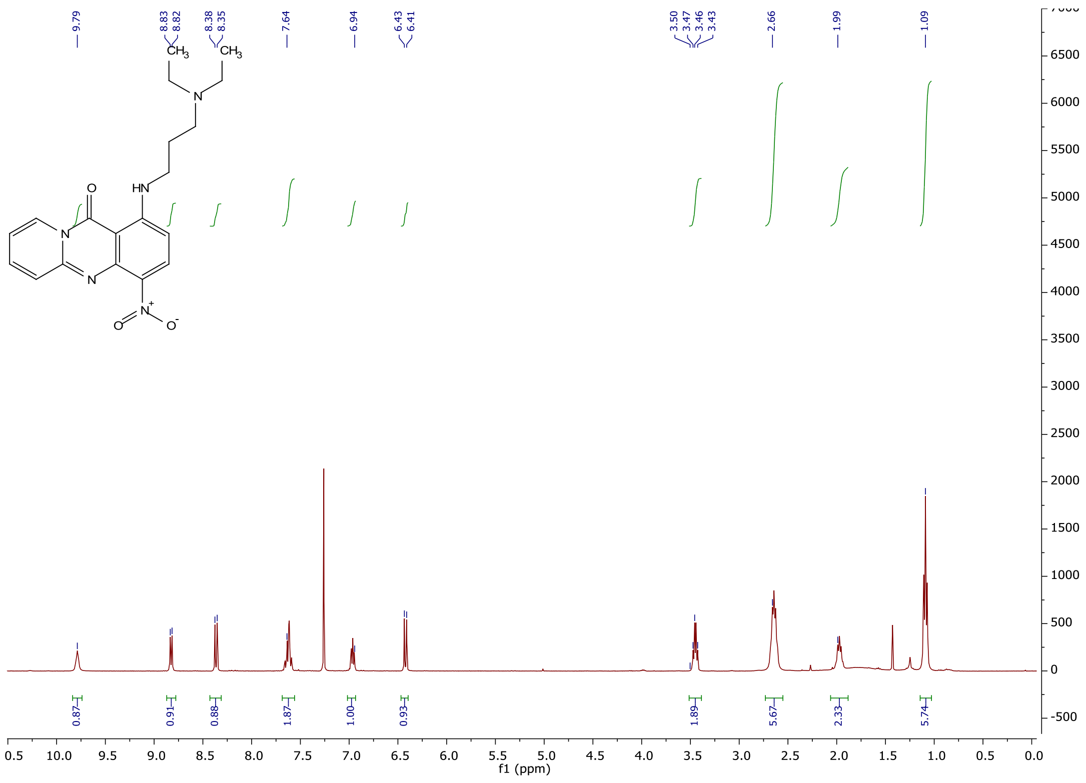

Figure S7: ^{1}H NMR spectrum of 12

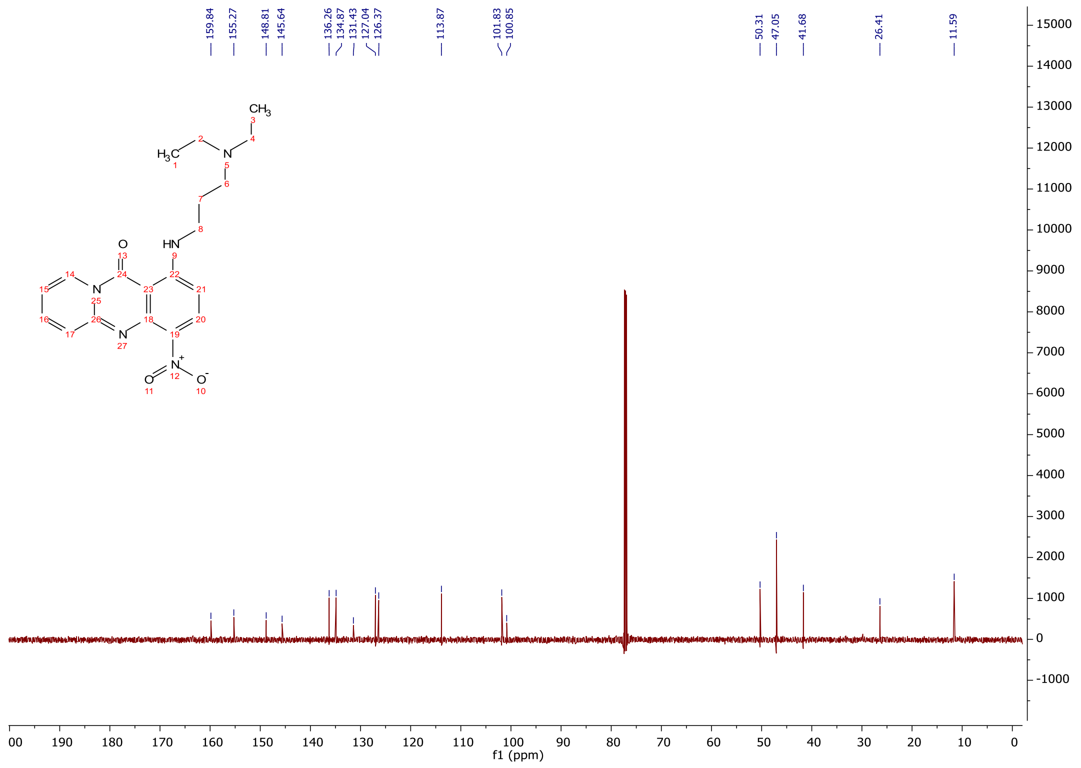

Figure S8: ^{13}C NMR spectrum of 12

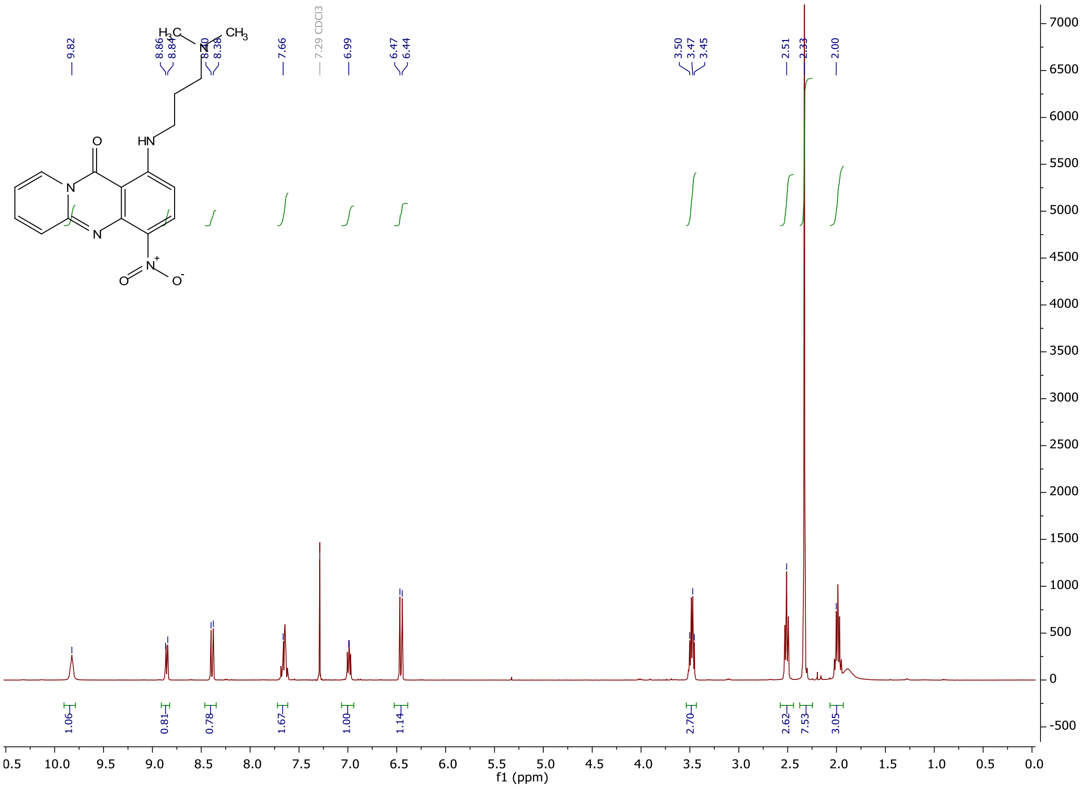

Figure S9: ^{1}H NMR spectrum of 13

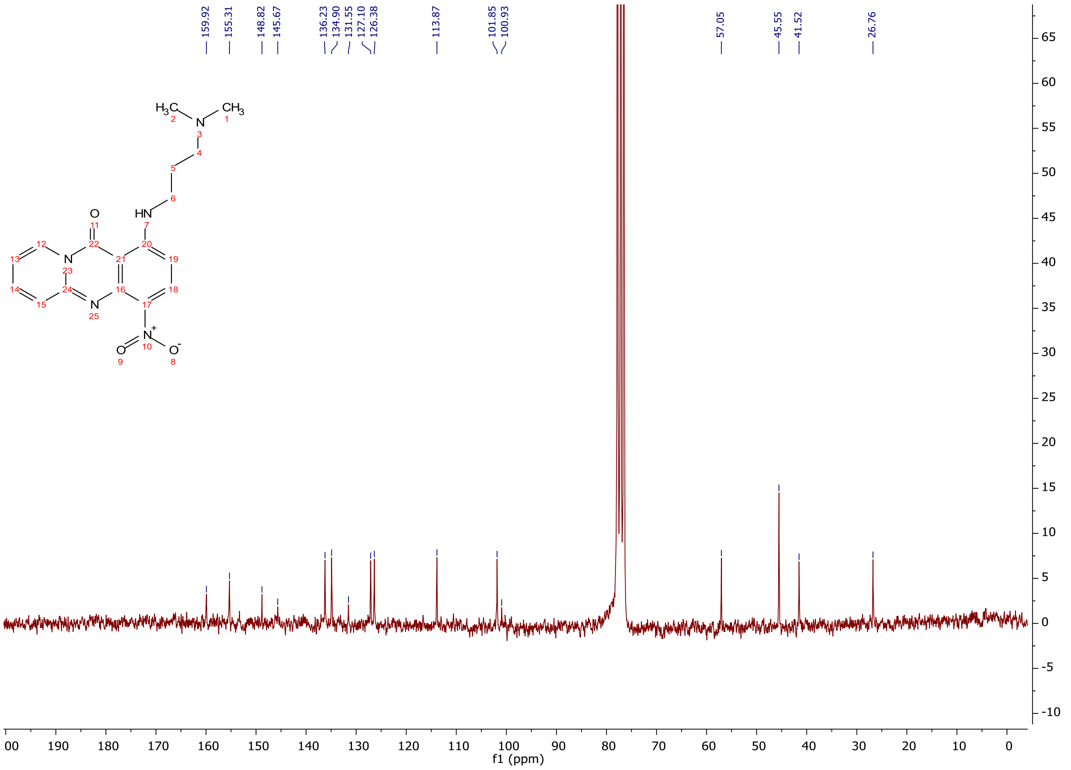

Figure S10: ^{13}C NMR spectrum of 13

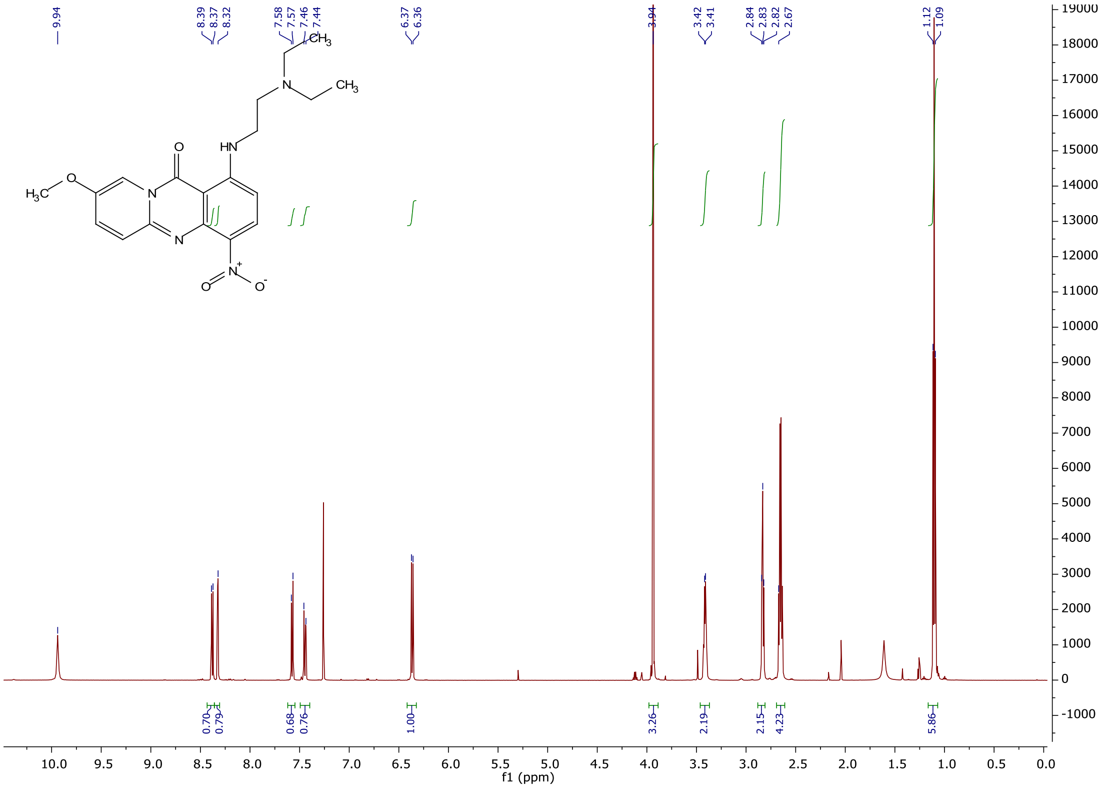

Figure S11: ^{1}H NMR spectrum of 14

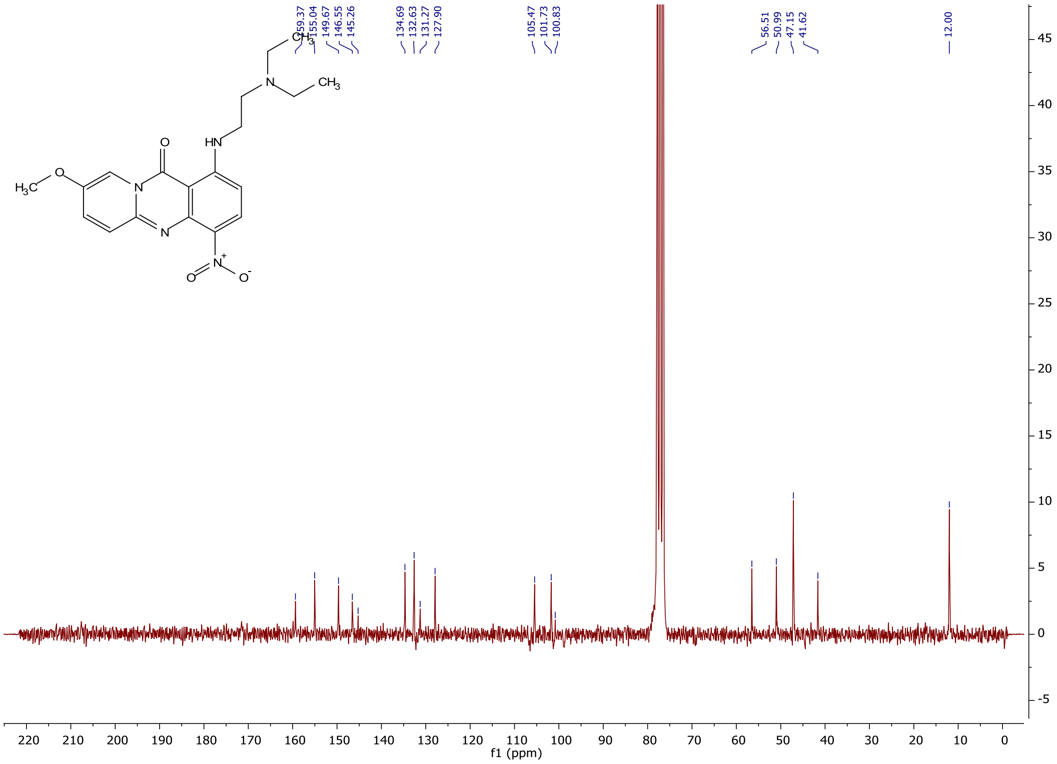

Figure S12: ^{13}C NMR spectrum of 14

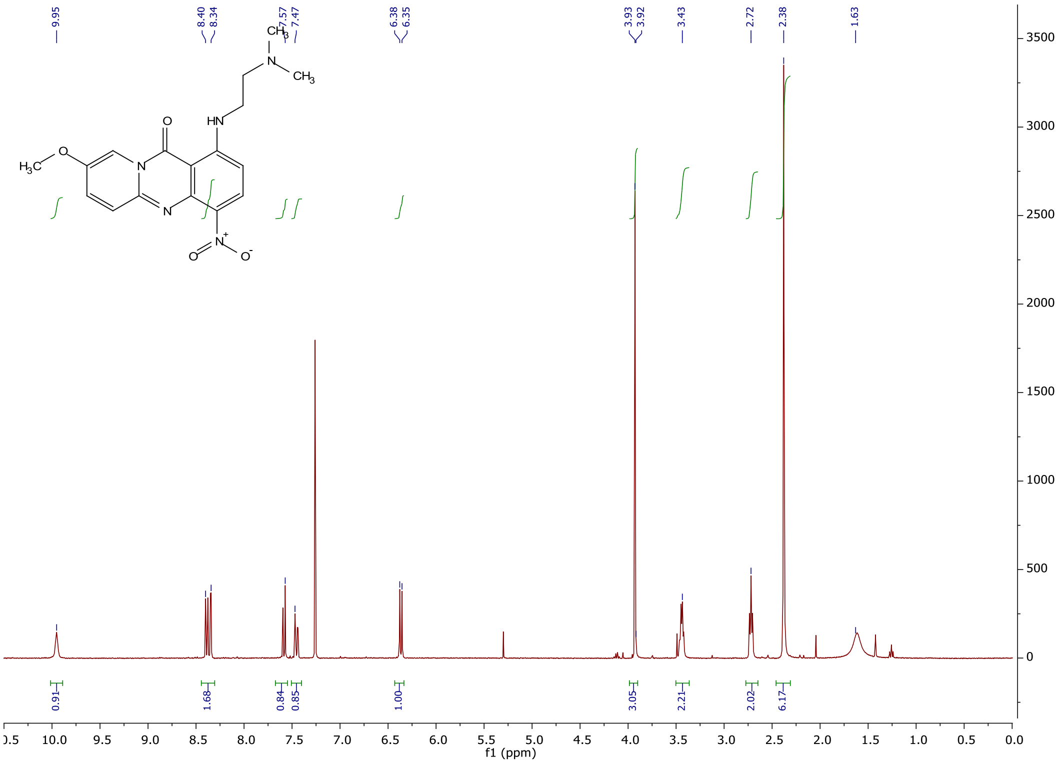

Figure S13: ^{1}H NMR spectrum of 15

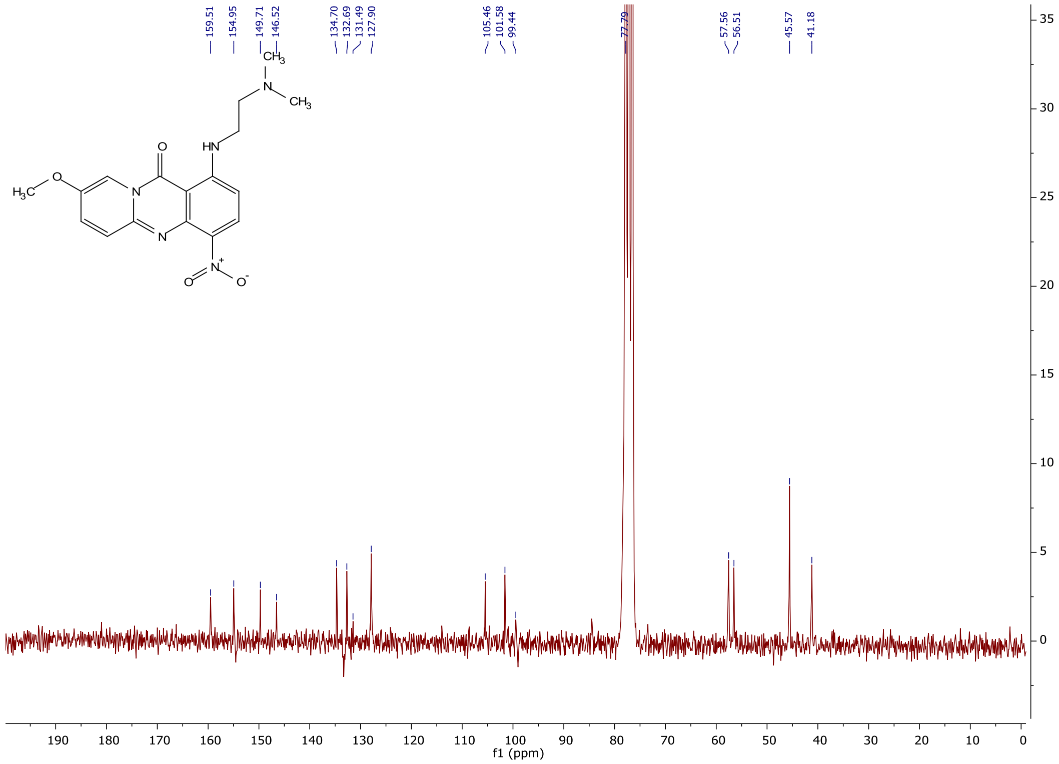

Figure S14: ^{13}C NMR spectrum of 15

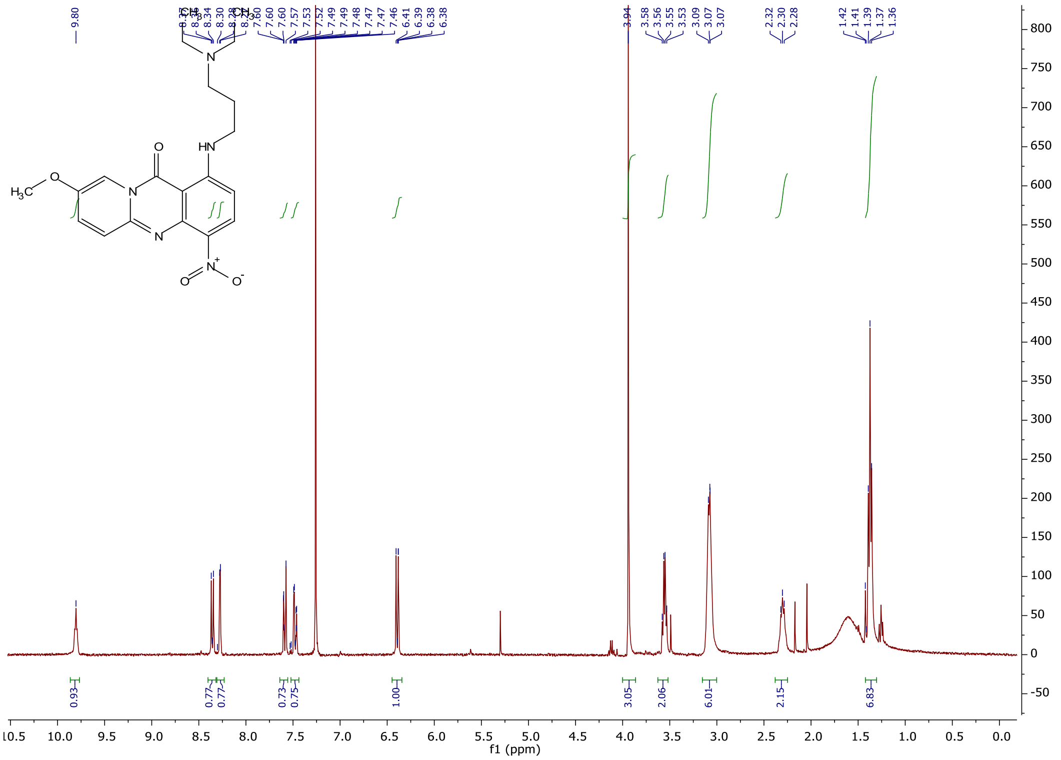

Figure S15: ^{1}H NMR spectrum of 16

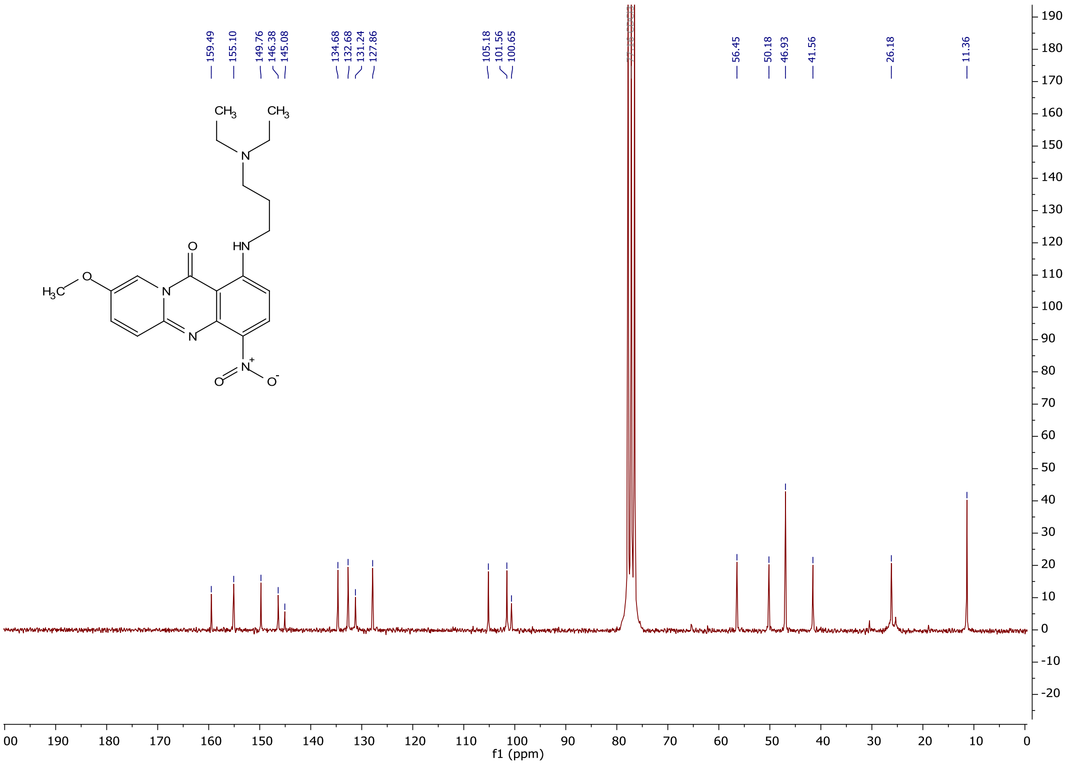

Figure S16: ^{13}C NMR spectrum of 16

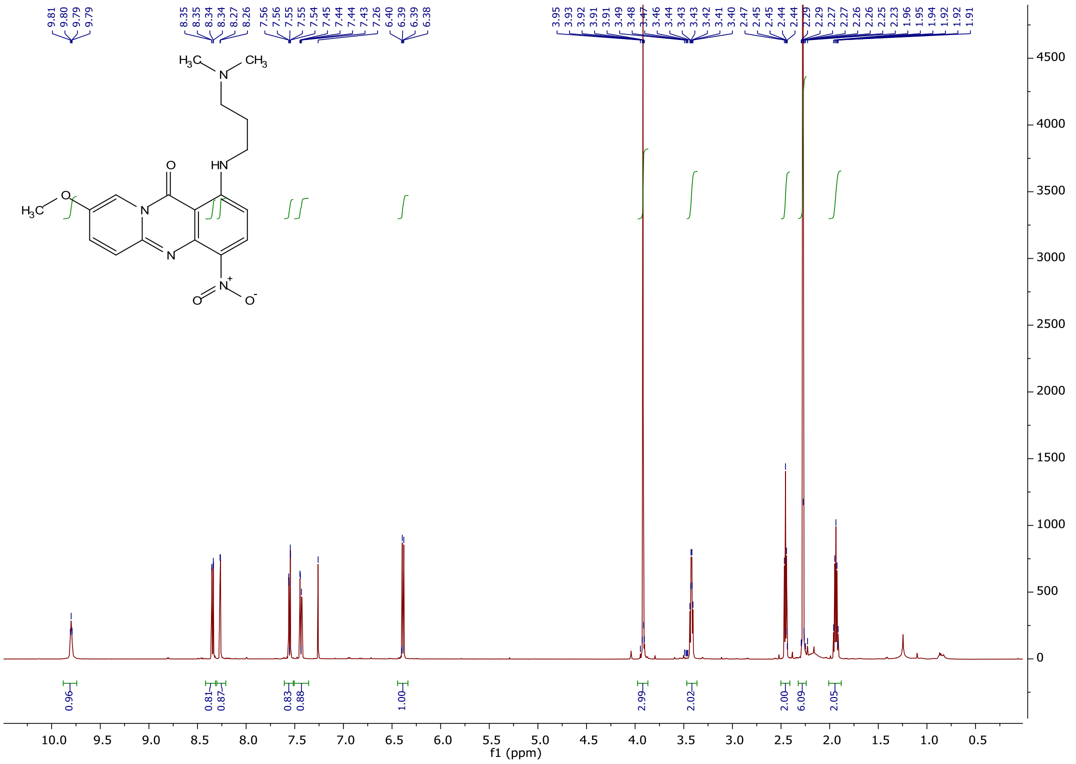

Figure S17: ^{1}H NMR spectrum of 17

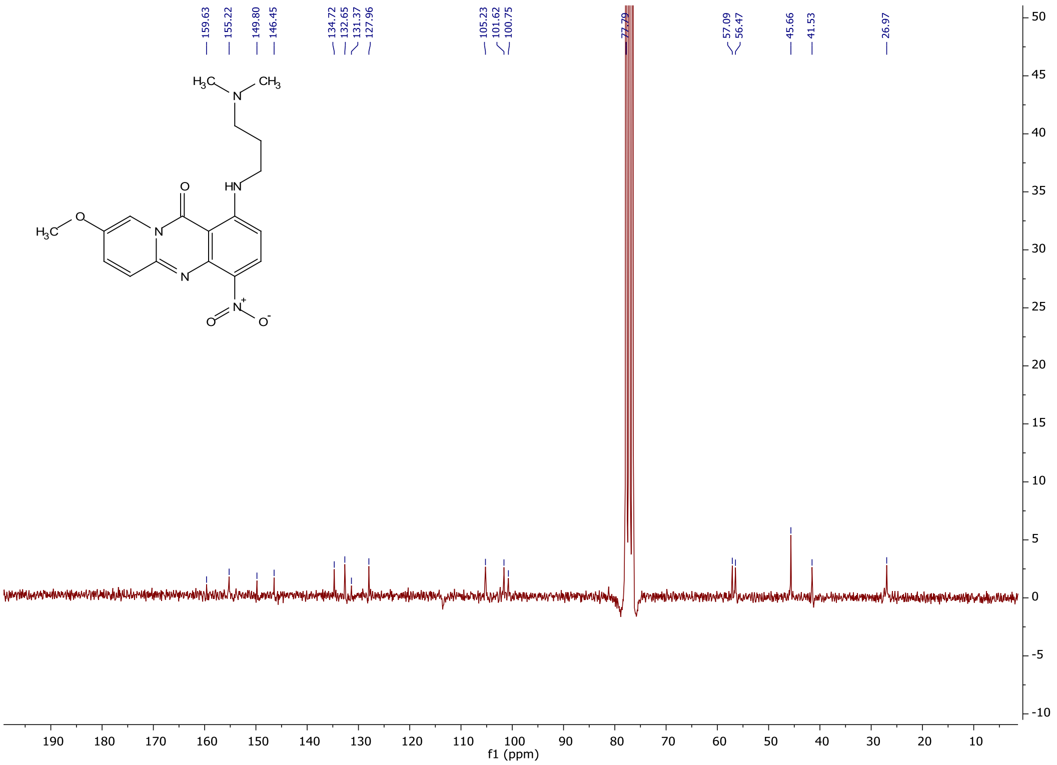

Figure S18: ^{13}C NMR spectrum of 17

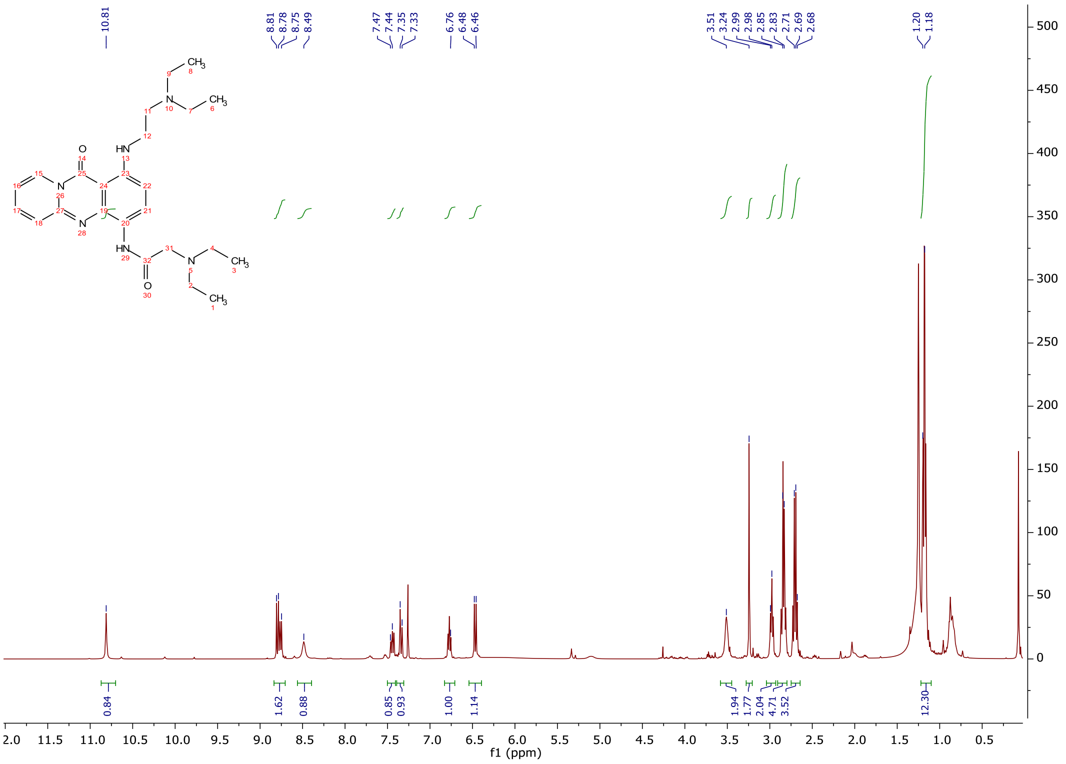

Figure S19: ^{1}H NMR spectrum of 34

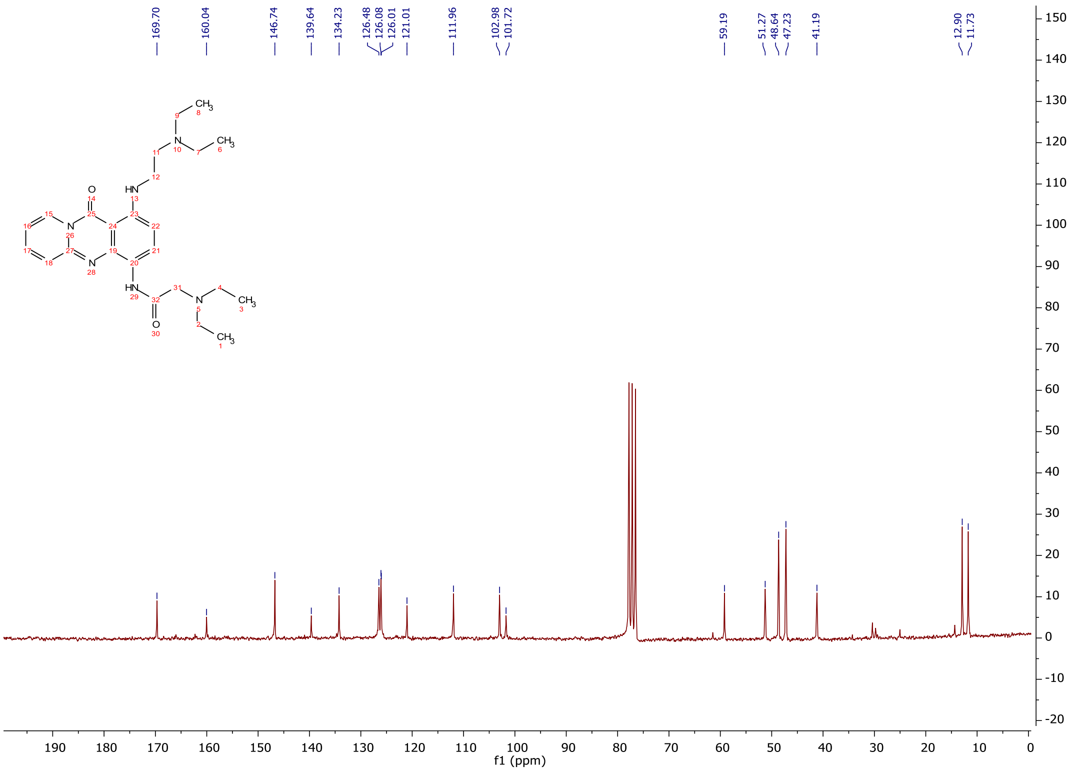

Figure S20: ^{13}C NMR spectrum of 34

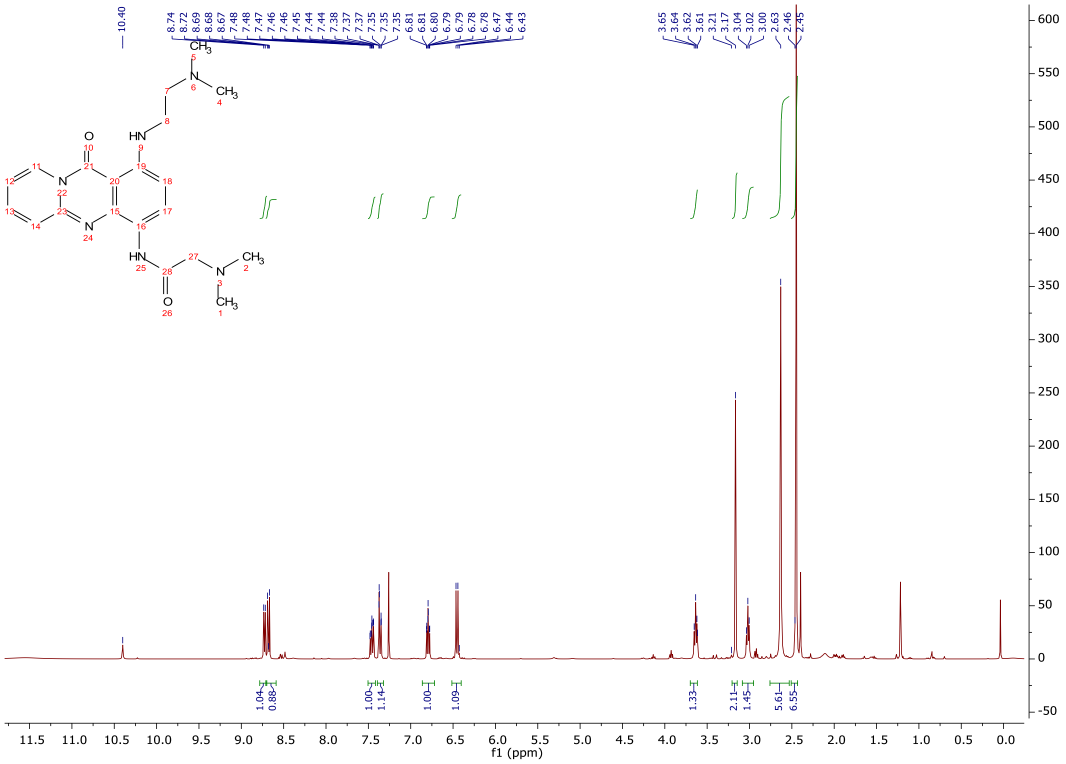

Figure S21: ^{1}H NMR spectrum of 35

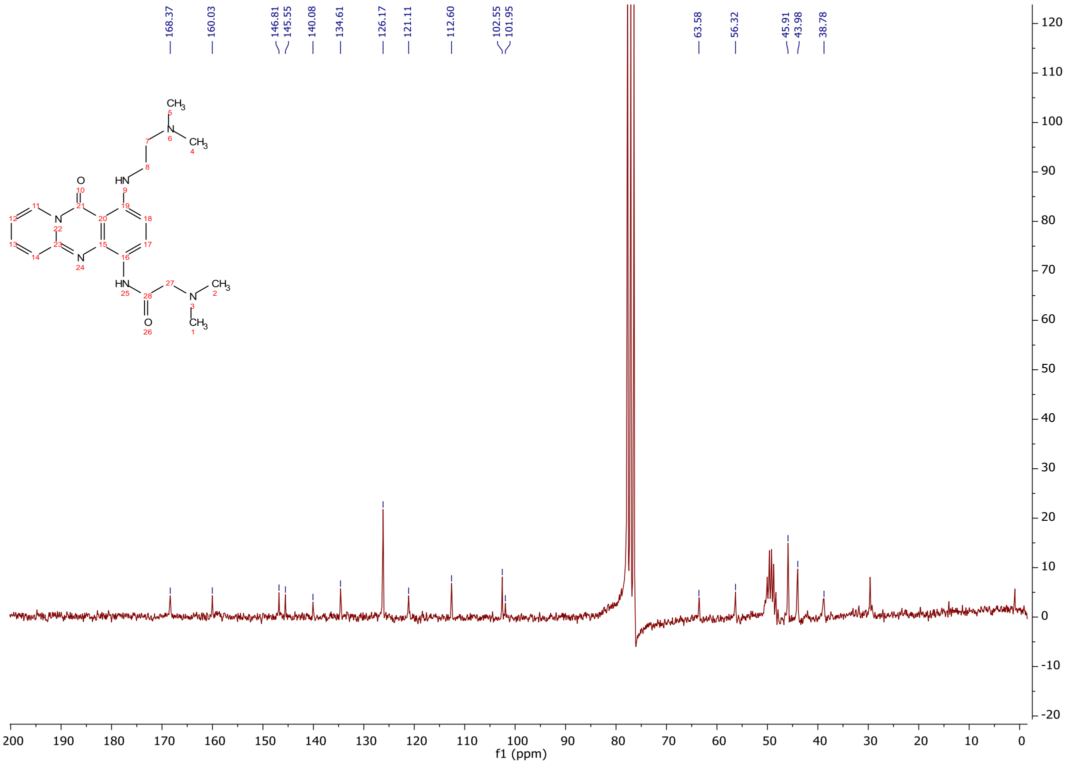

Figure S22: ^{13}C NMR spectrum of 35

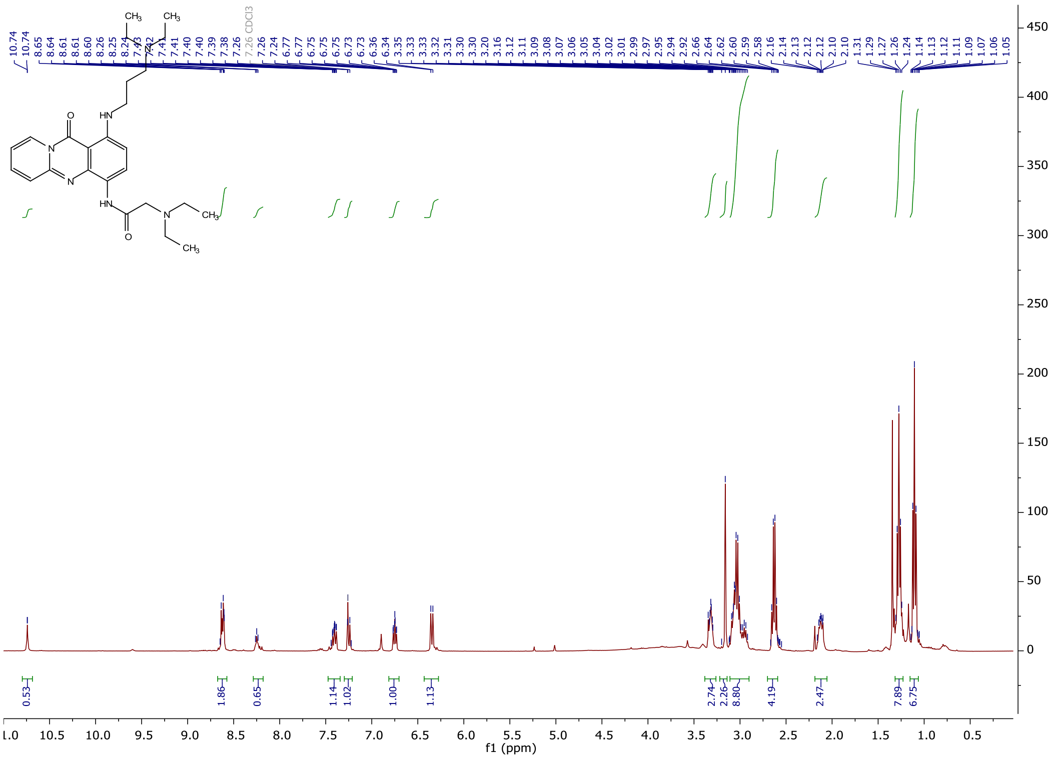

Figure S23: ^{1}H NMR spectrum of 36

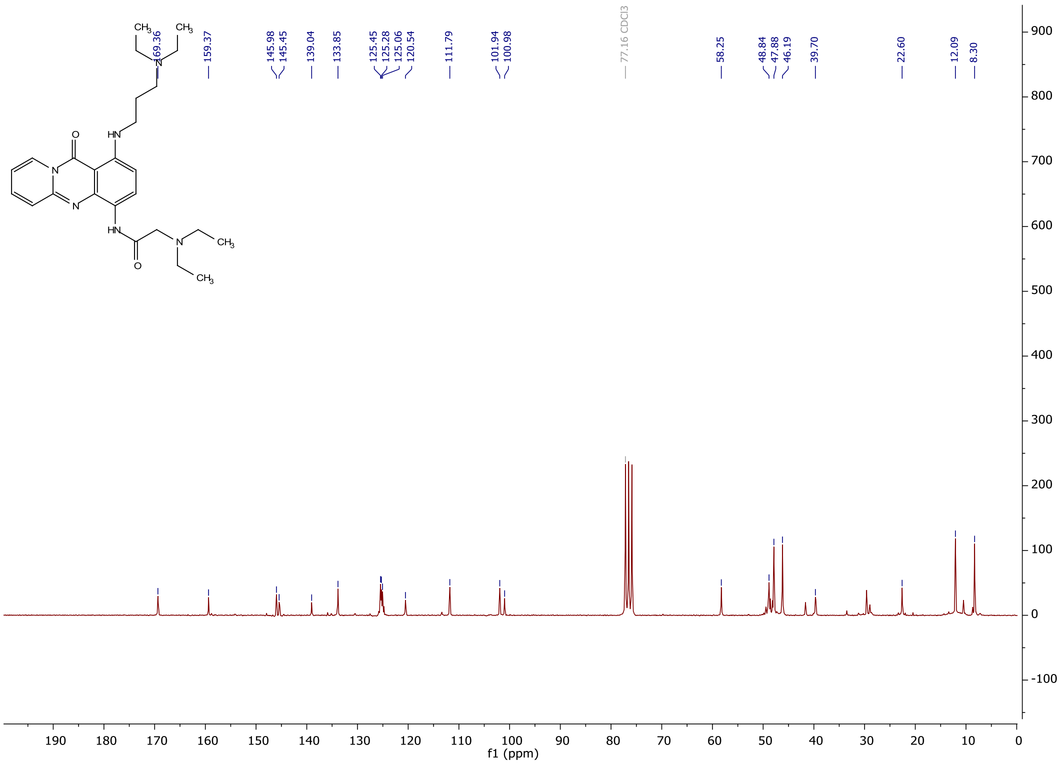

Figure S24: ^{13}C NMR spectrum of 36

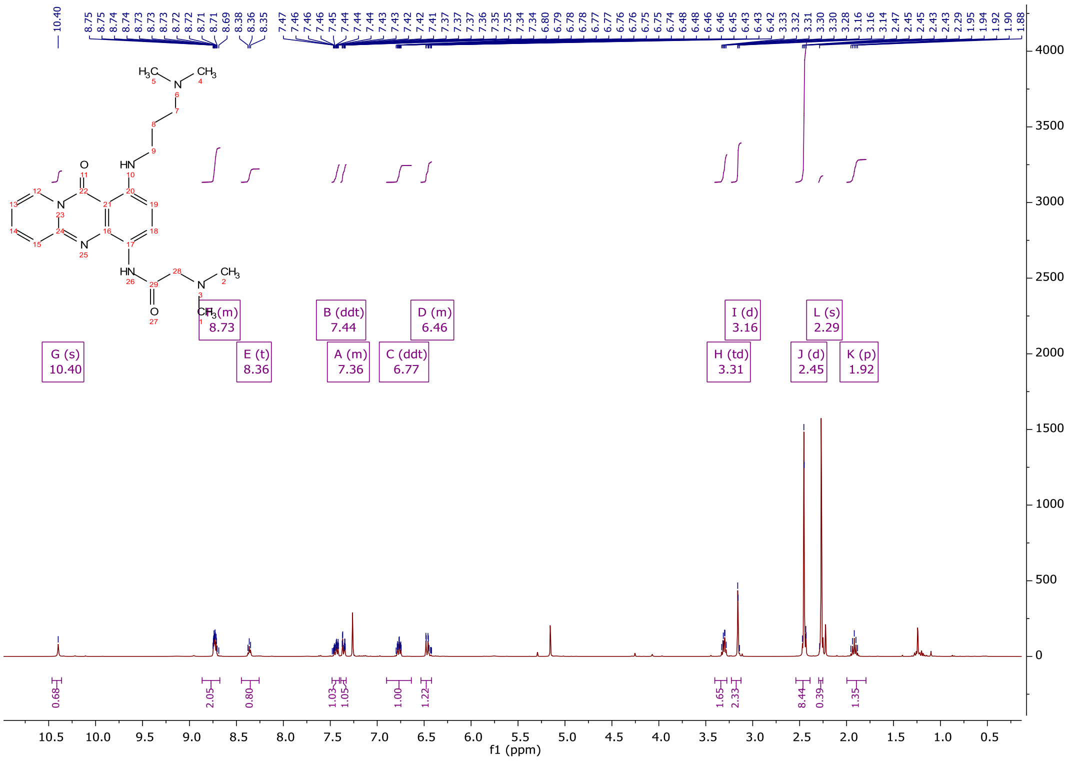

Figure S25: ^{1}H NMR spectrum of 37

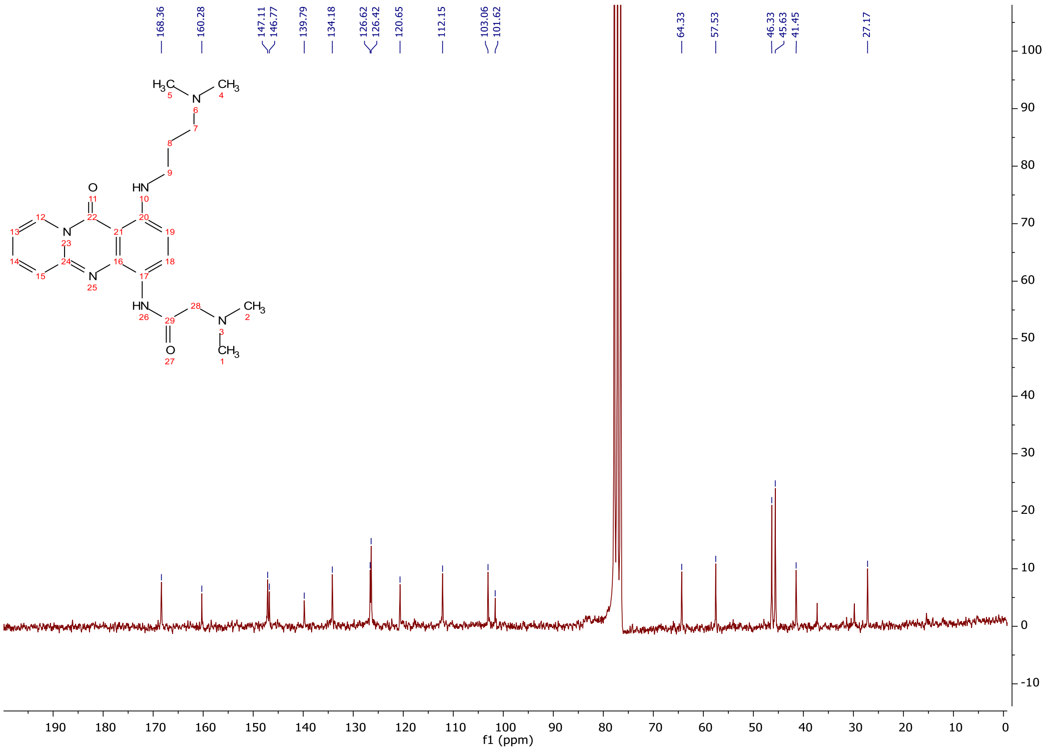

Figure S26: ^{13}C NMR spectrum of 37

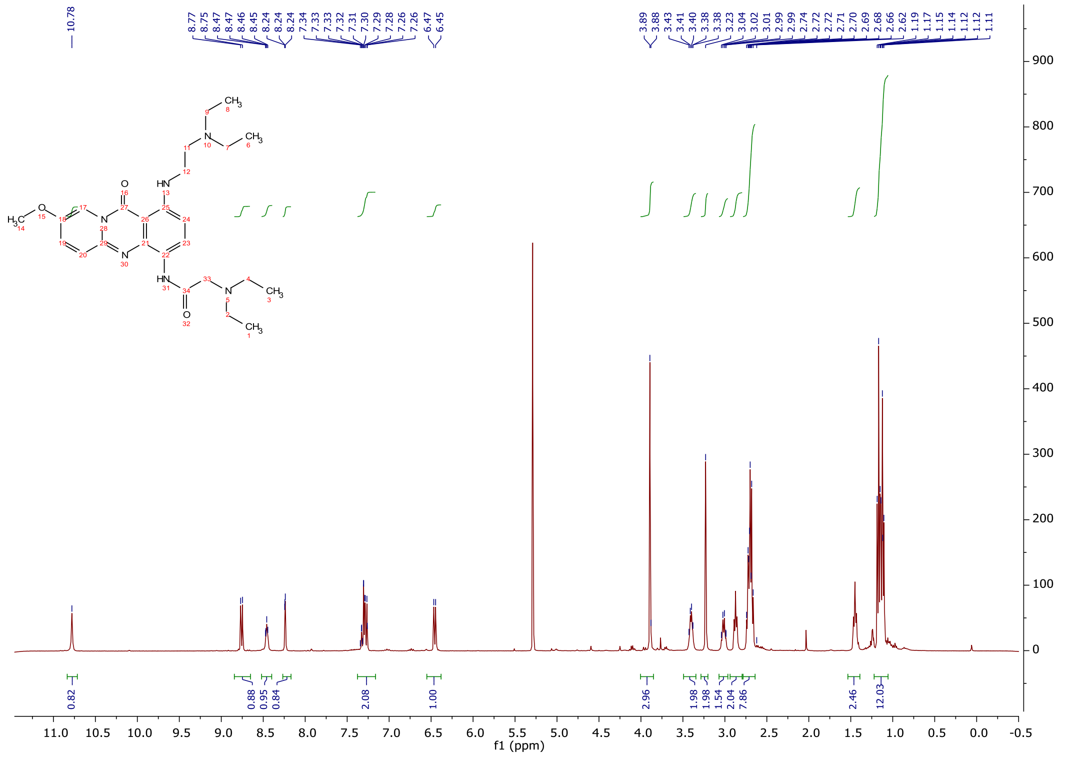

Figure S27: ^{1}H NMR spectrum of 38

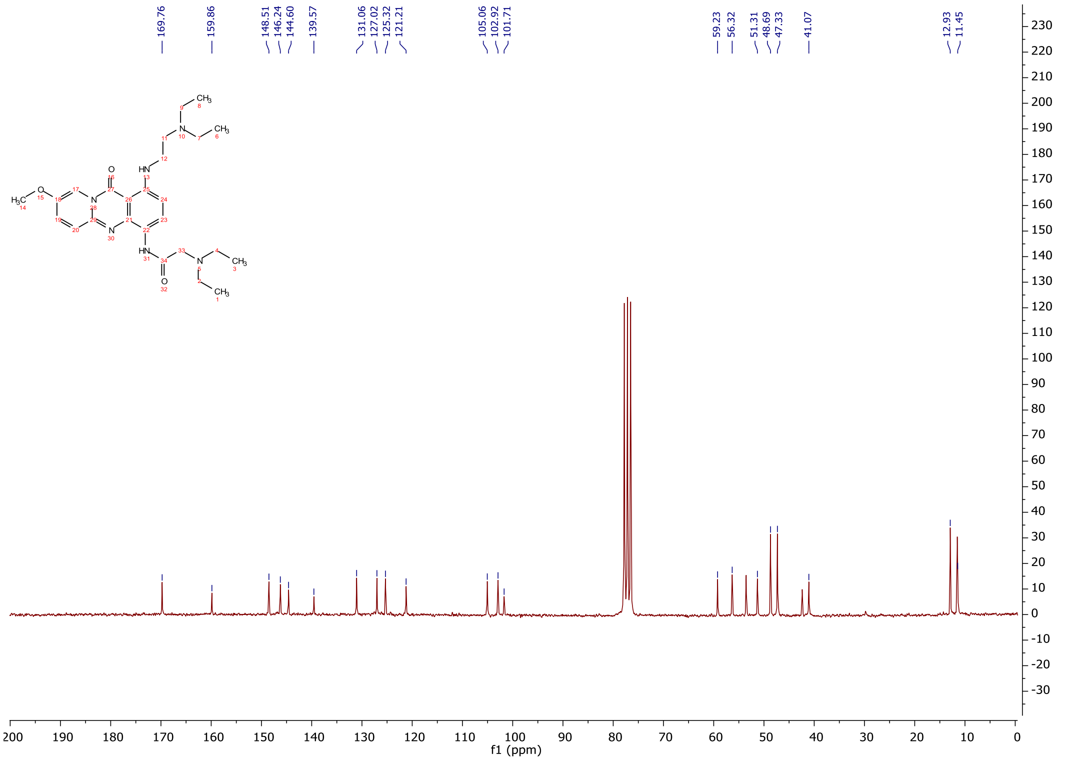

Figure S28: ^{13}C NMR spectrum of 38

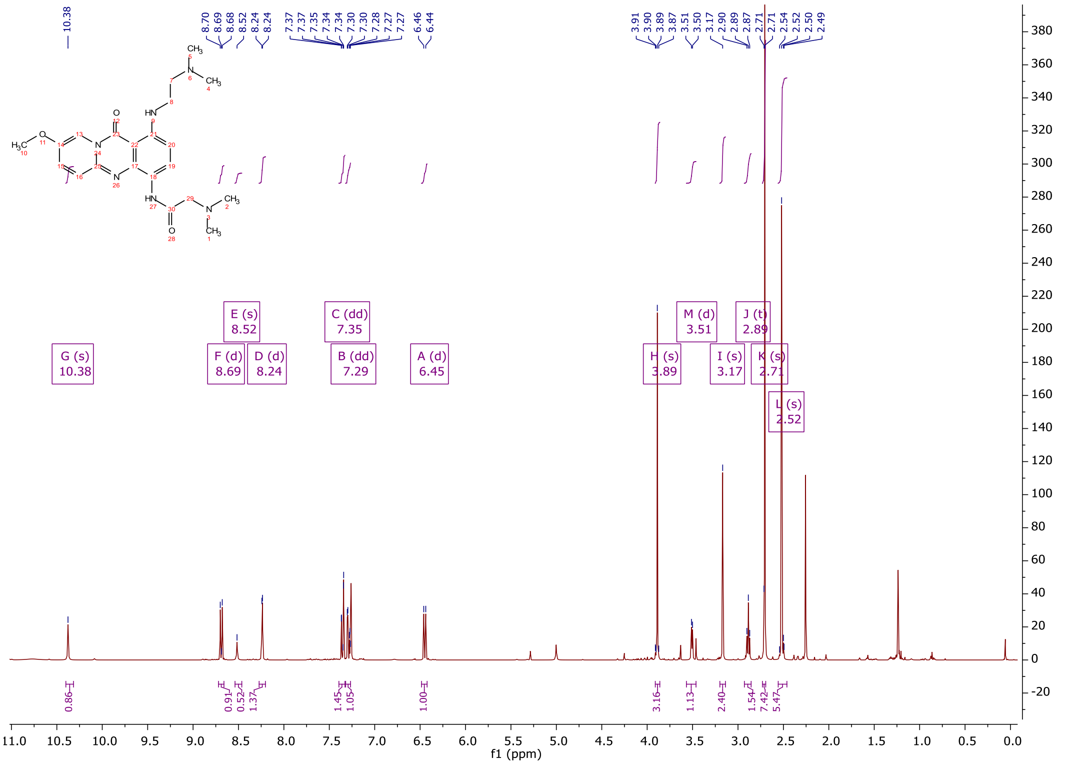

Figure S29: ^{1}H NMR spectrum of 39

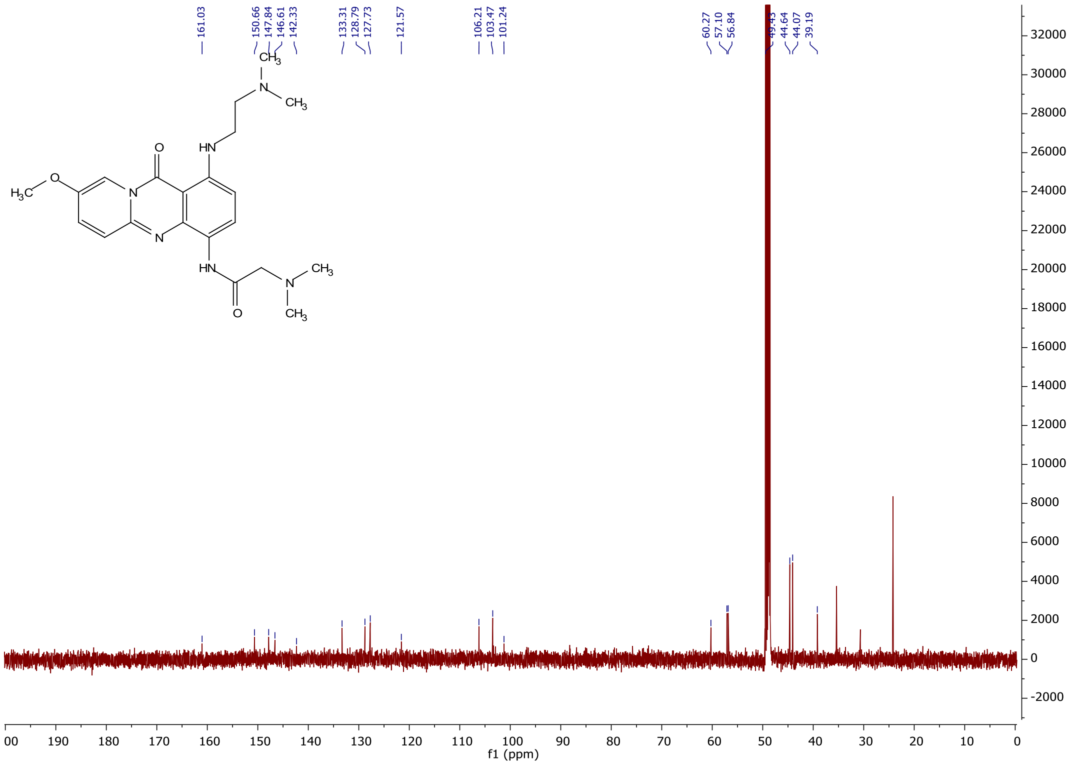

Figure S30: ^{13}C NMR spectrum of 39

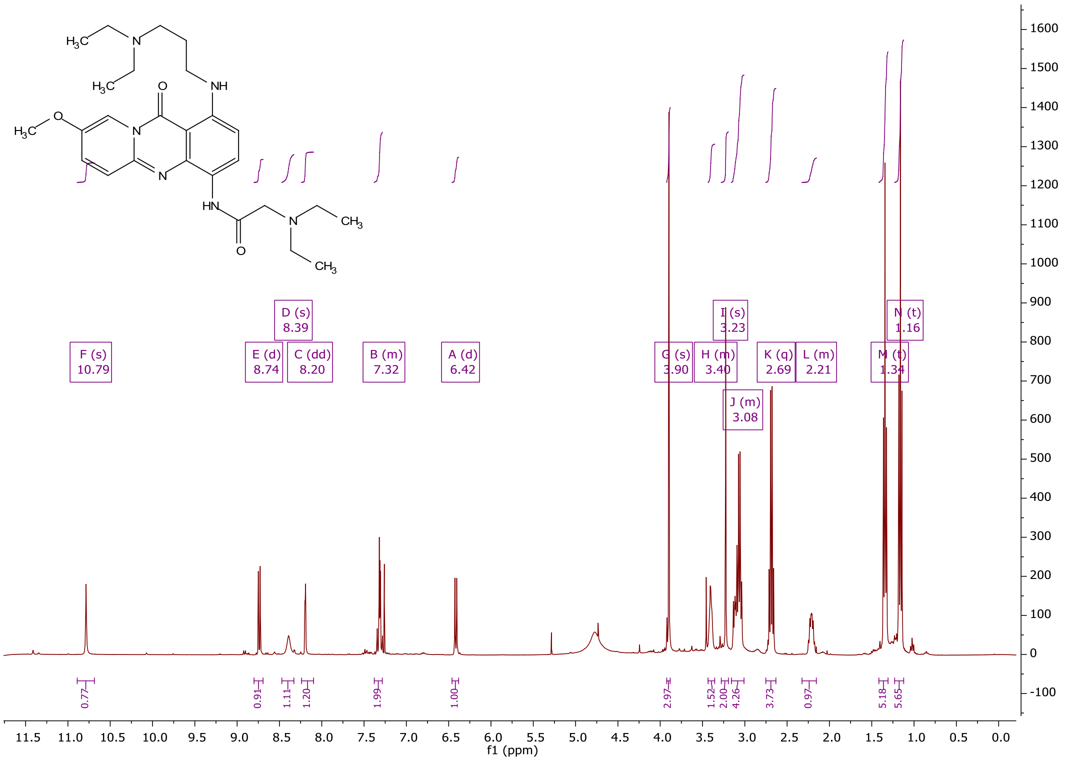

Figure S31: 1H NMR spectrum of 40

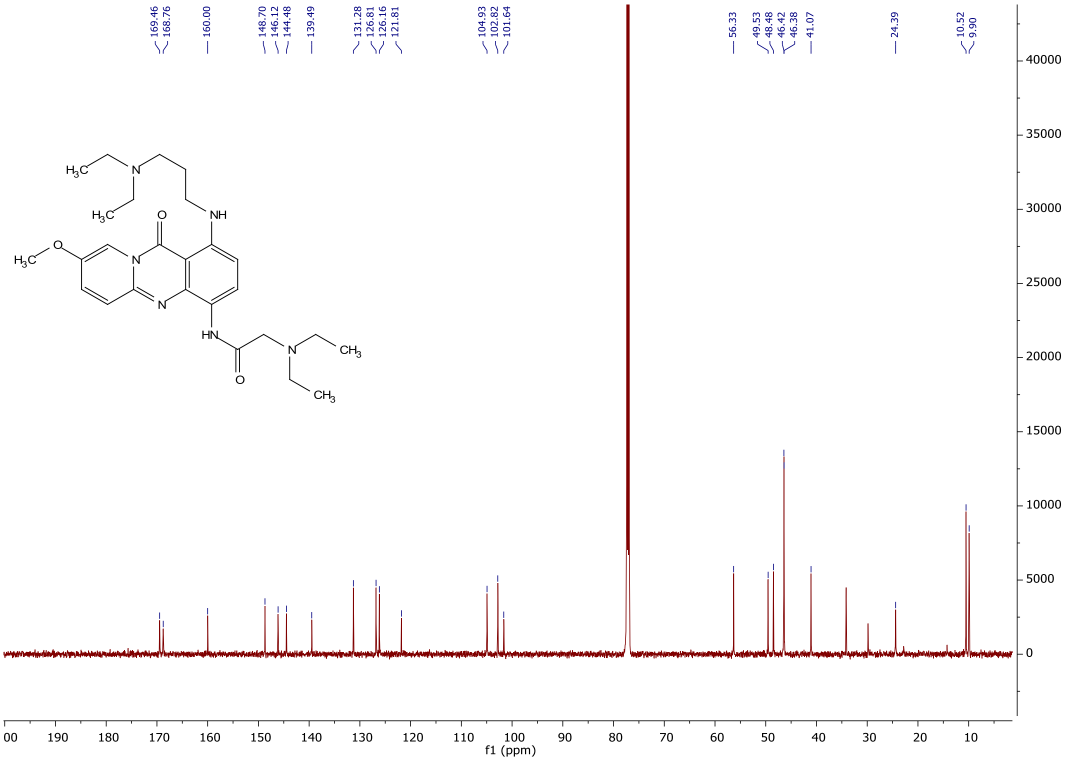

Figure S32: 13C NMR spectrum of 40

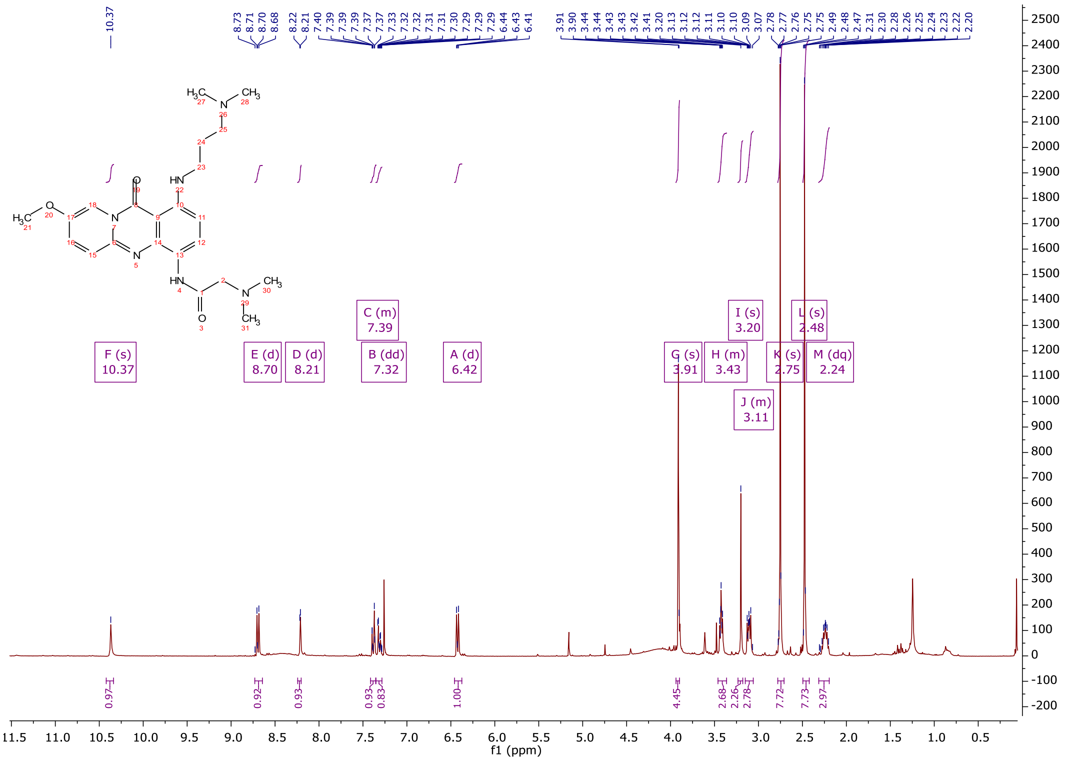

Figure S33: ^{1}H NMR spectrum of 41

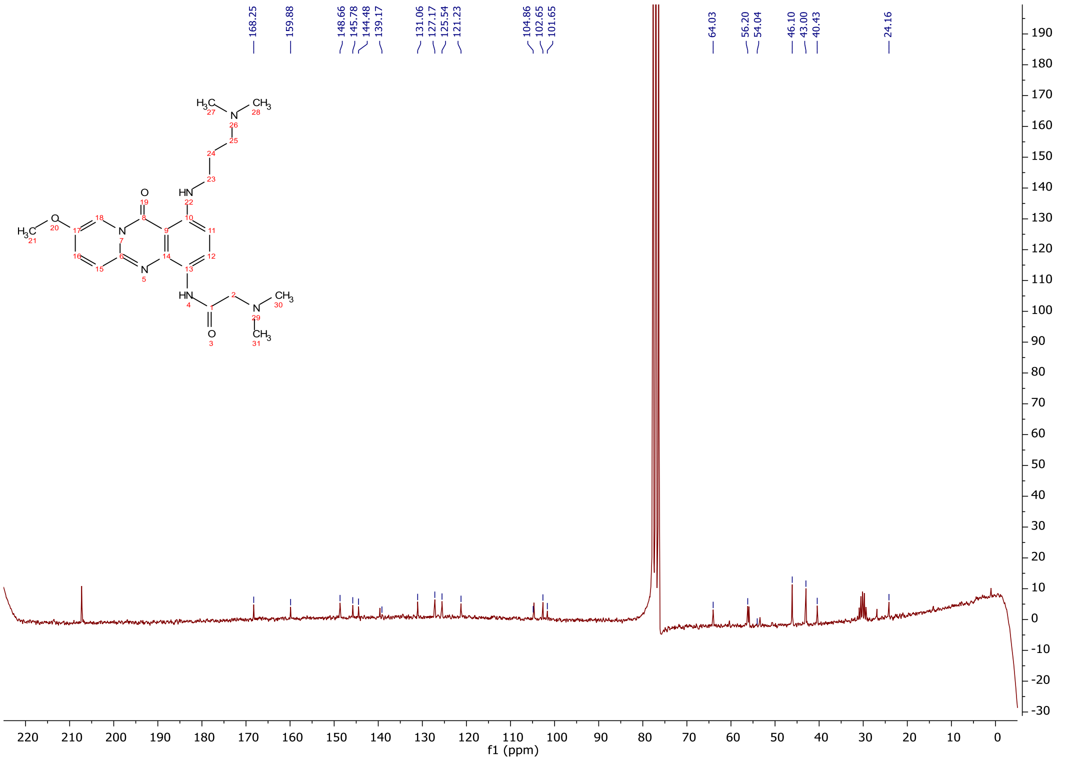

Figure S34: [13C] NMR spectrum of 41

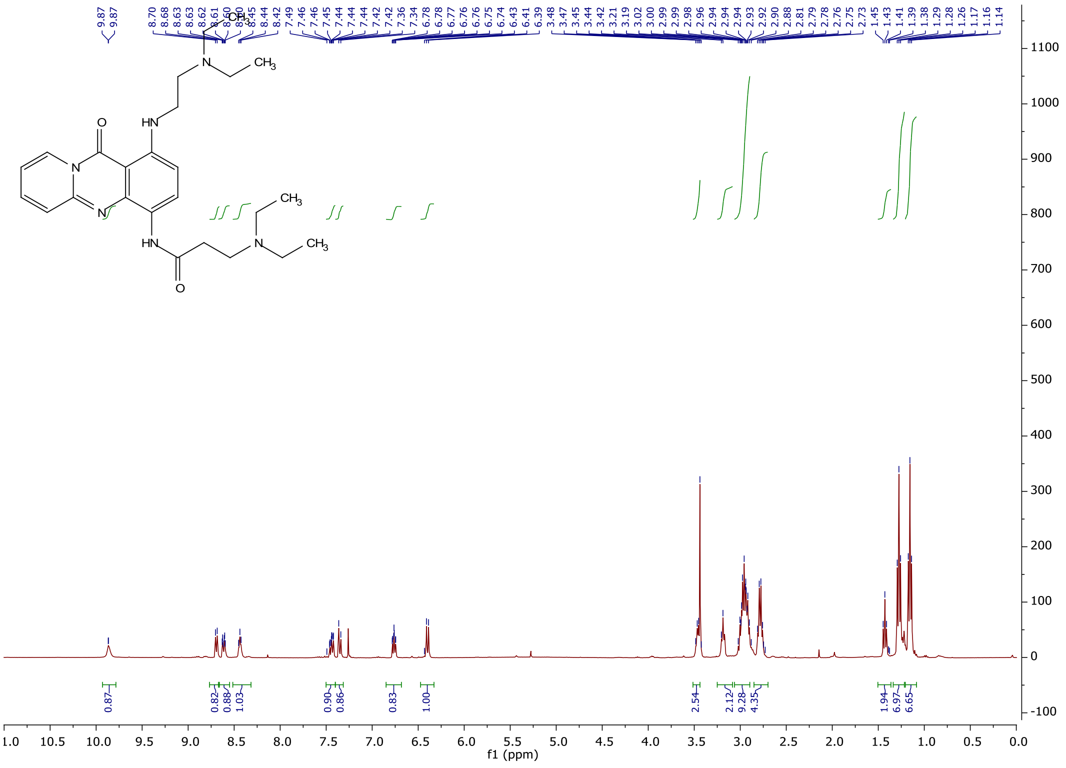

Figure S35: ^{1}H NMR spectrum of 50

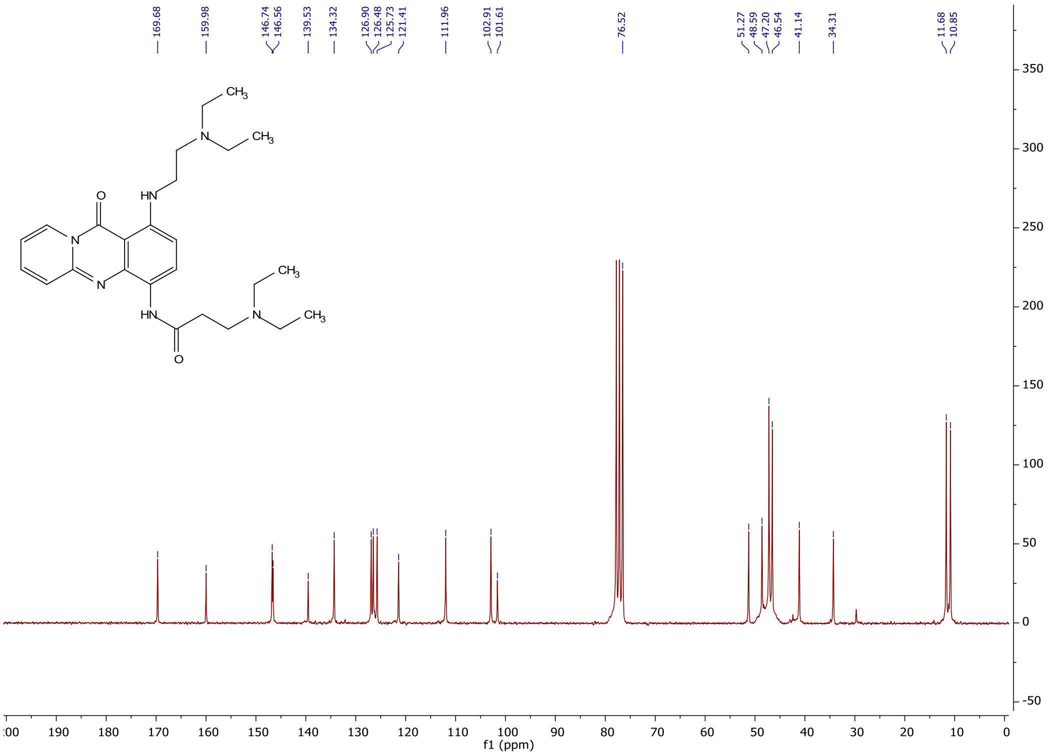

Figure S36: ^{13}C NMR spectrum of 50

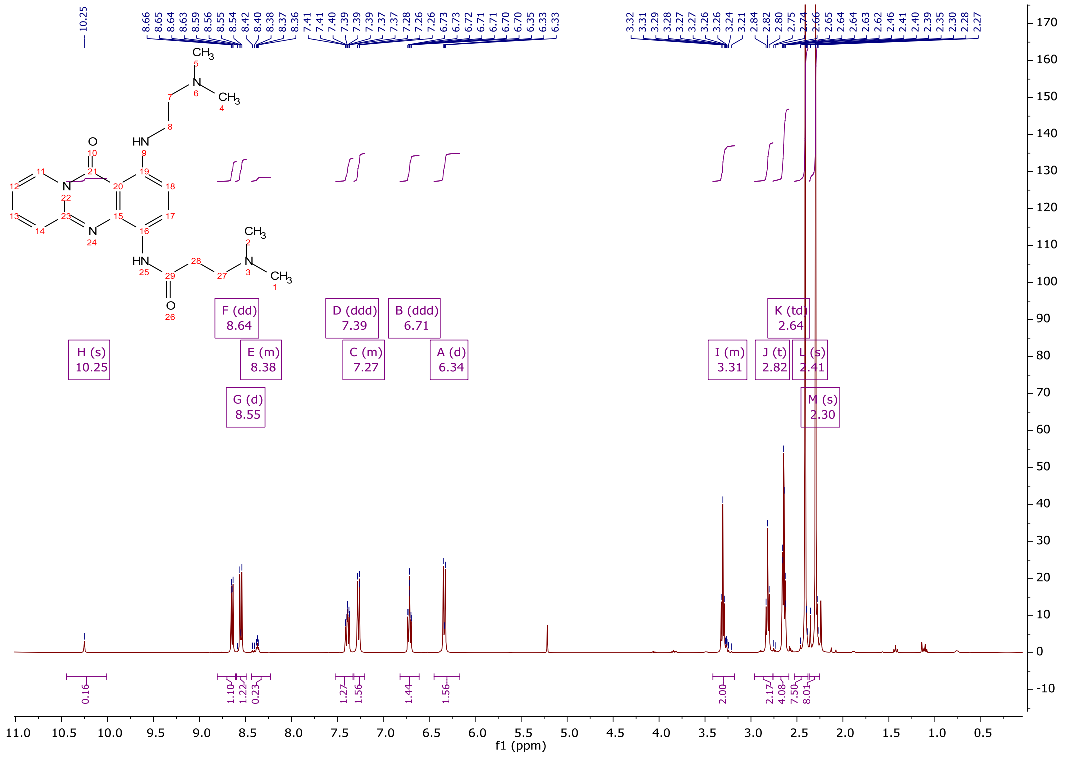

Figure S37: ^{1}H NMR spectrum of 51

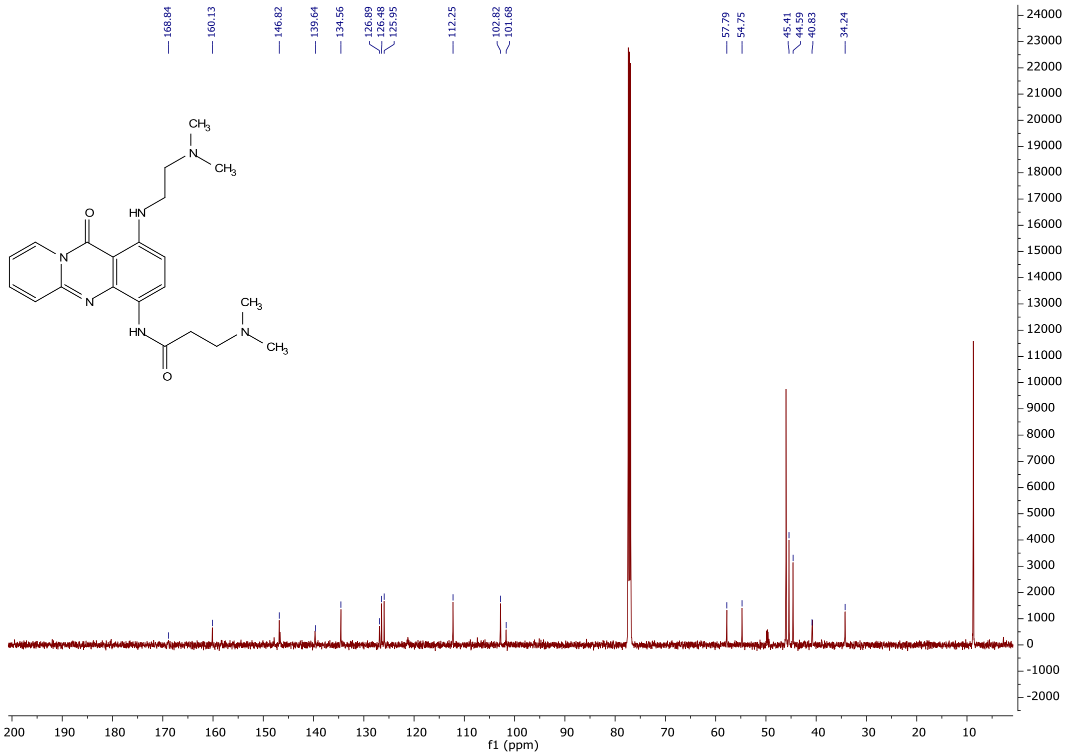

Figure S38: ^{13}C NMR spectrum of 51

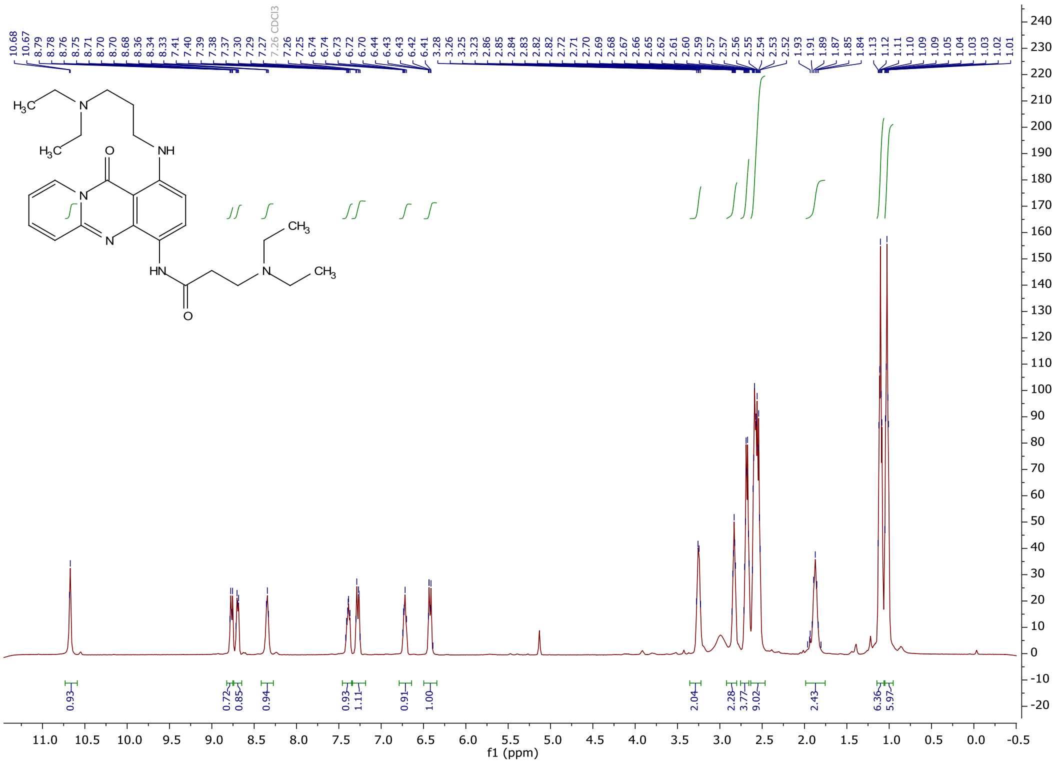

Figure S39: ^{1}H NMR spectrum of 52

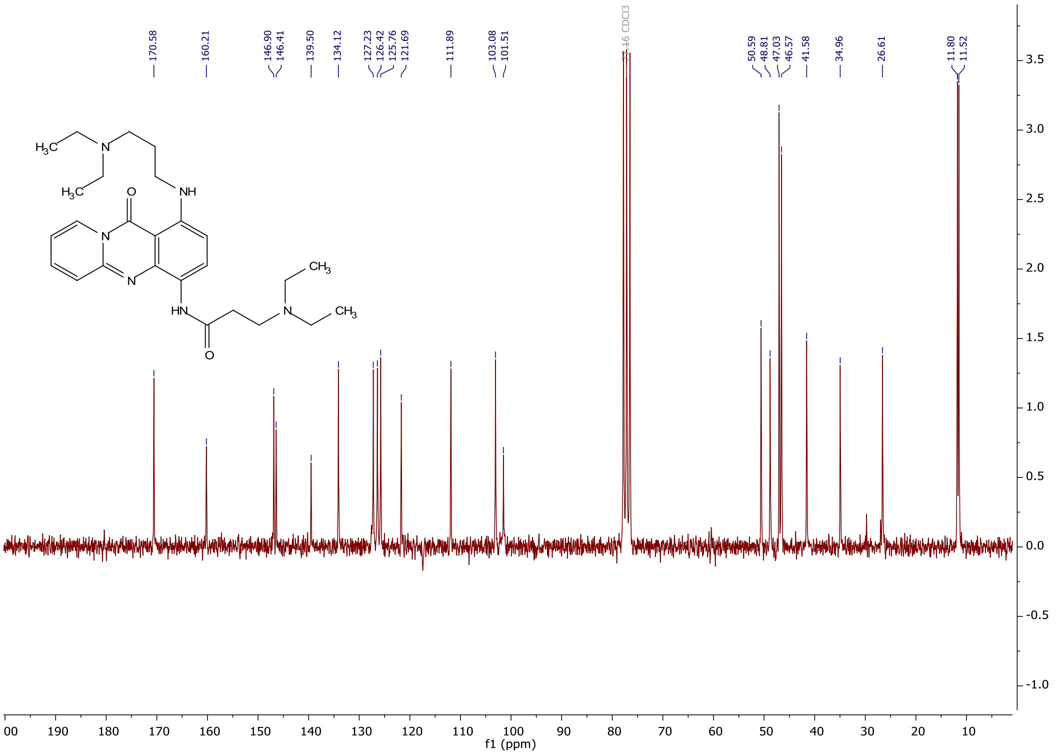

Figure S40: ^{13}C NMR spectrum of 52

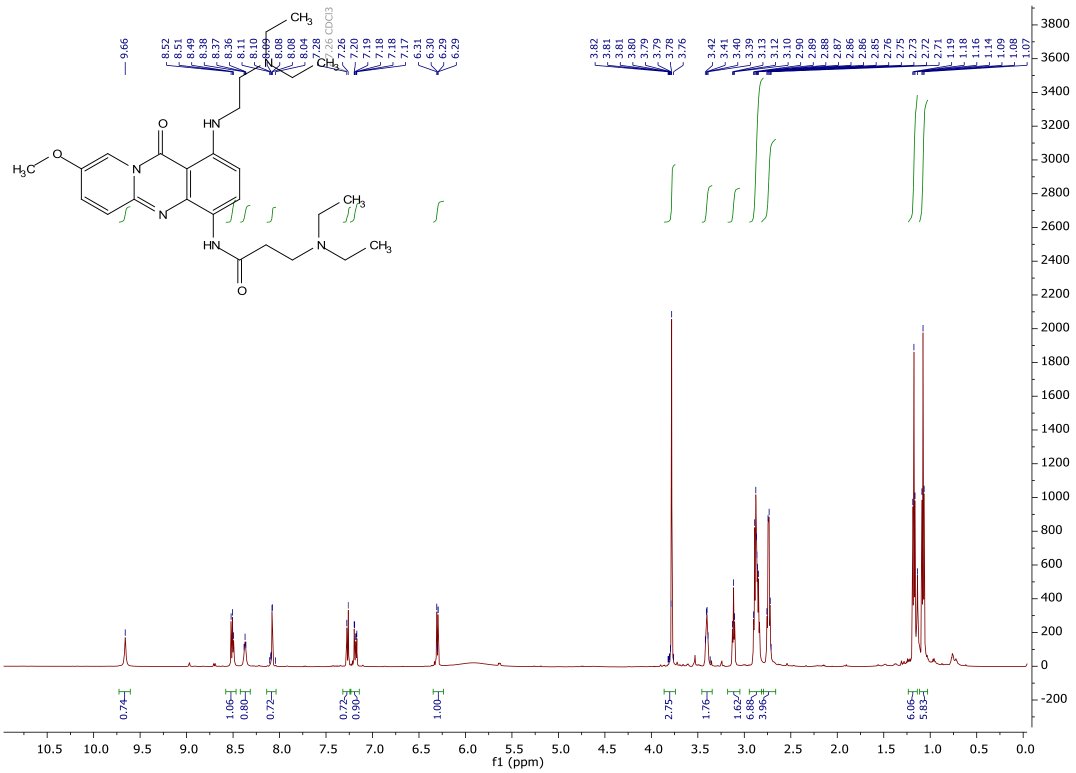

Figure S41: ^{1}H NMR spectrum of 54

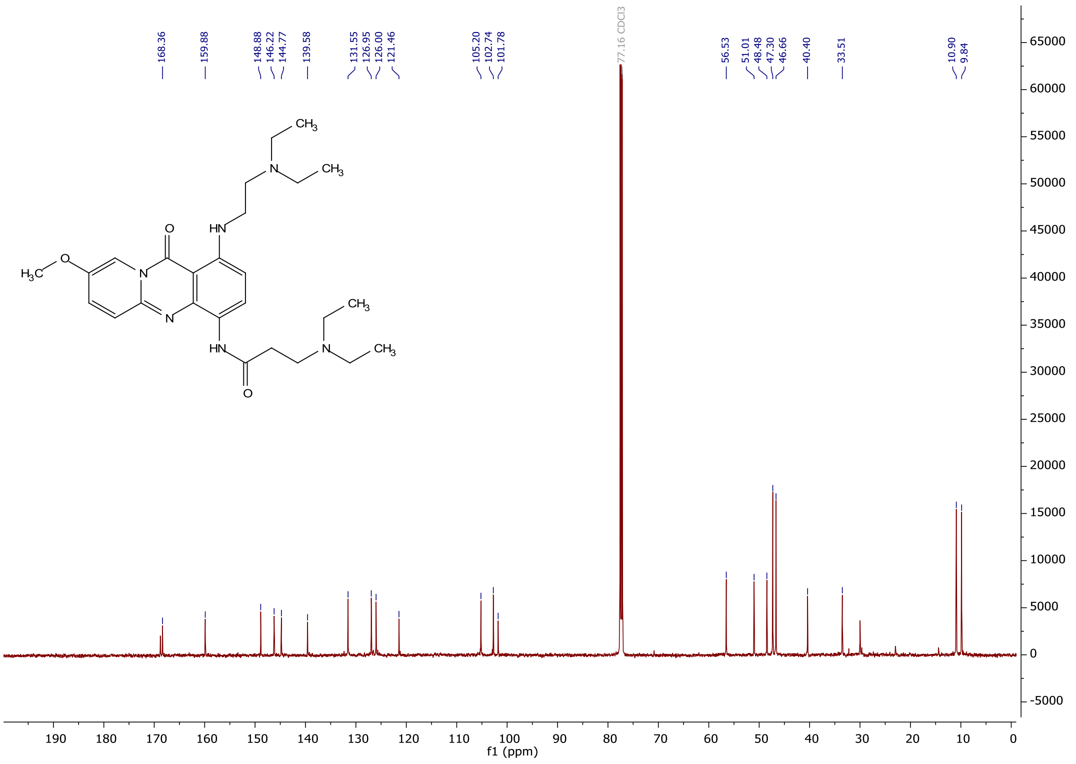

Figure S42: ^{13}C NMR spectrum of 54

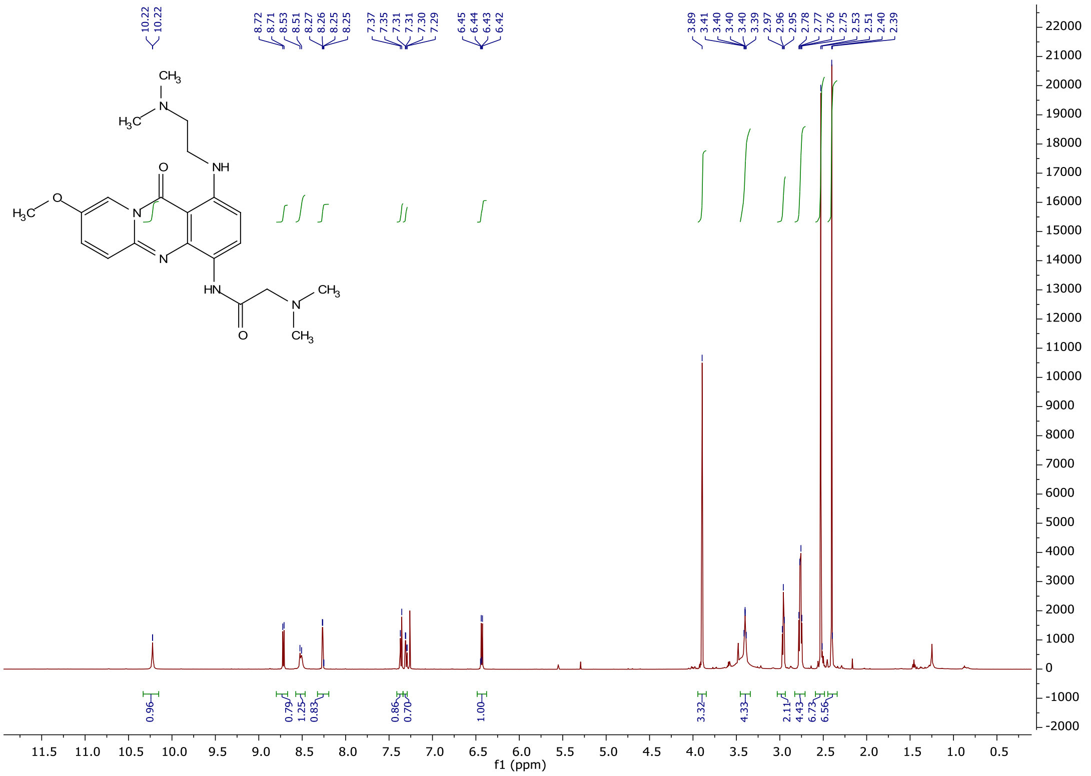

Figure S43: ^{1}H NMR spectrum of 55

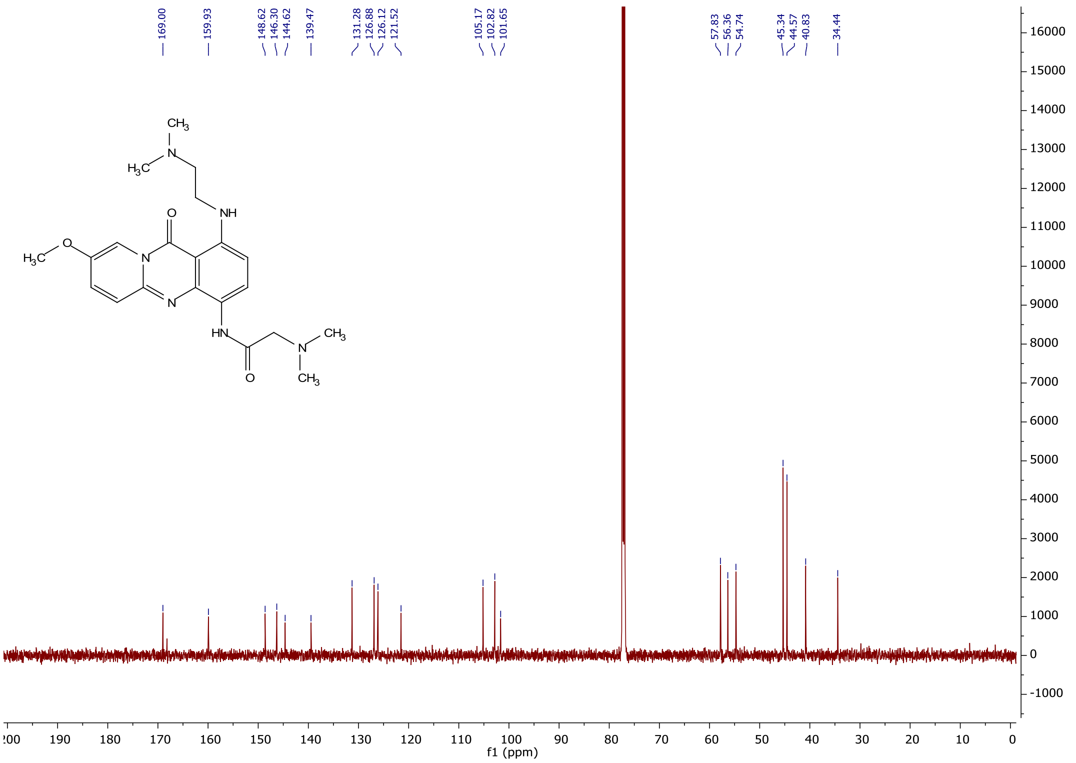

Figure S44: ^{13}C NMR spectrum of 55

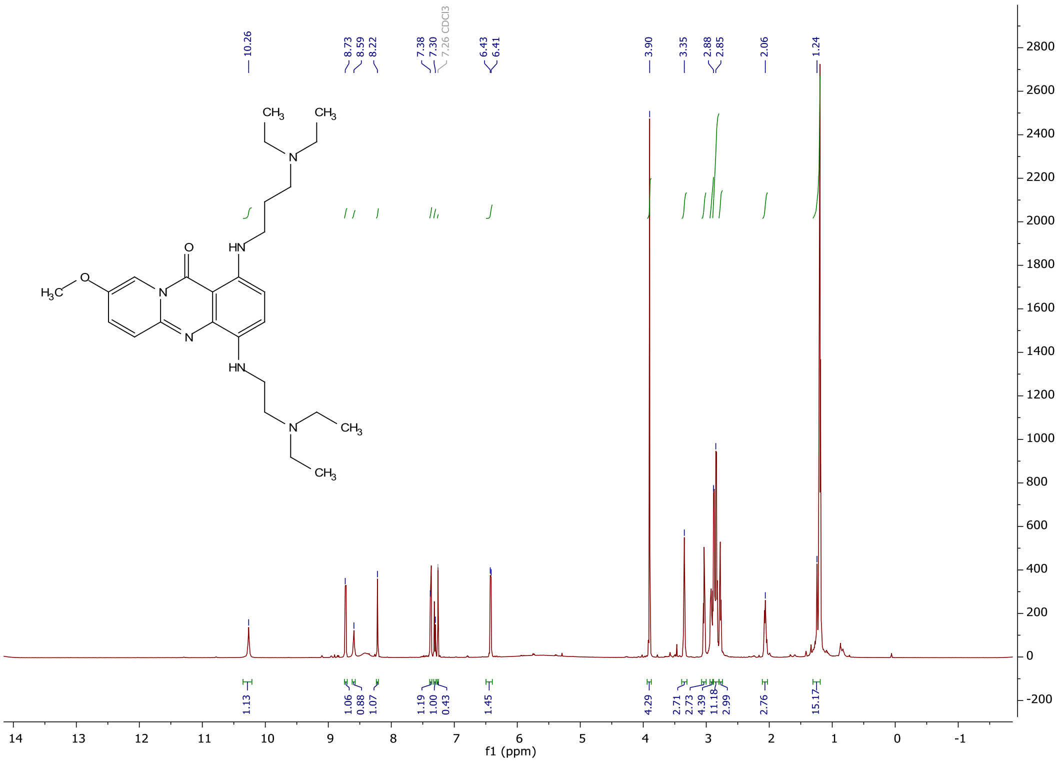

Figure S45: ^{1}H NMR spectrum of 56

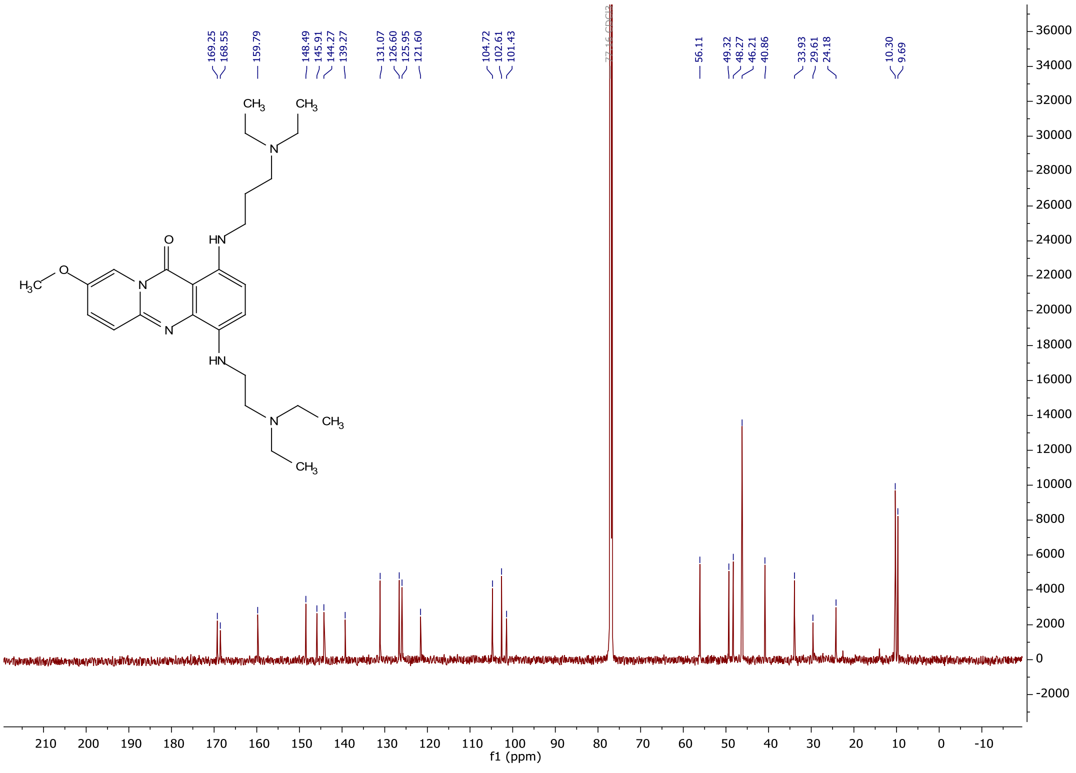

Figure S46: ^{13}C NMR spectrum of 56

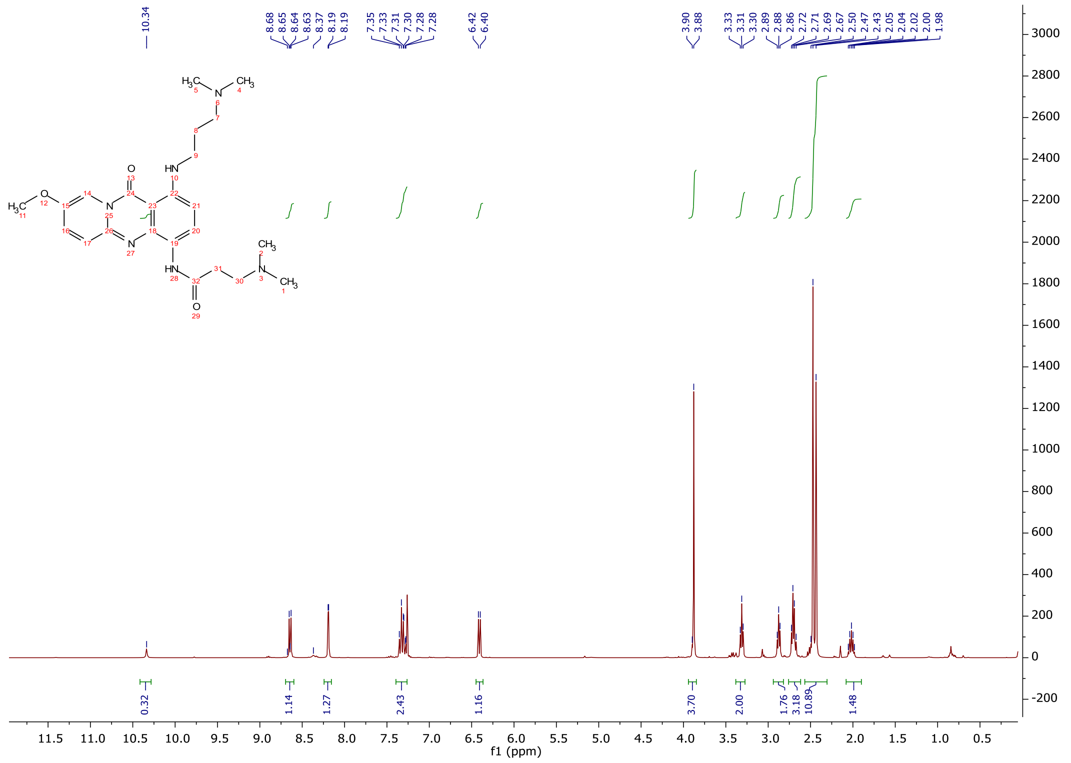

Figure S47: ^{1}H NMR spectrum of 57

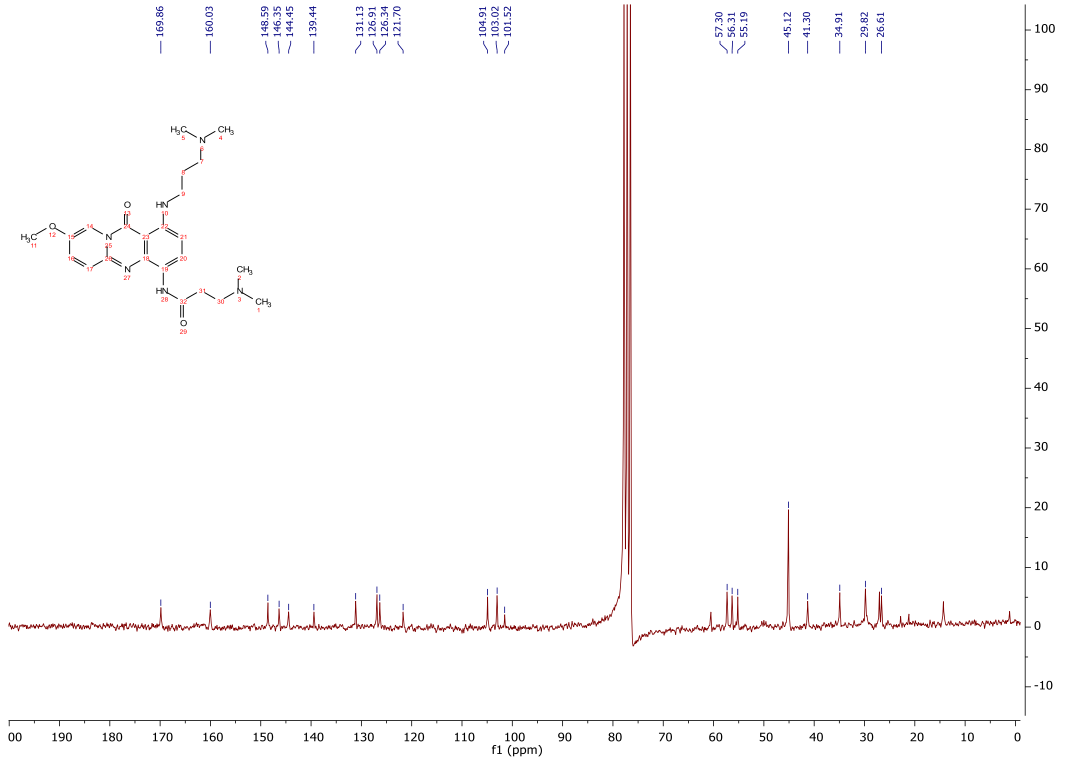

Figure S48: 13C NMR spectrum of 57
